# Phenol‐Rich Carbon Dots as Metal‐Free Nano‐Photocatalysts for [3 + 2] Cycloaddition Reactions

**DOI:** 10.1002/cssc.202500521

**Published:** 2025-04-17

**Authors:** Martina Mamone, Giuseppe Gentile, Maurizio Prato, Giacomo Filippini

**Affiliations:** ^1^ Department of Chemical and Pharmaceutical Sciences INSTM UdR Trieste University of Trieste Via Licio Giorgieri 1 34127 Trieste Italy; ^2^ Centre for Cooperative Research in Biomaterials (CIC BiomaGUNE) Basque Research and Technology Alliance (BRTA) Paseo de Miramón 194 20014 Donostia‐San Sebastián Spain; ^3^ Basque Foundation for Science Ikerbasque Plaza Euskadi 5 48013 Bilbao Spain

**Keywords:** carbon dots, cycloaddition reactions, nanomaterials, nano‐organocatalyses, phenols, photoredox catalyses, synthetic methods

## Abstract

Carbon dots (CDs) are unique carbon‐based nanoparticles with potential applications in the field of photocatalysis. In this context, the proper selection of precursors and synthetic conditions is of paramount importance when tailoring the photocatalytic features of the resulting nanomaterials. Herein, a novel *bottom‐up* methodology has been developed for the preparation of phenol‐rich CDs (*Ph*‐CDs) that allowed us to capitalize on the excellent photoredox properties of surface phenolate anions that can be obtained upon deprotonation of *Ph*‐CDs. Specifically, in this study, *Ph*‐CDs are used in combination with a suitable base and an organocatalyst, namely the Schreiner thiourea (**A**), to drive valuable [3 + 2] photocycloaddition reactions between suitable cyclopropyl ketones **1** and unsaturated hydrocarbons **2**. Mechanistic studies show the key role played by **A** in the transformation. In fact, this organocatalyst may not only activate the carbonyl moiety of **1** but also stabilize the formation and enhance the photocatalytic performances of phenolate anions on the nanoparticle surfaces. Remarkably, this photocatalytic transformation provides a wide variety of densely functionalized five‐membered rings **3** (*17 examples, up to 99% yield*) under mild operative conditions. Lastly, it is demonstrated that *Ph*‐CDs can be easily recovered and reused up to three times without any significant drop in yield.

## Graphical abstract

Phenol‐rich carbon dots can be effectively used, in combination with a proper base and the Schreiner thiourea (**A**), as nano‐photocatalysts to drive synthetically relevant [3 + 2] cycloaddition reactions between cyclopropyl ketones **1** and unsaturated hydrocarbons **2**.

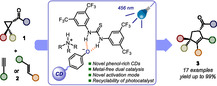

## 1 Introduction

**Figure 1:**
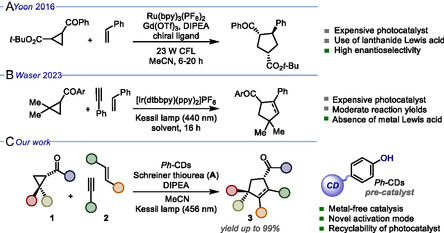
Overview of photocatalytic [3 + 2] cycloaddition strategies. A) Previous work on the photocatalytic [3 + 2] cycloaddition by Yoon et al. B) Recent photocatalytic route for the synthesis of saturated and unsaturated five‐membered cycles by Waser et al. C) Our work: nano‐photocatalytic [3 + 2] cycloaddition using *Ph*‐CDs in combination with **A**. CFL = compact fluorescent lamp. DIPEA: *N*,*N*‐diisopropylethylamine.

The field of organic photocatalysis has grown enormously in recent years.^[^ ^1^, ^2^, ^3^ ^]^ Photocatalysis results in unique bond constructions that are not accessible under classical thermal protocols, while reducing waste production and energy consumption.^[^ ^4^, ^5^ ^]^ In this context, photoredox catalysis, involving the light‐induced movement of one electron from or to a substrate, has become a well‐established tool for the direct functionalization of organic molecules under mild operative conditions.^[^ ^6^, ^7^ ^]^ However, the most used visible‐light photoredox catalysts (PCs) are typically based on expensive and potentially toxic polypyridyl complexes of ruthenium and iridium.^[^ ^8^, ^9^ ^]^ For this reason, a great deal of effort has been devoted to developing more sustainable and effective metal‐free photocatalytic systems. It follows that numerous organic dyes have been recently investigated in photoredox catalysis, all generally characterized by a densely functionalized aromatic core.^[^ ^10^, ^11^, ^12^ ^]^ From these investigations, phenol derivatives have emerged as a new class of metal‐free pre‐PCs capable of driving the synthesis of valuable organic compounds under visible‐light irradiation.^[^ ^13^ ^]^ In fact, the conjugate bases of these pre‐PCs, namely phenolate anions, are both electron‐rich organic intermediates and active chromophores. Thus, these anions can be effectively employed as PCs to produce reactive open shell species from suitable electron‐poor radical precursors either 1) by reaching an electronically excited state upon light absorption or 2) by forming photoactive electron donor–acceptor (EDA) complexes.^[^ ^14^, ^15^, ^16^ ^]^ Nevertheless, it is worth mentioning that, generally, most of the organic PCs are used with relatively high catalytic loading and their use might be limited by tedious multistep preparations, the fact that they cannot be recycled, and instability under certain reaction conditions.^[^ ^17^ ^]^ Thus, progression in this research field would require the design and development of new efficient, inexpensive, safe, readily available, and potentially recyclable PCs. Interestingly, carbon dots (CDs) fulfill all these requirements. CDs are photoactive carbon–based nanoparticles with dimensions below 10 nm which boast outstanding physicochemical properties, leading to their emergence as promising green nano‐photocatalytic systems.^[^ ^18^, ^19^, ^20^, ^21^, ^22^ ^]^ Recently, CDs have been used as PCs to drive a number of carbon—carbon and carbon—heteroatom bond forming organic transformations, including the 1) oxidations of simple organic substrates, 2) fluroalkylation of electron‐rich organic compounds, 3) cross‐coupling reactions, and 4) aldol condensations, among others.^[^ ^20^, ^23^, ^24^, ^25^, ^26^, ^27^, ^28^ ^]^ Despite some successful applications in recent years, the use of CDs as photocatalysts for organic synthesis seems to be still in its infancy.^[^ ^29^ ^]^ An extremely intriguing application would be to couple the photocatalytic abilities of CDs with a non‐photochemical second catalytic route to exploit possible synergy effects on more challenging chemical conversions. Within this strategy, also known as “dual photocatalysis”, reactive intermediates (such as radicals) produced through a photoredox process may be involved in an organo‐ or transition‐metal catalytic cycle, leading to the formation of densely functionalized organic products.^[^ ^30^ ^]^ In this regard, dual photocatalytic [3 + 2] cycloaddition reactions offer robust, simple, and atom‐economical approaches for the construction of valuable five‐member ring compounds, which are widely found both in natural products and pharmaceuticals.^[^ ^31^, ^32^, ^33^, ^34^, ^35^, ^36^, ^37^, ^38^, ^39^ ^]^ One example of this type of reactivity was reported in 2016 by Yoon and co‐workers, who developed an asymmetric [3 + 2] photocycloaddition reaction between aryl cyclopropyl ketones and terminal olefins to prepare enantioenriched cyclopentane derivatives in good yields with high enantioselectivity (**Figure** **1**A).^[^ ^40^ ^]^ In particular, the authors used Ru(bpy)_3_(PF_6_)_2_ as the PC in combination with a chiral gadolinium‐based Lewis acid. Moreover, in 2023, the Waser group reported a novel photocatalytic protocol for the direct [3 + 2] cycloaddition of disubstituted cyclopropanes with alkynes and olefins, employing an iridium‐based photocatalytic system. In this case, the use of highly reactive 2,2‐dimethylcyclopropanes ensured an efficient homolytic fission of the C—C bond within the cyclopropane, yielding the corresponding five‐membered cycloadducts from moderate to good yields (Figure 1B).^[^ ^41^ ^]^

Nevertheless, it is important to notice that available photocatalytic strategies for the construction of five‐membered rings generally rely on the use of expensive and potentially harmful transition metal–based homogeneous catalytic systems. In this regard, it has recently emerged that these catalytic systems are not attracting industrial interest due to their limitations in terms of recovery and recycling.^[^ ^30^ ^]^

Here, we have tackled these challenges and reported the development of a novel metal‐free dual nano‐catalytic strategy to promote [3 + 2] cycloaddition reactions between aryl cyclopropyl ketones (**1**) and alkynes/alkenes (**2**), producing an excellent yield of five‐membered cycloadducts (**3**) with good diastereoselectivity, while avoiding the use of expensive and toxic metal complexes (Figure 1C). In particular, a new type of phenol‐rich CDs (*Ph*‐CDs) has been prepared, characterized, and used as a photocatalytic system in combination with a suitable base (namely DIPEA: *N*,*N*‐diisopropylethylamine) and an organocatalyst, such as the Schreiner thiourea (**A**). In this work, we aimed at precisely controlling the chemical nature and tailoring the reactivity of CDs’ photocatalytic sites by transferring the useful behavior of phenolate anions from a molecular level to the nanoscale. Interestingly, mechanistic investigations highlighted the unique role played by **A** in the studied transformation. In fact, we found that this organocatalyst may not only activate the carbonyl moiety of **1** but also stabilize the formation and enhance the photocatalytic performances of phenolate anions on the nanoparticle surfaces. In this manner, we did expand the functions of organocatalysis by describing an unprecedent dual‐reactivity profile of a well‐established molecular organocatalyst, such as **A**. This novel activation mode demonstrated how the effective merging of organo‐ and nano‐catalysis may unlock previously inaccessible reaction pathways, thus providing new intriguing opportunities for the sustainable production of relevant chemicals. In addition, *Ph*‐CDs proved to be much more active than molecular phenols in the studied reaction. Lastly, we demonstrate that *Ph*‐CDs could be easily recovered and reused up to three times without any significant drop in yield.

## 2 Results and Discussion

**Figure 2:**
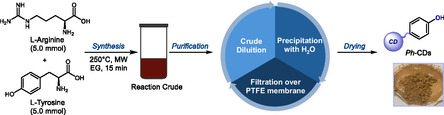
Synthesis and purification process of *Ph*‐CDs. PTFE: polytetrafluoroethylene; MW: microwave; EG: ethylene glycol.

Phenol‐rich CDs (*Ph*‐CDs) were synthesized following a simple, fast, and robust microwave (MW)‐assisted procedure (**Figure** **2**), using readily available and inexpensive amino acids as precursors. The MW‐assisted synthesis results in a high temperature and pressure, promoting the formation of the CDs while retaining some of the properties of the starting materials into the final nanoparticles. In particular, L‐tyrosine (Tyr) was selected to introduce phenol moieties on the resulting material and L‐arginine (Arg) as a bulk, highly reactive, carbon source. Previous studies regarding the formation mechanism of amorphous CDs produced from Arg highlighted that their core mainly originates from this amino acid.^[^ ^42^, ^43^ ^]^ Initially, milli‐Q water was chosen as the reaction medium. However, this strategy proved to be ineffective, possibly due to a limited heat transfer that resulted in the presence of unreacted starting materials within the reaction crude. To resolve this issue, ethylene glycol (EG) was chosen as the optimal solvent thanks to its convenient dielectric constant that allows higher conversion of electromagnetic energy into heat.^[^ ^44^ ^]^ Thus, in a typical experiment, 1 equivalent of Arg (874 mg, 5.0 mmol) and Tyr (906 mg, 5.0 mmol) were combined into an MW reaction vessel with EG (2.5 M, 2 mL) and thermally treated for 15 min at 250 °C. The crude oil obtained was diluted with *N*,*N*‐dimethylformamide (DMF) and the precipitation of *Ph*‐CDs was induced by dropwise addition of milli‐Q water. The obtained precipitate was then solubilized again in DMF and reprecipitated from water up to five times. A remarkably high yield of pure *Ph*‐CDs was obtained in the form of a brownish powder (20% yield based on weight, 350 mg). Based on several reports in literature, we assume that *Ph*‐CDs possess different polar chemical functionalities on their surfaces, including phenols, that could be left over from the polymerization of the molecular precursors.^[^ ^45^, ^46^, ^47^ ^]^

**Figure 3:**
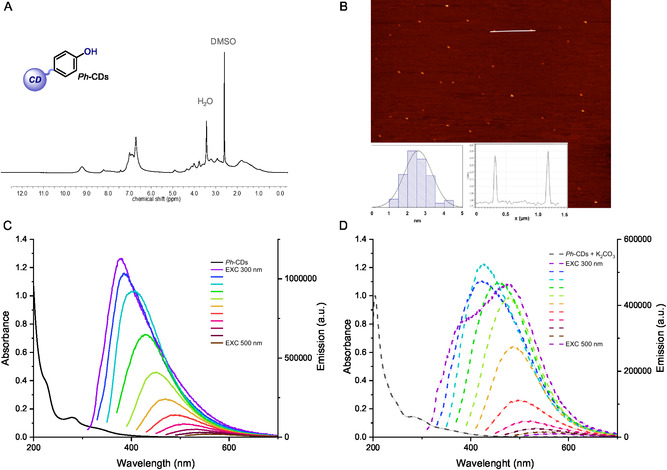
Characterization of *Ph*‐CDs. A) ^1^H‐NMR spectrum of *Ph*‐CDs (DMSO‐*d*_6_, 500 MHz). B) Tapping mode AFM of *Ph*‐CDs deposited on a mica substrate. C) Absorption and emission spectra of *Ph*‐CDs recorded at different excitation wavelengths. D) Absorption and emission spectra of deprotonated *Ph*‐CDs with K_2_CO_3_ (0.02 M) recorded at different excitation wavelengths. All UV–vis and fluorescence spectra were measured in MeCN/H_2_O (3:1) at 0.1 mg mL^−1^ concentration of *Ph*‐CDs. DMSO = dimethyl sulfoxide.

The purification of CDs is a critical step during synthesis and involves removing the starting materials and by‐products. However, this aspect is often overlooked in the pursuit of new CDs, leading to misinterpretations of the materials properties.^[^ ^48^, ^49^ ^]^ Consequently, the effective purification of the nanoparticles from the molecular side‐products was verified by nuclear magnetic resonance (NMR) analysis. In particular, sharp signals deriving from molecular species were absent from the ^1^H‐NMR spectrum of *Ph*‐CDs which instead showed broad signals typical of nanostructured materials (**Figure** **3**A).^[^ ^48^ ^]^ Additionally, the broad band in the aromatic region of the spectrum (in the range of 6.5–8.5 ppm) was a clear indication of the presence of aromatic moieties within the *Ph*‐CDs, such as phenol derivatives. As further proof of the purity of our nanoparticles, diffusion‐ordered spectroscopy (DOSY) analysis revealed that these carbon nanoparticles have a distinctive diffusion coefficient (*D* = 1.82×10^−6^ cm^2^ s^−1^) that is comparable to other values reported in the literature for similar nanomaterials (see Figure S5, Supporting Information).^[^ ^50^ ^]^ Structural and morphological information on the nanoparticles was gathered by thermogravimetric analysis (TGA), attenuated total reflectance‐Fourier‐transform infrared (IR) spectroscopy, elemental analysis, and atomic force microscopy (AFM). TGA attested to a predominantly amorphous structure of the *Ph*‐CDs with a relative thermal stability up to 300 °C and a subsequent loss of mass between 300 and 500 °C (see Figure S4, Supporting Information). The IR spectrum of the nanoparticles showed an intense broadband from 3650 to 3000 cm^−1^ that corresponds to —OH, —NH, and —CH stretches in aromatics, while the C=C stretch in aromatics occurs in the region between 1600 and 1500 cm^−1^ (see Figure S4b, Supporting Information). Elemental analysis of *Ph*‐CDs specified the listed atomic percentages, C: 63.52%, H: 6.19%, N: 10.27%, and O: 20.02%. A similar content of elements is found in Tyr, especially regarding the O%, thus suggesting that this amino acid contributes heavily to the formation of *Ph*‐CDs. Moreover, AFM analysis confirmed the nanoscale dimensions of *Ph*‐CDs, displaying an average size of 2.5 ± 0.7 nm (Figure 3B). Regarding the photochemical properties of *Ph*‐CDs, UV–vis spectroscopy showed a broad absorption band which extended into the visible region (albeit mildly), up to 450 nm (Figure 3C). The typical excitation wavelength‐dependent behavior of this class of nanomaterials has also been observed for *Ph*‐CDs (Figure 3C). Indeed, the value of the fluorescence peak shifts from 380 to 570 nm when the excitation wavelength is changed from 300 to 500 nm. Nevertheless, the fluorescence quantum yield of *Ph*‐CDs is independent of the excitation wavelength, assuming a value of 10.0% ± 0.5%. Interestingly, while recording the UV–visible spectrum and the emission profile of a solution of *Ph*‐CDs treated with K_2_CO_3_ (0.02 M in MeCN/H_2_O 3:1), a detectable bathochromic shift was recognizable in both cases (Figure 3D). It is well known that molecular phenolates show redshifted absorption and emission when compared to their protonated counterparts due to an increased conjugation within these aromatic anions.^[^ ^15^, ^51^ ^]^

**Figure 4:**
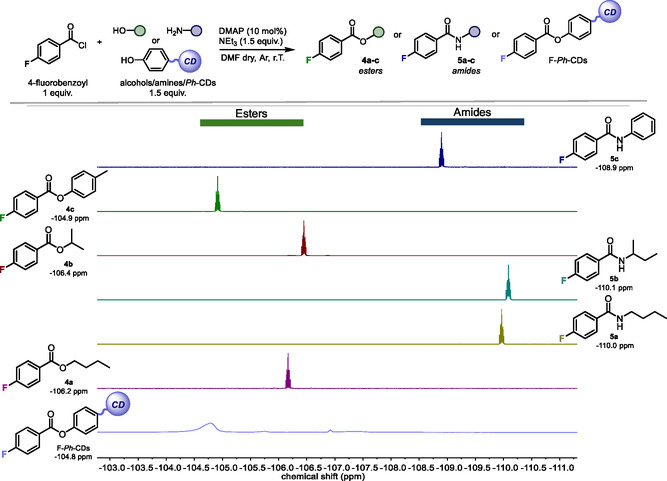
Fluorinated functionalization of alcohols/amines and *Ph*‐CDs and ^19^F‐NMR analysis of their corresponding products. Acylation of alcohols/amines/*Ph*‐CDs with 4‐fluorobenzoyl chloride (1 equiv., 0.5 mmol) were performed in DMF (0.05 M). Stacked ^19^F‐NMR spectra of their corresponding esters **4a**–**c**, amides **5a**–**c** and F‐*Ph‐*CDs were recorded in DMSO‐*d*_6_. DMAP = 4‐(dimethylamino)pyridine.

Consequently, these observations provided further proof of the presence of phenol groups on the *Ph*‐CDs’ surfaces. The fluorescence lifetimes of *Ph*‐CDs were also measured resulting in a double‐exponential fit with a short τ_1_ = 2.8 ns and a longer τ_2_ = 6.8 ns component (see Figure S3, Supporting Information). Subsequently, ^19^F‐NMR analysis was employed to detect and quantify the phenol moieties present on the surfaces of *Ph*‐CDs (**Figure** **4**). Our group has already explored this technique as a valuable tool not only for studying the nature of surface functionalities on CDs but also for quantifying them, thanks to the high sensitivity of the fluorine nucleus.^[^ ^52^, ^53^ ^]^ Here, we employed a post‐synthetic modification strategy to covalently attach a suitable fluorinated probe to the surface moieties of *Ph*‐CDs. Considering the complex chemical surfaces of *Ph*‐CDs, the functionalization of the superficial polar groups would form different fluorinated species. In theory, their ^19^F‐NMR signals should be distinguished because this technique is able to detect the formation of different fluorinated groups accurately. To verify this assumption, a series of molecular alcohols and amines were reacted with 4‐fluorobenzoyl chloride in the presence of 4‐(dimethylamino)pyridine (DMAP) and triethylamine (NEt_3_). The acylated products, namely esters and amides, were then analyzed using ^19^F‐NMR in dimethyl sulfoxide (DMSO)‐*d*_6_. Figure 4 shows the stacked spectra of the model products **4a**–**c** and **5a**–**c**. Interestingly, the chemical shifts of the amides **5a**–**c** (from −108.9 to −110.1 ppm) could be differentiated from those of the esters **4a**–**c** (from −104.9 to 106.2 ppm).

**Figure 5:**
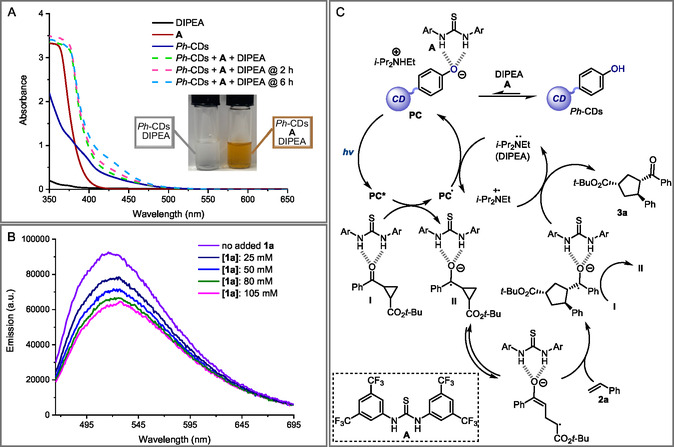
Mechanistic investigation of photocatalyzed [3 + 2] cycloaddition using *Ph*‐CDs. A) Optical absorption spectra recorded in MeCN: [DIPEA] = 100 mM; [**A**] = 10 mM; and [*Ph*‐CDs] = 50 mM. The dashed lines correspond to the mixture of *Ph*‐CDs + **A** + DIPEA (green line after 0 h, pink line after 2 h, and light blue line after 6 h). B) Quenching of the catalytic system (*Ph*‐CDs + **A** + DIPEA) emission ([*Ph*‐CDs + **A** + DIPEA] = 0.015 M in MeCN, excitation at 456 nm) in the presence of increasing amounts of **1a**. C) Mechanism of the photocatalytic [3 + 2] cycloaddition process.

In addition, aliphatic amides and esters could also be distinguished from the aromatics by their chemical shifts.^[^ ^54^ ^]^ Consequently, we performed the acylation reaction of the surface groups of *Ph*‐CDs to produce fluorinated nanoparticles (F‐*Ph*‐CDs). It is worth mentioning that the ^19^F‐NMR spectrum of the purified material showed a broad signal in the ^19^F‐NMR spectrum centered around −104.8 ppm, matching the signal of the model product obtained from *p*‐cresol (namely **4c**). Therefore, this easy and precise examination established the presence of available phenol groups on the *Ph*‐CDs surfaces. Moreover, the number of superficial phenol moieties was measured at 2.30 ± 0.2 mmol g^−1^ using α,α,α‐trifluorotoluene as an internal standard. To take advantage of the photochemical properties of *Ph*‐CDs, we tested their feasibility as PCs. In particular, we first focused our attention on the [3 + 2] photocycloaddition reaction between cyclopropyl ketones **1** and olefins **2**. To exploit the unique photoredox characteristics of phenolates in their excited state, the phenol moieties on *Ph*‐CDs should be deprotonated. To this end, it is evident that a base had to be added to the reaction mixture. A reductive quencher to restore the photocatalyst and a Lewis acid to activate the cyclopropyl ketone also appeared necessary (see **Figure** **5**C for details). For these reasons, cyclopropane **1a** was selected to react with styrene **2a** in the presence of *Ph*‐CDs (13 mol% of phenol groups, based on NMR analysis), Gd(OTf)_3_ (20 mol%), and DIPEA (*i*‐Pr_2_NEt, 2 equiv.). This preliminary experiment was conducted at room temperature in acetonitrile and under irradiation by a Kessil lamp at 456 nm (lamp power: 50 W). Under these conditions, the corresponding cyclopentane **3a** was isolated in 94% of yield as a diastereomeric mixture (**Table** **1**, entry 1). This promising result prompted us to pursue a more sustainable metal‐free strategy avoiding the use of lanthanide Lewis acid, which is an expensive and toxic reagent.

**Table 1:** Optimized reaction conditions and control experiments.

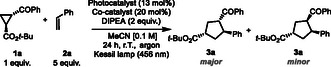				
Entry	Deviation from the standard conditions	Photocatalyst	Co‐catalyst	Yield% (d.r.)a)
1	–	*Ph*‐CDs	Gd(OTf)_3_	94 (4:1)
2	–	*Ph*‐CDs	B(C_6_F_5_)_3_	0
3	–	*Ph*‐CDs	SiMe_3_Cl	90 (3:1)
4	–	*Ph*‐CDs	**A**	98 (3:1)
5	In the dark at room temperature or at 50 °C	*Ph*‐CDs	**A**	0
6	In air	*Ph*‐CDs	**A**	0
7b)	–	*Ph*‐CDs	**A**	0
8	–	–	**A**	0
9	–	*Ph*‐CDs	–	12
10	–	L‐tyrosinec)	**A**	46 (3:1)
11	–	Phenolc)	**A**	25 (3:1)
12	–	*NH*_*2*_‐CDs	**A**	0

Reactions were performed on a 0.1 mmol scale. **A**: Schreiner thiourea; *Ph*‐CDs: phenol‐carbon dots.

a) Yield determined by ^1^H‐NMR using 1,3,5‐trimethoxybenzene as an internal standard; d.r. was determined from reaction crude by ^1^H‐NMR.

b) Without DIPEA or adding TEMPO (5 equiv.).

c) Catalytic loading: 20 mol%.

Consequently, we screened various metal‐free co‐catalysts for the activation of the cyclopropyl ketone **1**. The formation of the final product was not observed when the reaction was performed in the presence of B(C_6_F_6_)_3_ (Table 1, entry 2). From entry 3, a 90% yield of **3a** was resulted from using SiMe_3_Cl as the co‐catalytic system. Remarkably, a 98% yield of **3a** was obtained with a good diastereomeric ratio by employing the Schreiner thiourea (**A**). This seemed to be the best option for a metal‐free variant of the studied reactivity (Table 1, entry 4). Additional screening experiments are detailed in Supporting Information (see Table S1, Supporting Information). Control experiments showed that no product formation was observed in the absence of light, both at ambient temperature and at 50 °C, indicating the photochemical nature of the process (Table 1, entry 5). In this regard, we also observed that this chemical process was quenched when turning off the light during the catalytic experiment. Additionally, the inhibition of the reaction under aerobic conditions supported a radical mechanism (Table 1, entry 6). This was further corroborated by the experiment conducted in the presence of 2,2,6,6‐tetramethylpiperidine 1‐oxyl (TEMPO, 5 equiv.), since the formation of **3a** was not detected (Table 1, entry 7). The exclusion of any of the reaction components, namely *Ph*‐CDs, **A**, and DIPEA, completely suppressed the process (Table 1, entries 7–9). Moreover, performing the model reaction using 20 mol% of molecular photocatalysts, such as Tyr (Table 1, entry 10) or phenol (Table 1, entry 11), provided the desired product in low yields (up to 46%). Importantly, *Ph*‐CDs proved to be much more active than molecular phenols in the model reaction. Thus, these results suggest that the photochemical properties of the nano‐catalysts (*Ph*‐CDs) are essential to achieve high productivity in the studied transformation. Lastly, the use of amine‐rich CDs (*NH*_*2*_‐CDs), produced from Arg and 1,2‐diaminoethane, as photocatalysts did not provide the desired product (Table 1, entry 12).^[^ ^52^ ^]^ In particular, in this experiment, the starting substrates were completely recovered. This further confirmed the importance of having reactive and accessible phenols on the nanoparticles’ surfaces.

UV–vis spectroscopy studies help us to elucidate the mechanism of this photocatalytic reaction. In the reaction mixture, DIPEA (2 equiv.) might play a dual role as this tertiary amine can act 1) as a base to deprotonate the phenol moieties on *Ph*‐CDs and/or 2) as an electron donor to close the photocatalytic cycle. Specifically, UV–vis studies were utilized to evaluate the level of deprotonation of the surface groups on *Ph*‐CDs upon addition of DIPEA. In particular, we observed that the addition of DIPEA to a solution of *Ph*‐CDs did not show any appreciable change of the absorption spectrum, thus excluding an effective deprotonation of the nanoparticles under such conditions. Indeed, according to the *pK*_a_ values of molecular phenols and DIPEA in acetonitrile,^[^ ^55^, ^56^ ^]^ the deprotonation of these surface aromatic groups should be minimal. Interestingly, during additional UV–vis analysis, we noticed that the solution obtained by mixing *Ph*‐CDs, **A**, and DIPEA developed a pale yellow color after 2 h (Figure 5A). Consequently, the optical absorption spectrum of this mixture showed a strong bathochromic shift toward the visible spectral region (pink dashed line, Figure 5A). Remarkably, the appearance of the yellow color became even more prominent overtime (light blue dashed line, Figure 5A), suggesting the relatively slow formation of chemical species capable of absorbing visible light in solution. The absorption spectrum of a freshly prepared solution of *Ph*‐CDs, **A**, and DIPEA displayed a moderate bathochromic shift, possibly implying that in this case the new chromophore was partly formed (green dashed line, Figure 5A). In contrast, a solution prepared by mixing *Ph*‐CDs and DIPEA did not develop any color and was found to be stable overtime. Therefore, we envisioned that the combination of these three components (*Ph*‐CDs, **A**, and DIPEA) could result in the formation of colored complexes, capable of photochemically initiating the cycloaddition reaction. Concerning this, thioureas have been reported and described as effective chelating agents for spherical anions via hydrogen‐bonded interactions.^[^ ^57^ ^]^ Moreover, the presence of electron‐withdrawing groups (namely —CF_3_) within **A** makes the protons of the thioamide moiety highly acidic, thus improving their anion binding affinity and enhancing the thiourea's ability to disperse the negative charge of the bound anion.^[^ ^58^ ^]^ It is also important to point out that the complexation of a thiourea with an anion is typically characterized by the formation of a new absorption band that typically reaches the visible region of the spectrum.^[^ ^59^ ^]^ Hence, we believe that **A**, when in the presence of DIPEA, could slowly shift the deprotonation equilibrium of the phenol groups on *Ph*‐CDs by coordinating and stabilizing their conjugate bases, namely phenolate anions. Interestingly, these charge‐transfer surface anionic aggregates, which are held together by non‐covalent interaction, showed an improved visible‐light absorption capacity.

This is actually extremely important, because this coordination allowed for a more effective photoexcitation of the catalytic systems (Figure 5A). Conversely, both *Ph*‐CDs and their deprotonated form exhibited a limited absorptivity at 456 nm (Figure 3A–D). Other phenomena, such as the formation of EDA complexes between the reaction components, were excluded through additional absorption studies (see Figure S8, Supporting Information). To confirm the presence of phenol‐based moieties on the *Ph*‐CDs, which can generate a visible‐light absorbing system with **A** and DIPEA, we recorded the absorption spectrum of a molecular phenol under the same conditions. Thus, a solution containing 4‐*tert*‐butylphenol, **A**, and DIPEA was prepared and analyzed by UV–vis spectroscopy. Even in this case, a prominent, redshifted absorption of the solution was observed overtime (see Figure S9, Supporting Information). This test confirmed the photochemical behavior of phenol groups when in the presence of **A** and DIPEA, while also supporting the presence of phenol groups on *Ph*‐CDs. The model reaction was also performed in the absence of **A** and using strong bases instead of DIPEA to completely deprotonate the acidic sites on *Ph*‐CDs. Specifically, the use of 2 equiv. of either NaO*t*‐Bu or Cs_2_CO_3_ resulted in the formation of only traces of product **3a**. These experiments further suggested the key role played by the thiourea‐based organocatalyst, that is needed to activate the carbonyl moiety within **1**, while also highlighting the unique activation mode of phenols which can be obtained by employing **A** and DIPEA. We then examined the reaction kinetics under optimized conditions (Table 1, entry 4), performing the transformation at different reaction times, spanning from 1 to 24 h. In particular, we observed that, within the first 2 h, the starting materials were not consumed. After this induction period, the yield of **3a** began to grow, becoming quantitative after 8 h (see Figure S13, Supporting Information). Based on these results and on the UV–vis spectroscopic studies, we supposed that the formation of a certain number of anionic surface aggregates, that are capable of effectively absorbing light at 456 nm and thus photochemically initiating the cycloaddition reaction, required at least 2 h. To further validate this claim, we monitored the progress of the model reaction between **1a** and **2a** as follows. First 1) *Ph*‐CDs, **A**, and DIPEA were mixed in acetonitrile for 2 h to assess the formation of the photocatalytic system and then 2) the reactants (**1a** and **2a**) were added to this solution which was stirred for additional 6 h under light irradiation. This experiment almost provided a quantitative yield of product **3a**, therefore corroborating our hypothesis (see Figure S6, Supporting Information). Stern–Volmer quenching experiments were performed to better understand the initiation mechanism of this photocatalytic reaction (Figure 5B). Thus, the emission spectrum of the colored adduct, formed by mixing *Ph*‐CDs, **A**, and DIPEA, was recorded after excitation at 456 nm. A progressive quenching of its emission intensity was observed upon increasing the concentration of cyclopropane **1a**. In particular, the quenching of emission can occur through a variety of processes. However, the data incorporated in the Stern–Volmer equation revealed a linear correlation in the range 25–105 mM of the quencher (*K*_sv_ = 4.66 M^−1^).

This is indicative of the occurrence of a single type of quenching mechanism, likely via a single‐electron transfer (SET) process. With this data in mind, we proposed a reaction mechanism (Figure 5C) that starts from the crucial deprotonating equilibrium of phenols on *Ph*‐CDs. In fact, in the presence of DIPEA, this equilibrium is completely shifted toward its neutral form. In contrast, thanks to the coordination between **A** and surface phenolate anions, the equilibrium may slowly shift toward the anionic form of *Ph*‐CDs. In this way, the actual photocatalytic system (PC) can be produced in solution. When subjected to 456 nm light irradiation, this PC reaches its electronically excited state (PC*) and becomes a strong reducing agent. Then, an SET occurs from the PC* to the cyclopropane **1a**, that is presumably activated by **A**. The reduced intermediate **II** then fragments giving its corresponding ring‐opening form. Subsequently, this intermediate reacts with **2a** to afford a ketyl radical. Lastly, the oxidation of this radical anion leads to the formation of product **3a**. Specifically, either **I** or the radical cation of DIPEA could serve as oxidants in this redox step. Unfortunately, the idea of developing an enantioselective variant of the studied photochemical transformation, employing chiral and enantiopure thioureas as co‐catalytic systems, turned out to be unfeasible (*ee%* up to 7%, see Table S2, Supporting Information).

**Scheme 1:**
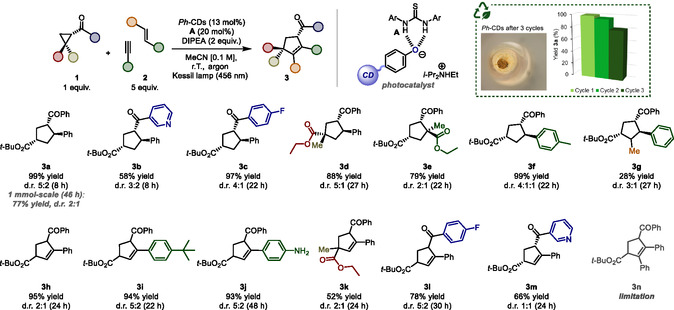
Evaluation of the scope of cyclopropanes **1** and alkenes or alkynes **2** for the [3 + 2] cycloaddition reaction. Reactions were performed on a 0.1 mmol scale using *Ph*‐CDs, Schreiner thiourea (**A**), DIPEA. The reaction mixtures were illuminated with a Kessil lamp at 456 nm for 8–48 h. Yields reported are the combined isolated yields of all diastereomers; d.r. was determined from crude NMR. Major diastereomer are shown for **3a**–**g**. Recyclability tests on *Ph*‐CDs as photocatalyst were performed for three catalytic cycles. Yields of **3a** in the graph were determined by ^1^H‐NMR analyses, using 1,3,5‐trimethoxybenzene as internal standard. See Supporting Information for additional experimental details.

To demonstrate the generality of our approach, we evaluated the synthetic potential of this photocatalytic reaction by allowing different cyclopropanes to react with unsaturated hydrocarbons (**Scheme** **1**). In particular, a cyclopropane bearing a heteroaromatic group (**1b**) was well tolerated under the reaction conditions, producing a good yield of the corresponding product **3b** with a moderate diastereoisomeric ratio (58% yield and d.r.: 3:2). Moreover, the use of **1c**, which contains an electron withdrawing group on the aryl fragment, resulted in a high yield of the desired product **3c** (97%) with good diastereoselectivity (d.r.: 4:1). Interestingly, both cyclopropane and olefin containing 1,1‐disubstituents were also suitable substrates for this transformation. As a matter of fact, these substrates afforded the corresponding cyclic products **3d**–**e** in high overall yields as mixture of two diastereoisomers (d.r.: up to 5:1). In addition, the use of a styrene decorated with an alkyl group afforded compound **3f** in excellent yield along with a good diastereoisomeric ratio. Importantly, our methodology also functionalized an internal olefin resulting in a moderate yield of the product **3g** as two diastereoisomers. To demonstrate the capability of our photocatalytic system to produce substituted cyclopentenes, different alkynes (**2h**–**n**) were tested as substrates. In this regard, phenylacetylene gave good results both in terms of diastereoselectivity and yield (product **3h**: 95% yield, d.r.: 2:1). Remarkably, alkynes bearing either a free primary amine or steric‐hindered substituents reacted effectively under the optimized conditions, leading to very good overall yields of the desired cyclopentenes **3i** and **3j** (d.r.: up to 5:2). In addition, the use of a cyclopropane containing a quaternary carbon provided a moderate yield of the adduct **3k** with a poor diastereoselectivity. Lastly, electron‐deficient cyclopropyl ketones effectively reacted with the ethynylbenzene leading to very good overall yields of the desired unsaturated rings **3l**‐**3m**. Unfortunately, nonterminal alkynes did not participate in the reaction process (product **3n**). In this regard, we believe that the poor reactivity of this class of alkynes is due to their increased steric hindrance. In fact, this aspect may prevent them to effectively react with the ring‐opening intermediate, which is obtained upon fragmentation of **II** (see Figure 5c). Interestingly, the photocatalytic process is amenable to scaleup (1 mmol, product **3a**) with a poor erosion of the chemical yield (77% yield). Lastly, we also demonstrated that *Ph*‐CDs could be recycled up to three times (green box in Scheme 1). Remarkably, the recyclability tests provided comparable performances in terms of reaction yield and diastereoselectivity (see Supporting Information for details). The partial loss of activity observed over the third cycle could be attributed to both mass loss during *Ph*‐CDs recovery and partial degradation of the nanoparticles. As reported in Figure S12, Supporting Information, the ^1^H‐NMR, UV–vis, and fluorescence spectra of *Ph*‐CDs after catalysis showed some differences compared to that of the pristine ones. These differences were ascribed to small changes in the chemical structure of the CDs due to photobleaching and to possible irreversible radical side reactions occurred during catalysis.

## 3 Conclusion

The intriguing photophysical features of CDs inspired us to design a novel *bottom‐up* synthetic approach that allows the production of novel phenol‐rich nanoparticles. The use of a wide set of characterization techniques provided strong evidence for the formation of quasi‐spherical nanometric particles bearing a high number of superficial phenol groups. In particular, the presence of phenolic moieties on the *Ph*‐CDs was demonstrated by ^19^F‐NMR spectroscopy. Moreover, a novel photocatalytic activation mode for CDs was developed. Specifically, we demonstrated that the phenol moieties present on the nanoparticles’ surfaces can generate, when mixed with **A** and DIPEA, colored anionic non‐covalent aggregates. These photoactive intermediates may be used as visible‐light photocatalytic systems to drive [3 + 2] photocycloaddition reactions between cyclopropane **1** and unsaturated compounds **2**. Interestingly, this photocatalytic protocol provided a large number of relevant five‐membered rings **3**. The isolated yield of these products ranged from moderate to excellent along with good levels of diastereoselectivity. Importantly, the phenol‐rich nanoparticles exhibited excellent stability under the reaction conditions and could be recycled for at least three independent cycles. In conclusion, this article has highlighted the potential of suitably engineered CDs as green and metal‐free photocatalysts to drive appealing organic reactivities under mild operative conditions, while replacing toxic or expensive traditional PCs.

## Supporting Information

Supporting Information contains experimental procedures, including the synthesis of starting materials and characterization data, and NMR spectral data for all new compounds.

## Conflict of Interest

The authors declare no conflict of interest.

## Supporting information

Supplementary Material

## Data Availability

The data that support the findings of this study are available in Supporting Information of this article.
