# Supplementary material for: Phenol‐Rich Carbon Dots as Metal‐Free Nano‐Photocatalysts for [3 + 2] Cycloaddition Reactions
Source: ChemSusChem. 2025 Apr 17;18(13):e202500521. doi: 10.1002/cssc.202500521 (PMC12232118; doi:10.1002/cssc.202500521)
Supplement: Supplementary file 1 — Supplementary Material [file CSSC-18-e202500521-s001.pdf]

Supporting Information for

Phenol-Rich Carbon Dots as Metal-Free Nano-Photocatalysts for [3+2] Cycloaddition Reactions

Martina Mamone,[1‡] Giuseppe Gentile,[1‡] Maurizio Prato,[1,2,3]* and Giacomo Filippini[1]*

[1] Department of Chemical and Pharmaceutical Sciences, INSTM UdR Trieste, University of Trieste, Trieste 34127, Italy

[2]Centre for Cooperative Research in Biomaterials (CIC biomaGUNE), Basque Research and Technology Alliance (BRTA), Donostia San Sebastián, 20014, Spain

[3]Ikerbasque, Basque Foundation for Science, Bilbao 48013, Spain

‡These authors contributed equally to this work

Table of Contents

1.General Information and Synthetic Procedures 2

1.1 Synthesis of *Ph*-CDs 2

1.2 Preparation of Cyclopropanes 1a-d 2

1.3 Procedure for the Preparation of Reference Compounds for the Detection of Phenol Moieties 4

1.4 Procedure for the Detection of Phenol Moieties on F-*Ph*-CDs Surface 5

2. Characterization of *Ph*-CDs 6

3. General Procedure for the Catalytic Experiments 7

3.1 General Procedure for the Photocatalytic [3+2] Cycloaddition Reaction 7

3.2 Screening of the Conditions of the [3+2] Cycloaddition 13

3.3 Monitoring of the Catalytic Adduct 14

3.4 Stern-Volmer Quenching Study 14

4. UV-Vis Experiments 15

4.1 Absorption Spectra 15

5. Recycling Experiments 16

6. Kinetic Study of the [3+2] Cycloaddition 18

7. NMR Spectra 19

7.1 ^{1}H -^{13}C-^{19}F NMR 19

7.2 Bidimensional Spectra 3a (minor diastereoisomer) 56

8. References 56

1.General Information and Synthetic Procedures

General Information: The microwave synthesis was performed on a CEM Discover-SP instrument. UV-Vis measurements were carried out on Cary 5000 UV-Vis-NIR. All the spectra were recorded at room temperature using 10 mm path-length quartz cuvettes. Absorption spectra of compounds were recorded with an Agilent Cary 5000 UV-Vis spectrophotometer. Emission measurements were performed on an Edinburgh instruments FS5 spectrofluorometer using a 150 W CW Ozone-free xenon arc lamp as source and a Photomultiplier R928P (spectral coverage 200 nm – 900 nm, cooled and stabilised) as detector. Quantum yields were performed using the integrating sphere setup SC-30. Luminescence lifetimes were measured with an Edinburgh Instruments FS5 time-correlated single-photon counting spectrofluorimeter, exciting the sample at 375 nm with a picosecond pulsed diode laser (EPL-375 Edimburgh Instruments). TGA was performed with a TGA Q500 (TA instruments), under a flow of N_2 (25 mL/min), following a temperature program consisting of the equilibration of the sample at 100°C for 10 minutes followed by a ramp at 5°C/min up to 800°C. The sample aliquot ranged from 1 to 2 mg, exactly weighed. ATR-IR measurements were performed using a Spectrum 2000 FT-IR Instrument (Perkin Elmer). The NMR spectra were recorded on Varian 400 spectrometer (^{1}H: 400 MHz; ^{19}F-NMR: 376.0 MHz ^{13}C: 101.0 MHz). Chromatographic purification of products was accomplished using flash chromatography on silica gel (35-70 mesh). For thin layer chromatography (TLC) analysis throughout this work, Merck pre-coated TLC plates (silica gel 60 GF254, 0.25 mm) were employed, using UV light as the visualizing agent (254 nm), basic aqueous potassium permanganate ($KMnO_4$) stain solution or iodine, and heat as developing agents. Organic solutions were concentrated under reduced pressure on a Büchi rotatory evaporator. Commercial reagents and solvents were purchased from Sigma-Aldrich, Fluka, Alfa Aesar, Fluorochem and VWR. They were used as received, without further purification unless otherwise stated. Synthesis grade and anhydrous solvents were used as purchased.

1.1 Synthesis of *Ph*-CDs

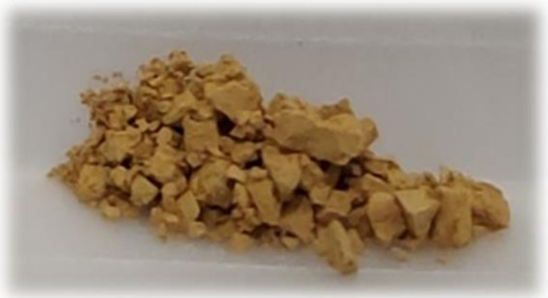

L-arginine (874.5 mg, 5 mmol) and L-tyrosine (905.9 mg, 5 mmol) were introduced in a sealable microwave vessel followed by 2 mL of ethylene glycol (2.5 M). The vessel was closed and then heated at 250°C (300 W) for 15 minutes. In the process of microwave heating, the solution changes color from a white suspension to a brown oil because of the formation of *Ph*-CDs. The solution was then diluted with *N*,*N*-dimethylformamide (DMF) ca. 3 mL, put under sonication and precipitation was induced by a dropwise addition of water up to 450 mL. The precipitate was collected though filtration over a polytetrafluoroethylene (PTFE) membrane with a 0.1 nm pore size. The precipitate was collected and dissolved in the minimal quantity of DMF, precipitation was re-induced under sonication using water. The precipitation process was repeated 4 times. The final powder was rinsed with diethyl ether (Et_2O) and left to dry under the fume hood. After drying, the final material is obtained as a brownish powder. The mass yield of the process was approximately 20% (350mg).

1.2 Preparation of Cyclopropanes 1a-d

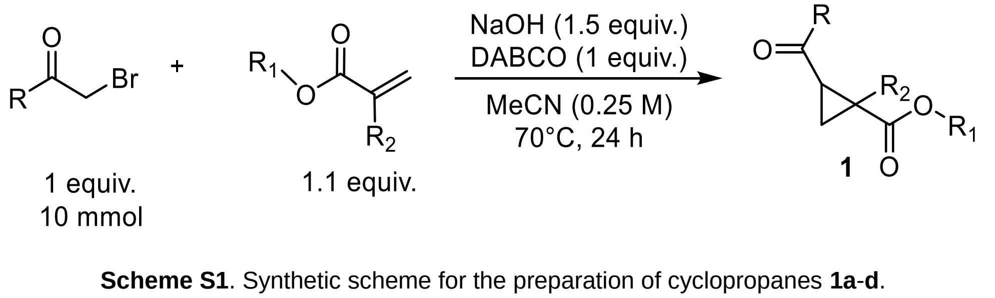

Scheme S1. Synthetic scheme for the preparation of cyclopropanes **1a**-**d**.

Dry MeCN (17 mL) was added to a round flask with 1,4-diazabicyclo[2.2.2]octane (DABCO; 10.0 mmol, 1.12 g) and the α-haloketone (10.0 mmol, 1 equiv.). A thick white precipitate formed immediately, and the mixture was allowed to stir at room temperature under a nitrogen atmosphere for 30 min. After this time, NaOH (0.6 g, 15.0 mmol, 1.5 equiv.) and the alkene (11 mmol, 1.1 equiv.) were added. The reaction flask was equipped with a reflux condenser and the reaction mixture was brought to 80°C until completion, as indicated by TLC. The reaction was then quenched with saturated aqueous ammonium chloride and extracted three times with Et_2O. The combined organic layers were washed once with brine, then dried over anhydrous Na2SO4, and concentrated under reduced pressure to give the crude product as a dark oil. The residue was purified by flash chromatography (CyHex/EtOAc) to afford the corresponding cyclopropanes **1a**-**d**.

<u>Characterization Data 1a-d</u>

***tert*-Butyl 2-benzoylcyclopropanecarboxylate (1a)**

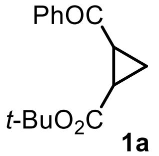

1a

1a was synthesized according to the general procedure from 2-bromo-1-phenylethanone (1.99 g, 10 mmol) and *tert*-butyl acrylate (1.34 mL, 11 mmol). The cyclopropane **1a** was obtained as solid (1.50 g, 60% yield).

^{1}H-NMR (400 MHz, $CDCl_3$) 8.02 (2H, d, *J*=7.0 Hz), 7.62 – 7.55 (1H, m), 7.49 (2H, t, *J*=7.7 Hz), 3.12 (1H, ddd, *J*=9.1, 5.7, 3.8 Hz), 2.30 (1H, ddd, *J*=8.6, 5.9, 3.8 Hz), 1.55 (2H, dddd, *J*=14.8, 9.1, 5.8, 3.3 Hz), 1.47 (9H, s). **^{13}C-NMR (101 MHz, $CDCl_3$)** δ 197.51, 171.56, 137.31, 133.42, 128.79, 128.41, 81.42, 28.23, 25.94, 25.92, 17.95. The characterization of the compound matches with the data reported in the literature.[1]

trans *tert*-Butyl 2-nicotinoylcyclopropanecarboxylate (1b)

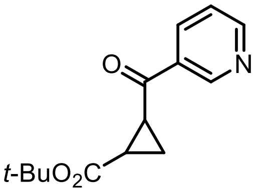

1b

1b was synthesized according to the general procedure from 3-(2-bromoacetyl)pyridin-1-ium bromide (2.8 g, 10 mmol) and *tert*-butyl acrylate (1.34 mL, 11 mmol). The cyclopropane **1b** was obtained as solid (1.6 g, 65% yield).

^{1}H-NMR (499 MHz, $CDCl_3$) δ 9.25 (d, *J* = 1.6 Hz, 1H), 8.81 (dd, *J* = 4.8, 1.7 Hz, 1H), 8.26 (dt, *J* = 8.0, 1.9 Hz, 1H), 7.44 (ddd, *J* = 8.0, 4.9, 0.9 Hz, 1H), 3.09 (ddd, *J* = 8.6, 5.7, 3.8 Hz, 1H), 2.34 (ddd, *J* = 8.8, 6.0, 3.8 Hz, 1H), 1.68 – 1.54 (m, 2H), 1.47 (s, 9H).**^{13}C-NMR (126 MHz, $CDCl_3$)** δ 196.32, 170.95, 153.67, 149.73, 135.48, 132.38, 123.60, 81.58, 28.06, 26.14, 25.90, 18.03. The characterization of the compound matches with the data reported in the literature.[1]

***tert*-Butyl 2-(4-fluoro)benzoylcyclopropanecarboxylate (1c)**

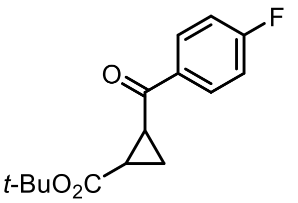

1c

1c was synthesized according to the general procedure from 2-Chloro-4'-fluoroacetophenone (1.7 g, 10 mmol) and *tert*-butyl acrylate (1.34 mL, 11 mmol). The cyclopropane **1c** was obtained as solid (1.8 g, 69% yield).

^{1}H-NMR (400 MHz, $CDCl_3$) δ 8.05 (dd, *J* = 9.0, 5.4 Hz, 2H), 7.16 (dd, *J* = 9.0, 8.4 Hz, 2H), 3.06 (ddd, *J* = 8.6, 5.7, 3.8 Hz, 1H), 2.30 (ddd, *J* = 8.6, 6.0, 3.8 Hz, 1H), 1.60 – 1.50 (m, 2H), 1.47 (s, 9H). **^{13}C-NMR (101 MHz, $CDCl_3$)** δ 195.91, 171.47, 166.06 (d, *J* = 255.3 Hz), 133.74 (d, *J* = 3.0 Hz), 131.06 (d, *J* = 9.2 Hz), 115.92 (d, *J* = 22.0 Hz), 81.52, 28.23, 25.92, 25.77, 18.00. **^{19}F-NMR (376 MHz, $CDCl_3$)** δ -104.92 (1F). **HRMS** calculated for $C_{15}H_{17}FO_3$ (M-Na): 287.1054 found: 287.1053.

ethyl 2-benzoyl-1-methylcyclopropane-1-carboxylate (1d) was synthesized according to the general procedure from 2-bromo-1-phenylethanone (1.99 g, 10 mmol) and ethyl methacrylate (1.37 mL, 11 mmol). The cyclopropane **1d** was obtained as solid (430 mg, 38% yield).

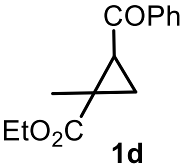

1d

^{1}H-NMR (499 MHz, $CDCl_3$) δ 7.94 – 7.89 (m, 2H), 7.56 – 7.47 (m, 1H), 7.44 – 7.37 (m, 2H), 4.18 (q, *J* = 7.1 Hz, 2H), 3.25 (dd, *J* = 8.3, 6.5 Hz, 1H), 1.62 (dd, *J* = 6.6, 3.9 Hz, 1H), 1.58 (dd, *J* = 8.2, 4.0 Hz, 1H), 1.26 (t, *J* = 7.1 Hz, 3H), 1.23 (s, 3H). **^{13}C-NMR (126 MHz, $CDCl_3$)** δ 195.88, 173.64, 137.98, 133.11, 128.61, 128.18, 61.31, 31.84, 29.57, 20.49, 14.20, 12.71. **HRMS** calculated for $C_{14}H_{16}O_3$ (M-Na): 255.1002 found: 255.1003.

1.3 Procedure for the Preparation of Reference Compounds for the Detection of Phenol Moieties

To synthesize the model compounds for ^{19}F-NMR experiments: 1.5 equiv. (0.75 mmol) of the nucleophile (alcohols or amines) were introduced in a flame-dried Schlenk tube followed by dry DMF (10 mL), 4-(Dimethylamino)pyridine (DMAP; 10 mol%) and triethylamine (0.75 mmol, 76.42 mg, 105 µL). The reaction was placed under argon atmosphere and heated at 40°C. The acylating reagent 4-fluorobenzoyl chloride (0.5 mmol, 79 mg, 60 mL, 1 equiv.) was solubilized in 2 mL of dry DMF and added dropwise. The solution turned immediately to a deep yellow color. The mixture was left to react overnight. The day after, it was quenched by the addition of 0.1 M HCl and extracted three times with ethyl acetate. The combined organic phases were reunited and dried over Na_2SO_4. The organic phase was concentrated under reduced pressure to give the crude product. The residue was purified by flash column chromatography (ethyl acetate/cyclohexane) to afford the desired products.

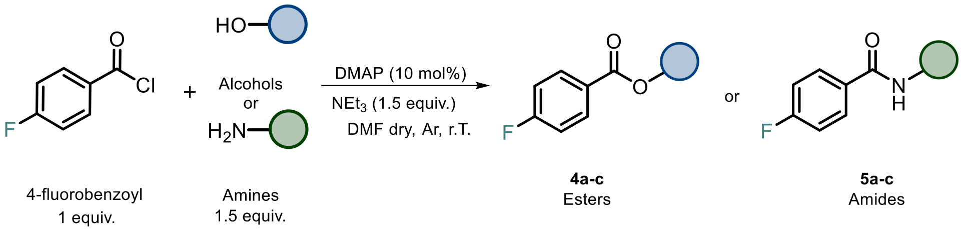

Scheme S2. Reaction scheme for the preparation of model compounds for ^{19}F-NMR tagging experiments.

Characterization Data 4a-c and 5a-c

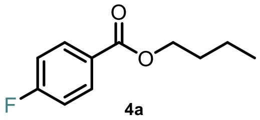

4a

Butyl-4-fluorobenzoate (4a)
Prepared according to the above-described procedure using 0.75 mmol (52.5 mg, 65 µL) of 1-butanol.
^{1}H-NMR (400 MHz, DMSO-d_6) δ 8.20 – 7.78 (m, 2H), 7.50 – 7.19 (m, 2H), 4.25 (t, J = 6.5 Hz, 2H), 1.79 – 1.56 (m, 2H), 1.54 – 1.32 (m, 2H), 0.91 (t, J = 7.4 Hz, 3H). ^{19}F-NMR (376 MHz, DMSO-d_6) δ -106.2(m).

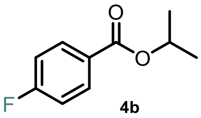

4b

Isopropyl-4-fluorobenzoate (4b)
Prepared according to the foregoing described procedure using 0.75 mmol (45.0 mg, 58 µL) of 2-propanol.
^{1}H-NMR (400 MHz, DMSO-d_6) δ 8.19 – 7.78 (m, 2H), 7.28 (m, 2H), 5.09 (p, J = 6.2 Hz, 1H), 1.27 (d, J = 6.3, 6H). ^{19}F-NMR (376 MHz, DMSO-d_6) δ -106.4 (m).

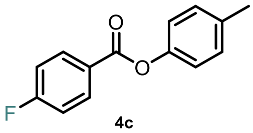

4c

***p*-tolyl-4-fluorobenzoate (4c)**
Prepared according to the previously described procedure using 0.75 mmol (81.0 mg) of *p*-cresol.
^{1}H-NMR (400 MHz, DMSO-d_6) δ 8.25 – 8.12 (m, 2H), 7.46 – 7.35 (m, 2H), 7.28 – 7.21 (m, 2H), 7.13 (m, 2H), 2.31 (s, 3H). ^{19}F-NMR (376 MHz, DMSO-d_6) δ -104.9 (m).

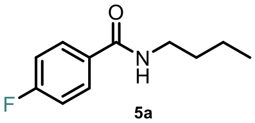

5a

***N*-butyl-4-fluorobenzamide (5a)**
Prepared according to the previously described procedure using 0.75 mmol (54.85 mg, 74 µL) of butylamine.
^{1}H-NMR (400 MHz, DMSO-d_6) δ 8.43 (t, J = 5.7 Hz, 1H), 8.03 – 7.70 (m, 2H), 7.25 (m, 2H), 3.23 (td, J = 7.1, 5.6 Hz, 2H), 1.57 – 1.43 (m, 2H), 1.40 – 1.22 (m, 2H), 0.87 (t, J = 7.3 Hz, 3H).^{19}F-NMR (376 MHz, DMSO-d_6) δ -110.0 (m).

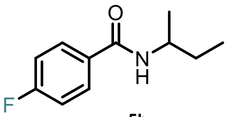

5b

***N*-(sec-butyl)-4-fluorobenzamide (5b)**
Prepared according to the previously described procedure using 0.75 mmol (54.85 mg, 76 µL) of *sec*-butylamine.

^{1}H NMR (400 MHz, DMSO-d_6) δ 8.14 (d, J = 8.2 Hz, 1H), 8.02 – 7.81 (m, 2H), 7.38 – 7.15 (m, 2H), 3.89 (tt, J = 7.8, 6.4 Hz, 1H), 1.58 – 1.39 (m, 2H), 1.11 (d, J = 6.6 Hz, 3H), 0.84 (t, J = 7.4 Hz, 3H).^{19}F-NMR (376 MHz, DMSO-d_6) δ -110.1 (m).

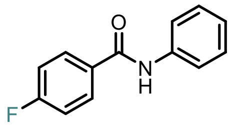

5c

4-fluoro-N-phenylbenzamide (5c)

Prepared according to the previously described procedure using 0.75 mmol (69.84 mg, 64 μL) of aniline.

^{1}H-NMR (400 MHz, DMSO-d_6) δ 10.23 (s, 1H), 8.14 – 7.97 (m, 2H), 7.74 (dt, J = 7.9, 1.1 Hz, 2H), 7.46 – 7.28 (m, 4H), 7.22 – 7.01 (m, 1H).^{19}F-NMR (376 MHz, DMSO-d_6) δ -108.9 (m).

1.4 Procedure for the Detection of Phenol Moieties on F-*Ph*-CDs Surface

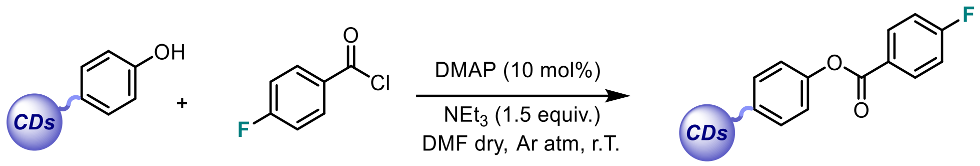

Scheme S3. Reaction scheme for the post-synthetic acylation of *Ph*-CDs for ^{19}F-NMR tagging experiments.

50 mg of *Ph*-CDs were introduced in a flame-dried Schlenk tube followed by dry DMF (10 mL), 4-(Dimethylamino)pyridine (DMAP; 0.4 mmol, 48 mg) and triethylamine (6 mmol, 607 mg, 830 mL). The reaction was placed under argon atmosphere and heated at 40°C. The acylating reagent 4-fluorobenzoyl chloride (4 mmol, 634 mg, 478 mL) was solubilized in 5 mL of dry DMF and added dropwise. The mixture was left to react for 48 hours. Then, it was quenched by the addition of 0.1 M HCl. This caused the precipitation of a fine brown powder. The reaction was extracted with ethyl acetate, in which the functionalized *Ph*-CDs are soluble, contrarily to the pristine material. The acylated *Ph*-CDs were purified *via* flash chromatography. First, the powder is solubilized in 1 mL of DMF and absorbed over silica. A short silica plug was loaded with the powder and eluted with (cyclohexane/ethyl acetate 8:2) to remove the side products. Subsequently, the acylated *Ph*-CDs were desorbed with DMF. The DMF solution was diluted with a solution of 5% w/w of LiCl and extracted with ethyl acetate. The combined organic phases were reunited, dried over Na_2SO_4 and concentrated under reduced pressure. The procedure afforded the purified F-*Ph*-CDs. Specifically, the ^{19}F-NMR spectrum used to quantify the phenol moieties on the nanomaterial is reported in Figure S1.

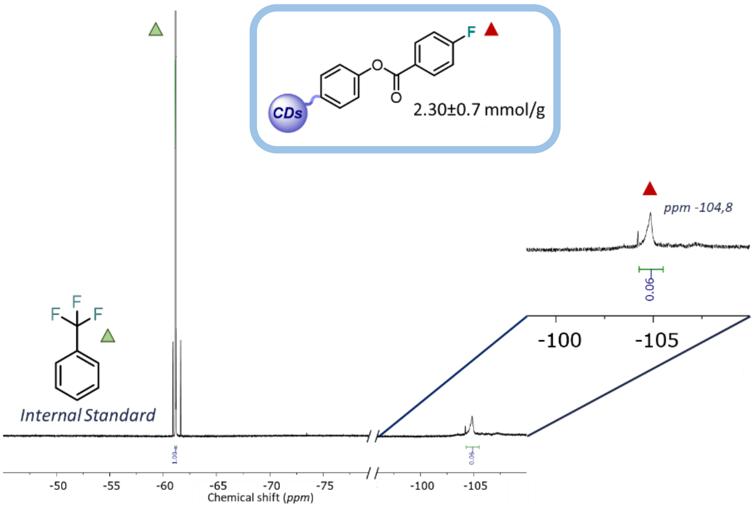

Figure S1. ^{19}F-NMR spectrum of F-*Ph*-CDs recorded in DMSO-d_6 in the presence of α,α,α-trifluorotoluene as internal standard. Inset: magnification of diagnostic peak at -104.8 ppm.

2. Characterization of *Ph*-CDs

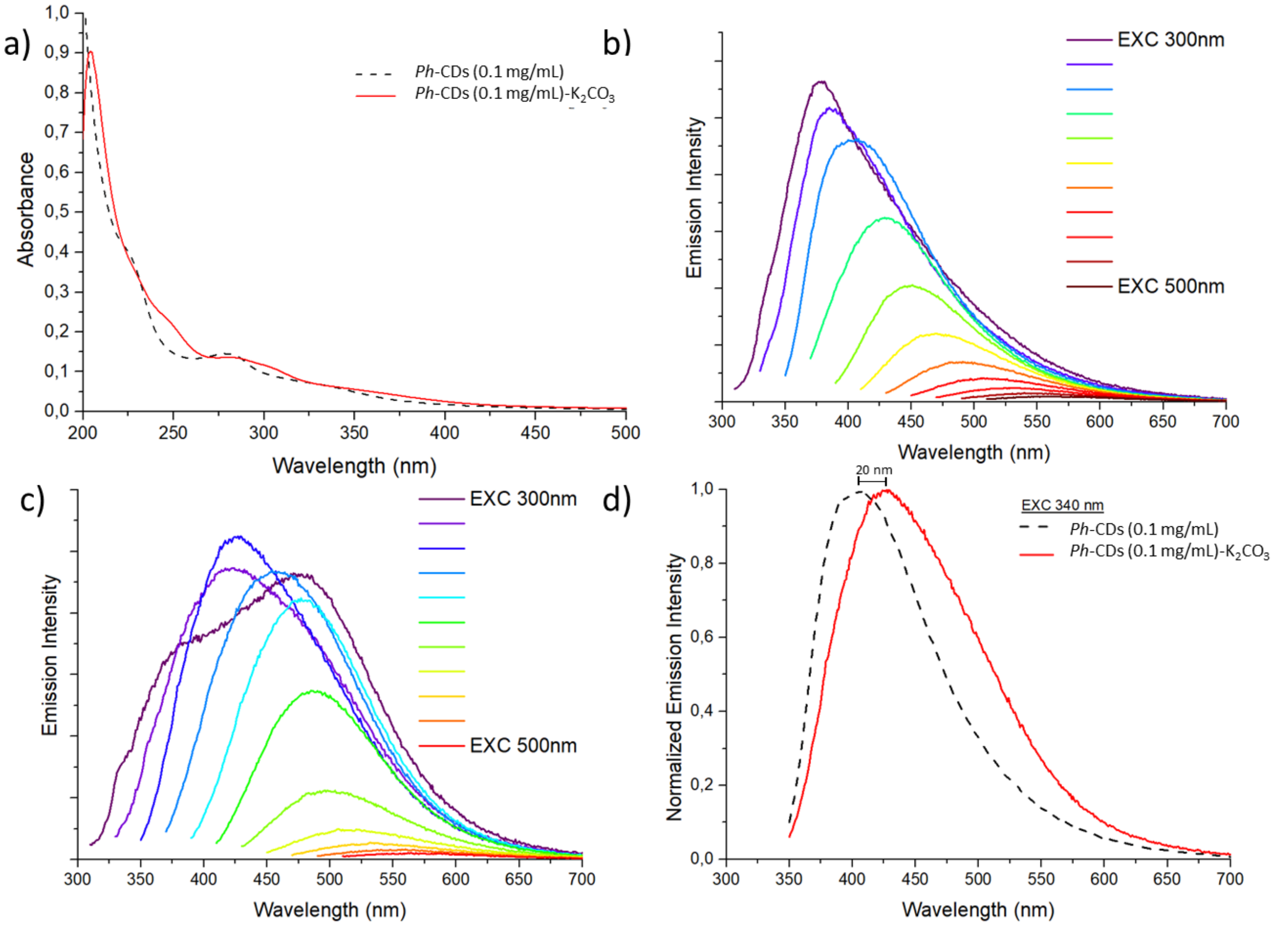

Figure S2. Photophysical characterization of *Ph*-CDs: a) UV-Vis spectra of *Ph*-CDs, dashed-line: *Ph*-CDs pristine, red-line: *Ph*-CDs deprotonated with K_2CO_3 (0.02 M); b) emission map of pristine *Ph*-CDs; c) emission map of *Ph*-CDs deprotonated with K_2CO_3 (0.02 M); d) confrontation of bathochromic shift of pristine and deprotonated carbon dots. All spectra were recorded in MeCN/H_2O (3:1) at a 0.1 mg/mL concentration. MeCN= acetonitrile.

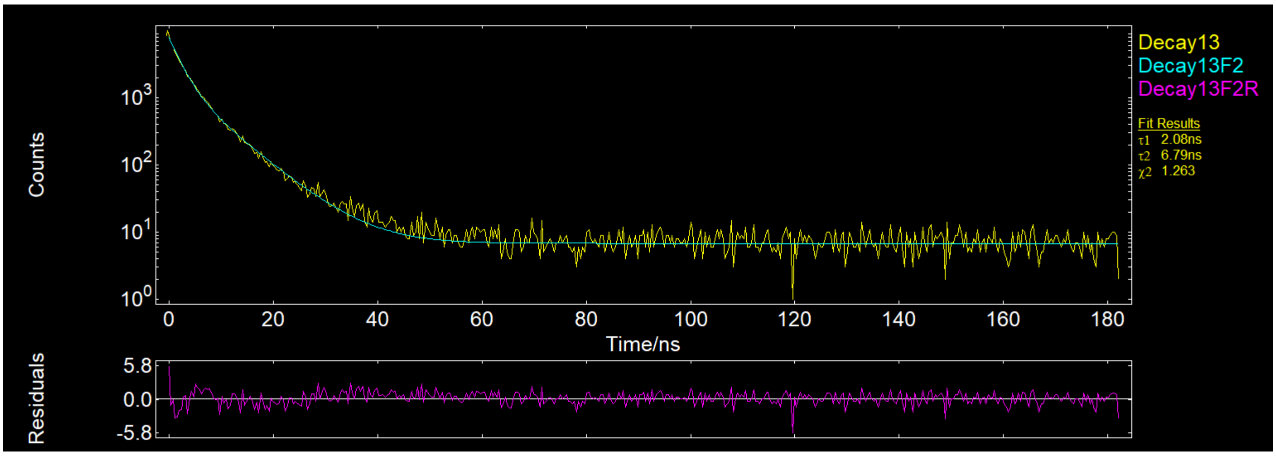

Figure S3. Fluorescence decay curve of *Ph*-CDs in MeCN. The yellow curve corresponds to the sample fluorescence decay, the blue curve corresponds to the best fit, with their respective residuals (green line).

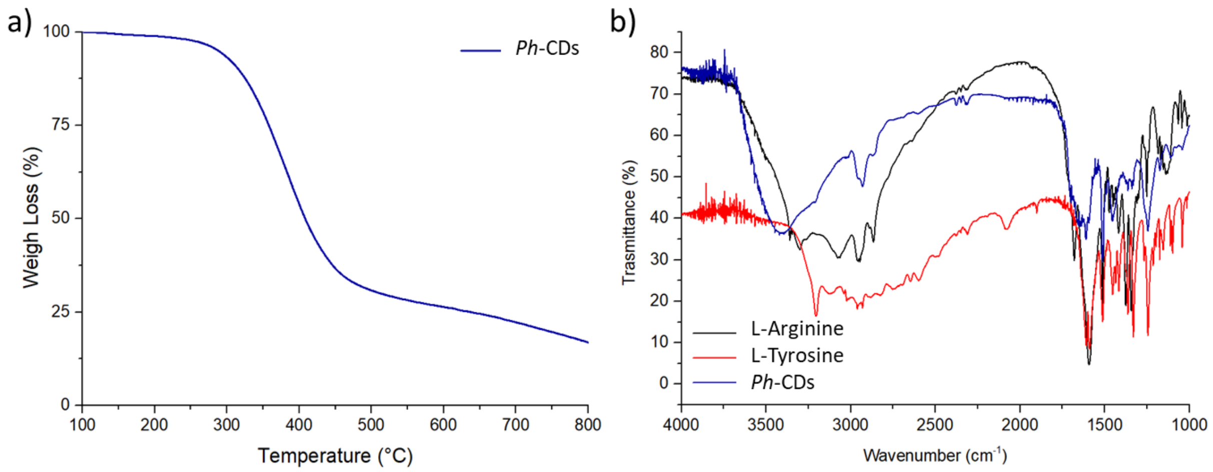

Figure S4. a) Thermogravimetric analysis under nitrogen of *Ph*-CDs; b) ATR-FTIR spectra of *Ph*-CDs (blue line), L-Arginine (black line) and L-Tyrosine (red line).

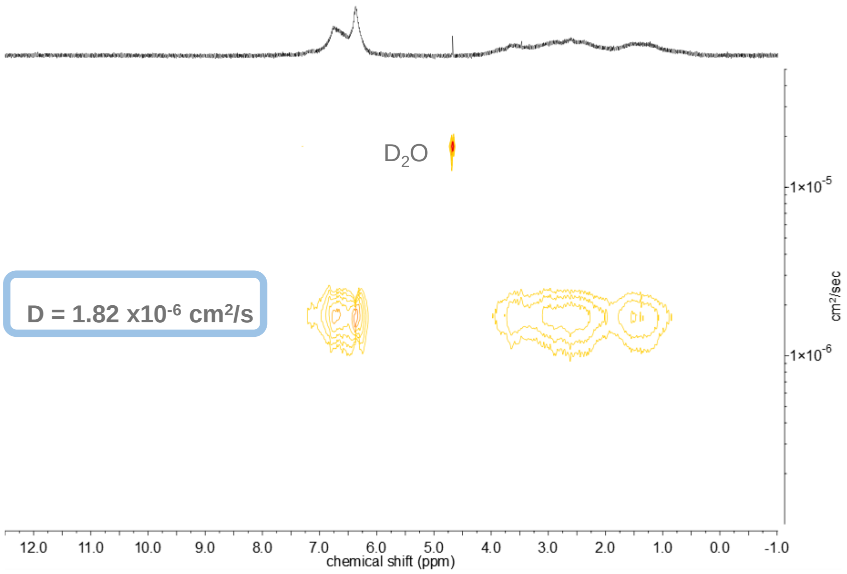

Figure S5. DOSY spectrum of *Ph*-CDs recorded in basic deuterated water (Base: NaOD; 0.2M). Diffusion coefficient (D) = 1.82 x 10^{-6} cm^2/s.

3. General Procedure for the Catalytic Experiments

3.1 General Procedure for the Photocatalytic [3+2] Cycloaddition Reaction

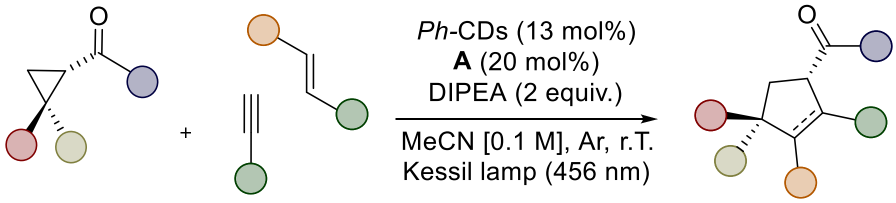

1 1 equiv. **2** 5 equiv. **3**

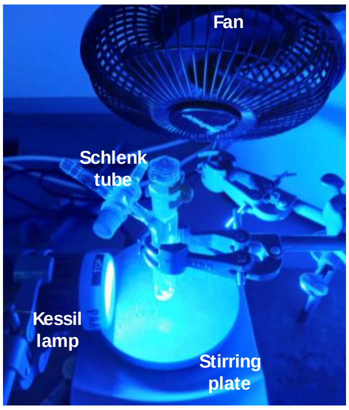

A 10 mL Schlenk tube was charged with cyclopropane **1** (0.1 mmol), unsaturated compounds **2** (0.5 mmol, 5.0 equiv.), *Ph*-CDs (6 mg, 13 mol%), Schreiner thiourea **A** (0.02 mmol, 20 mol%) and DIPEA (0.2 mmol, 2 equiv.). Acetonitrile was then added to the Schlenk tube (1 mL, 0.1 M). The reaction mixture was thoroughly degassed via 3 cycles of freeze-pump-thaw, and the vessel was refilled with argon, placed under light irradiation for 8-30 h (λ = 456 nm). The temperature was kept at around 30°C by using a fan. Then, the reaction mixture was filtered on cotton with Et_2O. The volatiles were removed in vacuo and the residue was purified by column chromatography to give the corresponding products **3**.

***tert*-butyl 3-benzoyl-4-phenylcyclopentane-1-carboxylate (3a)**

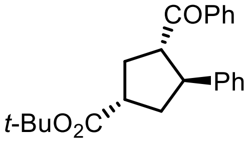

3a

3a was synthesized according to the general procedure from **1a** (24.6 mg, 0.1 mmol) and styrene (58 µL, 0.5 mmol). The reaction was complete after 8 h. The crude product was purified by column chromatography (7:3, Hexane/Et_2O) to give 34 mg (99% yield) of cycloadduct as two separable diastereomers (5:2 d.r.).

<u>Major:</u> **^{1}H-NMR (499 MHz, $CDCl_3$)** δ 7.86 – 7.75 (m, 2H), 7.49 (t, *J* = 7.4 Hz, 1H), 7.37 (t, *J* = 7.8 Hz, 2H), 7.30 – 7.22 (m, 4H), 7.18 – 7.09 (m, 1H), 3.83 (dq, *J* = 34.4, 8.9 Hz, 2H), 3.09 (ddd, *J* = 17.2, 8.4, 6.0 Hz, 1H), 2.57 – 2.45 (m, 2H), 2.28 – 2.18 (m, 1H), 2.13 (dt, *J* = 13.3, 9.0 Hz, 1H), 1.46 (s, *J* = 4.1 Hz, 9H). **^{13}C-NMR (126 MHz, $CDCl_3$)** δ 200.70, 174.35, 143.89, 137.02, 133.05, 128.67, 128.61, 128.53, 127.45, 126.56, 80.66, 54.89, 47.30, 44.62, 37.45, 35.50, 28.22. **HRMS** calculated for $C_{23}H_{26}O_3$ (M-Na): 373.1773 found: 373.1774. The characterization of the compound matches with the data reported in the literature.[1]

<u>Minor</u>: **^{1}H-NMR (499 MHz, $CDCl_3$)** δ 7.59 (dd, *J* = 8.3, 1.2 Hz, 2H), 7.43 – 7.36 (m, 1H), 7.30 – 7.24 (m, 2H), 7.05 – 6.95 (m, 5H), 4.18 (td, *J* = 9.1, 7.3 Hz, 1H), 3.65 (dd, *J* = 17.3, 9.6 Hz, 1H), 2.94 (tt, *J* = 10.5, 8.0 Hz, 1H), 2.65 (ddd, *J* = 13.2, 10.6, 8.7 Hz, 1H), 2.50 – 2.33 (m, 2H), 2.30 – 2.19 (m, 1H), 1.51 (s, 9H). **^{13}C-NMR (101 MHz, $CDCl_3$)** δ 201.07, 174.26, 141.35, 137.95, 132.46, 128.45, 128.24, 128.14, 128.00, 126.49, 80.56, 51.20, 48.99, 44.67, 36.74, 32.52, 28.30. **HRMS** calculated for $C_{23}H_{26}O_3$ (M-Na): 373.1775 found: 373.1774. The characterization of the compound matches with the data reported in the literature.[2]

***tert*-butyl 3-nicotinoyl-4-phenylcyclopentane-1-carboxylate (3b)**

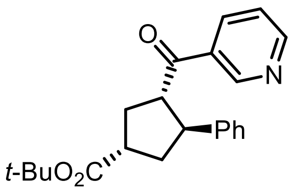

3b

3b was synthesized according to the general procedure from **1b** (24.7 mg, 0.1 mmol) and styrene (58 µL, 0.5 mmol). The reaction was complete after 8 h. The crude product was purified by column chromatography (8:2, Hexane/Et_2O) to give 20 mg (58% yield) of cycloadduct as two separable diastereomers (3:2 d.r.).

<u>Major:</u> **^{1}H-NMR (400 MHz, $CDCl_3$)** δ 8.96 (d, *J* = 1.7 Hz, 1H), 8.69 (dd, *J* = 4.8, 1.7 Hz, 1H), 8.08 – 8.02 (m, 1H), 7.36 – 7.28 (m, 1H), 7.25 – 7.19 (m, 4H), 7.19 – 7.10 (m, 1H), 3.78 (dq, *J* = 26.2, 9.0 Hz, 2H), 3.11 (ddd, *J* = 17.2, 8.1, 5.6 Hz, 1H), 2.57 – 2.44 (m, 2H), 2.33 – 2.23 (m, 1H), 2.16 (dt, *J* = 13.2, 9.1 Hz, 1H), 1.46 (s, *J* = 4.9 Hz, 9H). The characterization of the compound matches with the data reported in the literature.[1]

<u>Minor:</u> **^{1}H-NMR (400 MHz, $CDCl_3$)** δ 8.81 (d, *J* = 1.3 Hz, 1H), 8.57 (d, *J* = 3.5 Hz, 1H), 7.74 (dt, *J* = 7.9, 1.9 Hz, 1H), 7.15 (dd, *J* = 7.9, 4.8 Hz, 1H), 7.05 – 6.92 (m, 5H), 4.15 (dd, *J* = 17.5, 7.8 Hz, 1H), 3.67 (td, *J* = 10.1, 7.4 Hz, 1H), 2.95 (ddd, *J* = 15.7, 10.5, 7.9 Hz, 1H), 2.68 (ddd, *J* = 13.3, 10.4, 8.1 Hz, 1H), 2.51 – 2.33 (m, 2H), 2.27 (dt, *J* = 13.8, 7.7 Hz, 1H), 1.51 (s, 9H). **^{13}C-NMR (101 MHz, $CDCl_3$)** δ 200.25, 174.01, 152.74, 149.52, 140.70, 135.29, 133.19, 128.37, 128.23, 126.82, 123.28, 80.67, 51.47, 49.11, 44.61, 36.60, 32.11, 28.30. **HRMS** calculated for $C_{22}H_{25}NO_3$ (M-H): 352.1906 found: 352.1907.

***tert*-butyl 3-(4-fluorobenzoyl)-4-phenylcyclopentane-1-carboxylate (3c)**

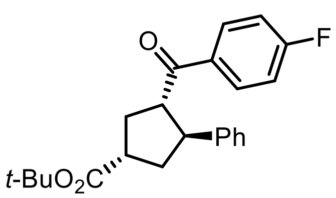

3c

3c was synthesized according to the general procedure from **1c** (26.4 mg, 0.1 mmol) and styrene (58 µL, 0.5 mmol). The reaction was complete after 22 h. The crude product was purified by column chromatography (7:3, Cyhex/DCM) to give 29 mg (97% yield) of cycloadduct as two separable diastereomers (4:1 d.r.)

<u>Major:</u> **^{1}H-NMR (499 MHz, $CDCl_3$)** 7.60 (2H, dd, *J*=8.8, 5.4 Hz), 7.05 – 6.96 (5H, m), 6.92 (2H, t, *J*=8.6 Hz), 4.12 (1H, dd, *J*=16.9, 8.3 Hz), 3.63

(1H, td, J=9.8, 7.5 Hz), 2.93 (1H, tt, J=10.5, 8.0 Hz), 2.64 (1H, ddd, J=13.3, 10.4, 8.4 Hz), 2.40 (2H, qt, J=13.1, 9.0 Hz), 2.23 (1H, dt, J=13.7, 7.6 Hz), 1.51 (9H, s). **^{19}F-NMR (376 MHz, CDCl$_3$)** δ -106.40.**^{13}C-NMR (126 MHz, CDCl$_3$)** δ 199.59, 174.19, 165.32 (d, J=253.7 Hz), 141.10, 134.37 (d, J=3.2 Hz), 130.69 (d, J=9.3 Hz), 128.41, 128.07, 126.63, 115.28 (d, J=21.9 Hz), 51.05, 49.14, 44.62, 36.64, 32.45, 28.30. **HRMS** calculated for $C_{23}H_{25}FO_3$ (M-Na): 391.1680 found: 391.1680.

Minor: **^{1}H-NMR(499 MHz, CDCl$_3$)** 7.80 (2H, ddd, J=8.4, 5.3, 2.6 Hz), 7.25 – 7.19 (4H, m), 7.18 – 7.12 (1H, m), 7.04 – 6.98 (2H, m), 3.84 – 3.69 (2H, m), 3.13 – 3.05 (1H, m), 2.56 – 2.42 (2H, m), 2.25 (1H, ddd, J=13.3, 9.4, 8.2 Hz), 2.13 (1H, dt, J=13.2, 9.0 Hz), 1.46 (9H, s). **^{19}F-NMR (376 MHz, CDCl$_3$)** δ -105.53. **^{13}C-NMR (126 MHz, CDCl$_3$)** δ 199.18, 174.30, 165.75 (d, J=254.7 Hz), 143.73, 133.42 (d, J=2.9 Hz), 131.14 (d, J=9.3 Hz), 128.71, 127.41, 126.66, 115.66 (d, J=21.9 Hz), 80.69, 54.88, 47.58, 44.56, 37.48, 35.27, 28.20. **HRMS** calculated for $C_{23}H_{25}FO_3$ (M-Na): 369.1860 found: 369.1861.

ethyl 3-benzoyl-1-methyl-4-phenylcyclopentane-1-carboxylate (3d)

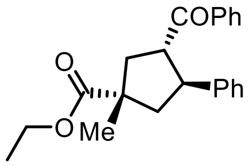

3d

3d was synthesized according to the general procedure from **1d** (22.2 mg, 0.1 mmol) and styrene (58 µL, 0.5 mmol). The reaction was completed after 27 h. The crude product was purified by column chromatography (7:3, Hexane/Et_2O) to give 27 mg (88% yield) of cycloadduct as two not separable diastereomers (5:1 d.r.).

Major: **^{1}H-NMR (400 MHz, CDCl$_3$)** δ 7.82 (d, J = 7.7 Hz, 2H), 7.56 – 7.44 (m, 1H), 7.39 (t, J = 7.8 Hz, 2H), 7.26 (q, 4H), 7.16 (td, J = 5.8, 2.8 Hz, 1H), 4.18 (q, J = 7.1 Hz, 2H), 4.02 – 3.81 (m, 2H), 2.83 (dd, J = 13.0, 7.2 Hz, 1H), 2.58 (dd, J = 13.3, 7.9 Hz, 1H), 2.17 (dd, J = 13.4, 9.0 Hz, 1H), 1.85 (dd, J = 13.1, 11.2 Hz, 1H), 1.47 (s, 3H), 1.28 (t, J = 7.1 Hz, 3H).**^{13}C-NMR (126 MHz, CDCl$_3$)** δ 200.61, 177.34, 143.21, 137.00, 132.99, 128.60, 128.59, 128.52, 127.47, 126.55, 61.02, 54.09, 49.30, 47.31, 45.96, 42.85, 26.34, 14.30. **HRMS** calculated for $C_{22}H_{24}O_3$ (M-H): 337.1798 found: 337.1798.

Major+minor: **^{1}H-NMR (400 MHz, CDCl$_3$)** δ 7.83 – 7.76 (2H major, m), 7.56 – 7.46 (1H major + 2H minor, m), 7.42 – 7.34 (2H major + 1H minor, m), 7.25 – 7.20 (4H major + 2H minor, m), 7.19 – 7.11 (1H major, m), 7.01 – 6.89 (5H minor, m), 4.35 (1H minor, dt, J=10.2, 8.0 Hz), 4.22 (2H minor, q, J=7.1 Hz), 4.18 (2H major, q, J = 7.1 Hz), 3.95 (1H major, td, J=9.6, 7.6 Hz), 3.85 (1H major, ddd, J=11.8, 9.5, 7.4 Hz), 3.80 – 3.72 (1H minor, m), 2.80 (1H major, ddd, J=13.1, 7.4, 1.1 Hz), 2.67 (1H minor, ddd, J=13.1, 7.2, 1.8 Hz), 2.59 (1H major, ddd, J=13.4, 7.7, 1.1 Hz), 2.31 (1H minor, dd, J=13.5, 8.4 Hz), 2.17 (1H major, dd, J=13.4, 9.6 Hz), 2.06 (1H minor, dd, J=13.1, 11.2 Hz), 1.86 (1H major, dd, J=13.1, 11.7 Hz), 1.53 (3H minor, s), 1.47 (3H major, s), 1.32 (3H minor, t, J=7.1 Hz), 1.28 (3H major, t, J = 7.1 Hz). **^{13}C NMR (126 MHz, CDCl$_3$)** δ 201.16, 200.61 (major), 178.14, 177.34 (major), 143.21 (major), 142.83, 138.16, 137.00 (major), 133.17, 132.99 (major), 128.79, 128.63, 128.60 (major), 128.59 (major), 128.52 (major), 128.43, 128.38, 128.15, 127.95, 127.47 (major), 126.55 (major), 126.39, 61.54, 61.02 (major), 54.80, 54.09 (major), 50.72, 49.65, 49.30 (major), 48.83, 47.31 (major), 47.20, 45.96 (major), 45.73, 45.01, 43.40, 42.85 (major), 26.34 (major), 25.64, 14.30 (major), 14.39.

3-(*tert*-butyl) 1-ethyl 5-benzoyl-1-methylcyclopentane-1,3-dicarboxylate (3e)

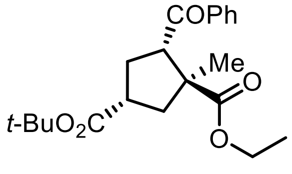

3e

3e was synthesized according to the general procedure from **1a** (24.6 mg, 0.1 mmol) and ethyl methacrylate (55 µL, 0.5 mmol). The reaction was complete after 22 h. The crude product was purified by column chromatography (8:2, Hexane/Et_2O) to give 28 mg (79% yield) of cycloadduct as two separable diastereomers (2:1 d.r.).

Major: **^{1}H-NMR (400 MHz, CDCl$_3$)** δ 7.96 – 7.87 (m, 2H), 7.55 (t, J = 7.3 Hz, 1H), 7.45 (q, J = 7.7 Hz, 2H), 4.41 (dd, J = 10.0, 7.1 Hz, 1H), 4.08 (dd, J = 14.1, 7.0 Hz, 2H), 2.97 – 2.84 (m, 1H), 2.57 – 2.42 (m, 2H), 2.24 – 2.11 (m, 1H), 1.94 (dt, J = 16.0, 8.0 Hz, 1H), 1.47 (s, 9H), 1.17 (t, J = 7.1 Hz, 3H), 1.08 (s, 3H).**^{13}C-NMR (101 MHz, CDCl$_3$)** δ 200.63, 177.13, 173.88, 138.01, 133.23, 128.72, 128.54, 80.63, 61.36, 52.29, 51.55, 43.38, 42.43, 32.55, 28.24, 20.94, 14.15. **HRMS** calculated for $C_{21}H_{28}O_5$ (M-Na): 383.1829 found: 383.1829.

Minor: **^{1}H-NMR (400 MHz, CDCl$_3$)** δ 7.96 – 7.84 (m, 2H), 7.55 (t, J = 7.4 Hz, 1H), 7.45 (t, J = 7.8 Hz, 2H), 4.03 (q, J = 7.2 Hz, 2H), 3.77 (dd, J = 9.1, 5.3 Hz, 1H), 2.98 (dd, J = 17.0, 9.0 Hz, 1H), 2.71 (dd, J = 13.1, 10.3 Hz, 1H), 2.54 – 2.37 (m, 1H), 2.36 – 2.24 (m, 1H), 2.05 (dd, J = 13.1, 8.5 Hz, 1H), 1.40 (s, 9H), 1.37

(s, 3H), 1.09 (t, *J* = 7.1 Hz, 3H). **^{13}C-NMR (101 MHz, $CDCl_3$)** δ 200.81, 176.23, 173.89, 136.69, 132.98, 128.64, 128.61, 80.62, 60.59, 54.89, 52.84, 42.87, 39.24, 32.00, 28.15, 25.65, 14.10. **HRMS** calculated for $C_{21}H_{28}O_5$ (M-Na): 383.1829 found: 383.1828.

***tert*-butyl 3-benzoyl-4-(p-tolyl)cyclopentane-1-carboxylate (3f)**

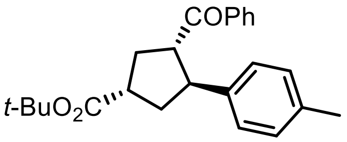

3f

3f was synthesized according to the general procedure from **1a** (24.6 mg, 0.1 mmol) and 4-methylstyrene (59 µL, 0.5 mmol). The reaction was complete after 22 h. The crude product was purified by column chromatography (7:3, Hexane/Et_2O) to give 36 mg (99% yield) of cycloadduct as three separable diastereomers (4:1:1 d.r.).

Major: **^{1}H-NMR (499 MHz, $CDCl_3$)** δ 7.86 – 7.77 (m, 2H), 7.49 (t, *J* = 7.4 Hz, 1H), 7.37 (t, *J* = 7.7 Hz, 2H), 7.12 (d, *J* = 8.0 Hz, 2H), 7.05 (d, *J* = 8.0 Hz, 2H), 3.85 (dd, *J* = 17.7, 8.9 Hz, 1H), 3.76 (q, *J* = 8.7 Hz, 1H), 3.13 – 3.02 (m, 1H), 2.49 (m, 2H), 2.27 (s, 3H), 2.19 (m, 1H), 2.14 – 2.05 (m, 1H), 1.45 (s, 9H). **^{13}C-NMR (126 MHz, $CDCl_3$)** δ 200.84, 174.44, 140.82, 137.03, 136.06, 133.03, 129.33, 128.60, 128.54, 127.29, 80.64, 54.84, 46.90, 44.57, 37.53, 35.45, 28.21, 21.10. **HRMS** calculated for $C_{24}H_{28}O_3$ (M-Na): 387.1932 found: 387.1931. The characterization of the compound matches with the data reported in the literature.[1]

Minor a: **^{1}H-NMR (499 MHz, $CDCl_3$)** δ 7.82 (dd, *J* = 8.3, 1.2 Hz, 2H), 7.52 – 7.46 (m, 1H), 7.40 – 7.34 (m, 2H), 7.15 (d, *J* = 8.1 Hz, 2H), 7.05 (d, *J* = 7.9 Hz, 2H), 3.96 (dd, *J* = 17.2, 9.5 Hz, 1H), 3.70 – 3.58 (m, 1H), 3.04 – 2.93 (m, 1H), 2.55 – 2.43 (m, 2H), 2.27 (s, 3H), 2.15 – 2.03 (m, 2H), 1.48 (s, *J* = 3.9 Hz, 9H). **^{13}C-NMR (126 MHz, $CDCl_3$)** δ 201.68, 175.11, 140.02, 136.81, 136.21, 133.17, 129.33, 128.65, 128.64, 127.41, 80.62, 53.85, 48.10, 44.34, 39.07, 34.97, 28.27, 21.11. **HRMS** calculated for $C_{24}H_{28}O_3$ (M-Na): 387.1931 found: 387.1931.

Minor b: **^{1}H-NMR (499 MHz, $CDCl_3$)** δ 7.59 (t, *J* = 12.6 Hz, 2H), 7.41 (t, *J* = 7.4 Hz, 1H), 7.31 – 7.24 (m, 2H), 6.84 (dd, *J* = 19.5, 8.1 Hz, 4H), 4.14 (td, *J* = 9.1, 7.3 Hz, 1H), 3.63 (dd, *J* = 17.2, 9.4 Hz, 1H), 2.92 (tt, *J* = 10.5, 8.0 Hz, 1H), 2.63 (ddd, *J* = 13.2, 10.6, 9.0 Hz, 1H), 2.49 – 2.32 (m, 2H), 2.21 (dt, *J* = 21.3, 7.4 Hz, 1H), 2.15 (s, 3H), 1.50 (s, 9H). **^{13}C-NMR (126 MHz, $CDCl_3$)** δ 201.10, 174.33, 138.30, 137.98, 135.93, 132.42, 128.68, 128.29, 128.22, 128.19, 80.52, 51.31,48.59, 44.63, 36.79, 32.48, 28.30, 21.01. **HRMS** calculated for $C_{24}H_{28}O_3$ (M-Na): 387.1930 found: 387.1931. The characterization of the compound matches with the data reported in the literature.[2]

***tert*-butyl 4-benzoyl-2-methyl-3-phenylcyclopentane-1-carboxylate (3g)**

3g was synthesized according to the general procedure from **1a** (24.6 mg, 0.1 mmol) and trans-β-Methylstyrene (59 µL, 0.5 mmol). The reaction was complete after 27 h. The crude product was purified by column chromatography (8:2, Hexane/Et_2O) to give 15 mg (28% yield) of cycloadduct as two not separable diastereomers (3:1 d.r.).

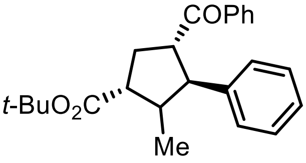

3g

Major: **^{1}H-NMR (499 MHz, $CDCl_3$)** δ 7.79 (d, *J* = 7.5 Hz, 2H), 7.46 (t, *J* = 7.4 Hz, 1H), 7.35 (t, *J* = 7.8 Hz, 2H), 7.27 – 7.22 (m, 4H), 7.15 (dt, *J* = 8.5, 2.3 Hz, 1H), 3.84 (q, *J* = 9.5 Hz, 1H), 3.46 (t, *J*=10.1 Hz, 1H), 3.09 (dd, *J* = 15.5, 8.1 Hz, 1H), 2.61 – 2.50 (m, 1H), 2.47 – 2.37 (m, 1H), 2.29 (ddd, *J* = 13.5, 9.5, 6.5 Hz, 1H), 1.47 (s, 9H), 0.99 (d, *J* = 6.9 Hz, 3H). **^{13}C-NMR (126 MHz, $CDCl_3$)** δ 200.28, 173.63, 142.71, 137.15, 132.83, 128.64, 128.51, 128.50, 127.95, 126.64, 80.85, 54.58, 54.28, 48.85, 44.63, 33.36, 28.36, 15.09. **HRMS** calculated for $C_{24}H_{28}O_3$ (M-Na): 387.1932 found: 387.1931.

Major + minor: **^{1}H-NMR (499 MHz, $CDCl_3$)** δ 7.81 – 7.77 (2H major, m), 7.54 – 7.51 (2H minor, m), 7.46 (1H major, ddt, *J*=7.8, 7.0, 1.3 Hz), 7.37 – 7.31 (2H major + 1H minor, m), 7.29 – 7.19 (4H major + 2H minor, m), 7.15 (1H major, ddt, *J*=6.8, 5.8, 2.2 Hz), 7.03 – 6.97 (2H minor, m), 6.97 – 6.92 (3H minor, m), 4.21 (1H minor, ddd, *J*=10.6, 8.8, 7.3 Hz), 3.85 (1H major, q, *J*=9.5 Hz), 3.46 (1H major, t, *J*=10.1 Hz), 3.16 – 3.02 (1H major + 1H minor, m), 2.73 (1H minor, ddd, *J*=13.0, 11.1, 8.8 Hz), 2.66 – 2.38 (2H major + 2H minor, m), 2.29 (1H major, ddd, *J* = 13.5, 9.5, 6.5 Hz), 2.21 (1H minor, dt, *J*=13.0, 7.4 Hz), 1.52 (9H minor, s), 1.48 (9H major, s), 1.04 (3H minor, d, *J*=6.5 Hz), 1.00 (3H major, d, *J*=6.9 Hz). **^{13}C-NMR (126 MHz, $CDCl_3$)** δ 201.40, 200.29 (major), 173.96, 173.63 (major), 142.70 (major), 140.37, 137.96, 137.14 (major), 132.83 (major), 132.28, 129.02, 128.63 (major), 128.50 (major), 128.49 (major), 128.12, 128.06, 128.00, 127.95 (major), 126.64 (major), 126.52, 80.84 (major), 80.50, 57.26, 54.56 (major), 54.26 (major),

52.45, 50.25, 48.83 (major), 45.28, 44.61 (major), 33.34 (major), 32.42, 28.34 (major), 28.27, 17.81, 15.07 (major).

***tert*-butyl 4-benzoyl-3-phenylcyclopent-2-ene-1-carboxylate (3h)**

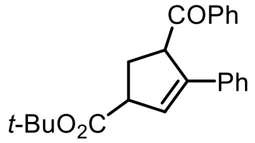

3h

3h was synthesized according to the general procedure from **1a** (24.6 mg, 0.1 mmol) and phenylacetylene (59 µL, 0.5 mmol). The reaction was complete after 20 h. The crude product was purified by column chromatography (8:2, Hexane/Et_2O) to give 33 mg (95% yield) of cycloadduct as two separable diastereomers (2:1 d.r.)

Major: **^{1}H-NMR (499 MHz, $CDCl_3$)** 8.05 (2H, dd, *J*=8.3, 1.4 Hz), 7.65 – 7.58 (1H, m), 7.51 (2H, t, *J*=7.7 Hz), 7.32 – 7.29 (2H, m), 7.25 – 7.16 (3H, m), 6.40 (1H, dd, *J*=2.5, 1.4 Hz), 5.12 (1H, ddt, *J*=10.1, 3.9, 1.8 Hz), 3.79 (1H, ddt, *J*=8.8, 6.5, 2.4 Hz), 2.82 (1H, ddd, *J*=13.3, 10.1, 6.5 Hz), 2.29 (1H, ddd, *J*=13.2, 8.6, 4.2 Hz), 1.48 (9H, s). **^{13}C-NMR (126 MHz, $CDCl_3$)** δ 200.77, 173.05, 143.92, 136.41, 135.00, 133.48, 128.94, 128.81, 128.57, 128.24, 127.83, 126.20, 81.14, 53.06, 51.56, 32.87, 28.28. The characterization of the compound matches with the data reported in the literature.[3]

Minor: **^{1}H-NMR (499 MHz, $CDCl_3$)** 8.04 (2H, dd, *J*=8.3, 1.3 Hz), 7.61 – 7.54 (1H, m), 7.48 (2H, t, *J*=7.7 Hz), 7.32 (2H, dd, *J*=7.2, 1.6 Hz), 7.25 – 7.15 (3H, m), 6.40 (1H, dd, *J*=2.9, 1.6 Hz), 4.90 (1H, ddt, *J*=9.8, 5.9, 1.7 Hz), 3.73 (1H, ddd, *J*=8.4, 4.8, 2.0 Hz), 2.75 (1H, ddd, *J*=13.4, 10.0, 9.0 Hz), 2.49 (1H, dt, *J*=13.4, 5.6 Hz), 1.45 (9H, s).**^{13}C-NMR (126 MHz, $CDCl_3$)** δ 199.94, 172.03, 143.68, 136.41, 135.18, 133.20, 128.86, 128.80, 128.55, 127.81, 127.75, 126.22, 81.16, 53.48, 51.59, 32.38, 28.20. The characterization of the compound matches with the data reported in the literature.[3]

***tert*-butyl 4-benzoyl-3-(4-(tert-butyl)phenyl)cyclopent-2-ene-1-carboxylate (3i)**

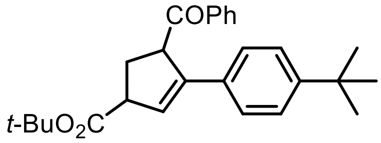

3i

3i was synthesized according to the general procedure from **1a** (24.6 mg, 0.1 mmol) and 4-tert-Butylphenylacetylene (90 µL, 0.5 mmol). The reaction was complete after 22 h. The crude product was purified by column chromatography (8:2, Hexane/Et_2O) to give 40 mg (94% yield) of cycloadduct as two separable diastereomers (5:2 d.r.).

Major: **^{1}H-NMR (101 MHz, $CDCl_3$)** δ 8.09 – 8.01 (2H, m), 7.58 (1H, t, *J*=7.3 Hz), 7.48 (2H, t, *J*=7.6 Hz), 7.25 (4H, m), 6.37 (1H, dd, *J*=2.9, 1.6 Hz), 4.88 (1H, dd, *J*=10.1, 5.6 Hz), 3.71 (1H, t, *J*=4.5 Hz), 2.72 (1H, dt, *J*=13.4, 9.6 Hz), 2.47 (1H, dt, *J*=13.4, 5.4 Hz), 1.55 (9H, s), 1.44 (9H, s). **^{13}C-NMR (101 MHz, $CDCl_3$)** δ 200.06, 172.16, 150.74, 143.38, 136.48, 133.17, 132.24, 128.90, 128.80, 126.96, 125.93, 125.51, 81.10, 53.43, 51.60, 32.35, 31.36, 31.08, 28.19. **HRMS** calculated for $C_{27}H_{32}O_3$ (M-H): 427.2244 found: 427.2244.

Minor **^{1}H-NMR (499 MHz, $CDCl_3$)** δ 8.10 – 8.02 (2H, m), 7.65 – 7.58 (1H, m), 7.51 (2H, t, *J*=7.7 Hz), 7.24 (4H, m), 6.36 (1H, dd, *J*=2.5, 1.4 Hz), 5.18 – 5.05 (1H, m), 3.78 (1H, ddt, *J*=8.7, 6.4, 2.3 Hz), 2.81 (1H, ddd, *J*=13.3, 10.1, 6.4 Hz), 2.26 (1H, ddd, *J*=13.1, 8.6, 4.3 Hz), 1.47 (9H, s), 1.26 (9H, s). **^{13}C-NMR (126 MHz, $CDCl_3$)** δ 200.96, 173.12, 150.82, 143.67, 136.47, 133.44, 132.10, 128.93, 128.82, 127.41, 125.88, 125.51, 81.06, 53.01, 51.57, 34.66, 32.85, 31.36, 28.28. **HRMS** calculated for $C_{27}H_{32}O_3$ (M-H): 427.2244 found: 427.2243.

***tert*-butyl 3-(4-aminophenyl)-4-benzoylcyclopent-2-ene-1-carboxylate (3j)**

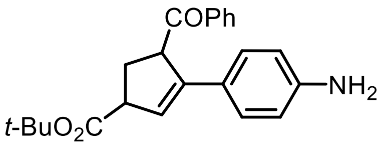

3j

3j was synthesized according to the general procedure from **1a** (24.6 mg, 0.1 mmol) and 4-ethynylaniline (58 mg, 0.5 mmol). The reaction was complete after 48 h. The crude product was purified by column chromatography (8:2, Cyhex/EtOAc) to give 33 mg (93% yield) as two not separable diastereoisomer cycloadducts (5:2 d.r.).

Major: **^{1}H-NMR (400 MHz, $CDCl_3$)** δ 8.05 – 7.98 (m, 2H), 7.63 – 7.52 (m, 1H), 7.52 – 7.40 (m, 2H), 7.12 (d, *J* = 8.6 Hz, 2H), 6.56 (d, *J* = 8.6 Hz, 2H), 6.21 (dd, *J* = 2.9, 1.6 Hz, 1H), 4.81 (ddt, *J* = 10.0, 5.9, 1.8 Hz, 1H), 3.70 (dddd, *J* = 8.6, 5.0, 2.9, 1.8 Hz, 1H), 2.71 (ddd, *J* = 13.5, 10.0, 9.1 Hz, 1H), 2.46 (dt, *J* = 13.4, 5.6 Hz, 1H), 1.43 (s, 9H).**^{13}C-NMR (126 MHz, $CDCl_3$)** δ 200.30, 172.41, 146.09, 143.40, 136.50, 133.06, 128.90, 128.73, 127.41, 124.31,

115.08, 113.86, 80.96, 53.84, 51.52, 32.35, 28.20. **HRMS** calculated for $C_{23}H_{25}NO_3$ (M-Na): 386.1727 found: 386.1729.

Minor + major: **^{1}H-NMR (400 MHz, $CDCl_3$)** δ 8.04 (m, 4H (2Hmajor + 2Hminor)), 7.62 – 7.53 (m, 2H(1Hmajor + 1Hminor)), 7.53 – 7.41 (m, 4H (2Hmajor + 2Hminor)), 7.11 (dd, *J* = 8.5, 6.2 Hz, 4H (2Hmajor + 2Hminor)), 6.58 – 6.50 (m, 4H (2Hmajor + 2Hminor)), 6.21 (dt, *J* = 3.0, 1.4 Hz, 2H (1Hmajor + 1Hminor)), 5.04 (ddt, *J* = 10.3, 3.9, 1.7 Hz, 1H minor), 3.75 (ddt, *J* = 8.8, 6.5, 2.3 Hz, 1H minor), 3.07 (broad, 2H), 2.85 – 2.64 (m, 2H (1Hmajor + 1Hminor)), 2.46 (m, 2H (1Hmajor + 1Hminor)), 2.25 (ddd, *J* = 13.3, 8.5, 4.1 Hz, 1H), 1.47 (s, 9H). **^{13}C-NMR (101 MHz, $CDCl_3$)** δ 200.35, 200.30 (major) 172.43, 172.41 (major), 146.09 (major), 145.70, 143.56, 143.40 (major), 136.53, 136.50 (major), 133.37, 133.06 (major), 128.90 (major), 128.80, 128.73 (major), 127.41 (major), 127.39, 126.08, 124.31(major), 124.84, 115.31, 115.08 (major), 113.86 (major), 110.15, 80.96 (major), 80.95, 53.84 (major), 53.12, 51.52 (major), 51.45, 32.84, 32.35 (major), 28.27, 28.20 (major).

ethyl 4-benzoyl-1-methyl-3-phenylcyclopent-2-ene-1-carboxylate (3k)

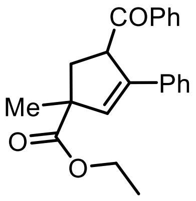

3k

3k was synthesized according to the general procedure from **1d** (22.2 mg, 0.1 mmol) and phenylacetylene (59 mg, 0.5 mmol). The reaction was complete after 24 h. The crude product was purified by column chromatography (9:1, Hexane/Et_2O) to give 23 mg (52% yield) of cycloadduct as two separable diastereomers (2:1 d.r.).

Major: **^{1}H-NMR (400 MHz, $CDCl_3$)** δ 8.03 (dt, *J* = 8.5, 1.7 Hz, 2H), 7.61 – 7.55 (m, 1H), 7.51 – 7.45 (m, 2H), 7.35 – 7.29 (m, 2H), 7.25 – 7.16 (m, 3H), 6.39 (d, *J* = 1.5 Hz, 1H), 4.97 (ddd, *J* = 9.8, 5.0, 1.4 Hz, 1H), 4.13 (dtt, *J* = 10.8, 7.3, 3.7 Hz, 2H), 2.81 (dd, *J* = 13.4, 5.1 Hz, 1H), 2.38 (dd, *J* = 13.4, 9.9 Hz, 1H), 1.47 (s, 3H), 1.24 (t, *J* = 7.1 Hz, 3H). **^{13}C-NMR (101 MHz, $CDCl_3$)** δ 199.87, 175.55, 141.86, 136.41, 135.02, 133.40, 133.23, 128.82, 128.58, 127.84, 126.22, 110.15, 61.11, 55.54, 53.13, 40.45, 25.95, 14.27. **HRMS** calculated for $C_{22}H_{22}O_3$ (M-Na): 357.1461 found: 357.1461.

Minor: **^{1}H-NMR (400 MHz, $CDCl_3$)** δ 8.09 – 8.01 (m, 2H), 7.64 – 7.59 (m, 1H), 7.56 – 7.43 (m, 3H), 7.32 – 7.27 (m, 2H), 7.26 – 7.17 (m, 2H), 6.36 (d, *J* = 1.6 Hz, 1H), 5.12 (ddd, *J* = 10.2, 5.3, 1.6 Hz, 1H), 4.26 – 4.07 (m, 2H), 3.20 (dd, *J* = 13.4, 10.2 Hz, 1H), 1.94 (dd, *J* = 13.4, 5.4 Hz, 1H), 1.45 (s, 3H), 1.28 (t, *J* = 7.1 Hz, 3H). **^{13}C-NMR (101 MHz, $CDCl_3$)** δ 201.13, 176.23, 142.36, 136.42, 135.00, 133.49, 133.26, 128.83, 128.57, 127.83, 126.22, 110.15, 61.15, 56.18, 53.01, 40.40, 25.49, 14.38. **HRMS** calculated for $C_{22}H_{22}O_3$ (M-Na): 357.1461 found: 357.1462.

***tert*-butyl 4-(4-fluorobenzoyl)-3-phenylcyclopent-2-ene-1-carboxylate (3l)**

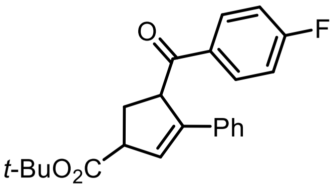

3l

3l was synthesized according to the general procedure from **1c** (26.4 mg, 0.1 mmol) and phenylacetylene (90 µL, 0.5 mmol). The reaction was complete after 30 h. The crude product was purified by column chromatography (9:1, Hexane/Et_2O) to give 28 mg (78% yield) of cycloadduct as two separable diastereomers (5:2 d.r.).

Major: **^{1}H-NMR (499 MHz, $CDCl_3$)** δ 8.09 – 8.03 (2H, m), 7.32 – 7.28 (2H, m), 7.25 – 7.22 (2H, m), 7.21 – 7.17 (1H, m), 7.17 – 7.10 (2H, m), 6.39 (1H, dd, *J*=2.9, 1.7 Hz), 4.83 (1H, ddt, *J*=9.6, 5.8, 1.8 Hz), 3.73 (1H, dddd, *J*=8.8, 5.1, 2.8, 1.8 Hz), 2.74 (1H, ddd, *J*=13.4, 10.0, 9.0 Hz), 2.48 (1H, dt, *J*=13.4, 5.6 Hz), 1.45 (9H, s). **^{19}F NMR (376 MHz, $CDCl_3$)** δ -105.31. **^{13}C-NMR (126 MHz, $CDCl_3$)** δ 198.43, 172.00, 165.86 (d, *J* = 254.9 Hz), 143.63, 135.09, 132.74 (d, *J* = 3.0 Hz), 131.52 (d, *J* = 9.3 Hz), 128.60, 128.01, 127.86, 126.19, 115.90 (d, *J* = 21.8 Hz), 81.24, 53.73, 51.57, 32.28, 28.21. **HRMS** calculated for $C_{23}H_{23}FO_3$ (M-Na): 389.1523 found: 389.1522.

Minor: **^{1}H-NMR (499 MHz, $CDCl_3$)** δ 8.10 – 8.04 (2H, m), 7.31 – 7.22 (5H, m), 7.22 – 7.14 (2H, m), 6.39 (1H, dd, *J*=2.5, 1.4 Hz), 5.10 – 5.05 (1H, m), 3.79 (1H, ddt, *J*=8.7, 6.4, 2.4 Hz), 2.82 (1H, ddd, *J*=13.4, 10.1, 6.4 Hz), 2.27 (1H, ddd, *J*=13.2, 8.6, 4.3 Hz), 1.48 (9H, s). **^{19}F NMR (376 MHz, $CDCl_3$)** δ -104.77. **^{13}C-NMR (126 MHz, $CDCl_3$)** δ 199.22, 172.95, 166.06 (d, *J* = 255.6 Hz), 143.80, 134.94, 132.81 (d, *J* = 3.0 Hz), 131.46 (d, *J* = 9.3 Hz), 128.60, 128.33, 127.91, 126.16, 116.07 (d, *J* = 21.9 Hz), 81.22, 53.05, 51.56, 32.80, 28.28. **HRMS** calculated for $C_{23}H_{23}FO_3$ (M-Na): 389.1523 found: 389.1524.

tert-butyl-4-nicotinoyl-3-phenylcyclopent-2-ene-1-carboxylate (3m)

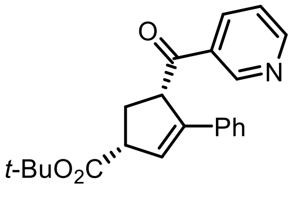

3m

3m was synthesized according to the general procedure from **1b** (24.7 mg, 0.1 mmol) and phenylacetylene (59 mg, 0.5 mmol). The reaction was complete after 24 h. The crude product was purified by column chromatography (2:1, Cyclohex/EtOAc) to give 25 mg (66% yield) of cycloadduct as two separable diastereomers (1:1 d.r.).

Major: **^{1}H-NMR (400 MHz, $CDCl_3$)** δ 9.28 (s, 1H), 8.84 (s, 1H), 8.38 (d, *J* = 7.8 Hz, 1H), 7.58 – 7.52 (m, 1H), 7.31 – 7.18 (m, 5H), 6.41 (dd, *J* = 2.5, 1.4 Hz, 1H), 5.07 (d, *J* = 9.9 Hz, 1H), 3.81 (dd, *J* = 8.7, 6.4 Hz, 1H), 2.86 (ddd, *J* = 13.5, 10.1, 6.5 Hz, 1H), 2.31 (ddd, *J* = 13.2, 8.6, 4.3 Hz, 1H), 1.48 (s, 9H).**^{13}C-NMR (126 MHz, $CDCl_3$)** δ 199.64, 172.73, 153.50, 149.84, 143.36, 136.48, 134.75, 131.83, 128.72, 128.68, 128.06, 126.20, 124.17, 81.36, 53.70, 51.58, 32.44, 28.25. **HRMS** calculated for $C_{22}H_{23}NO_3$ (M-H): 350.1751 found: 350.1752.

Minor: **^{1}H-NMR (400 MHz, $CDCl_3$)** δ 9.26 (1H, s), 8.79 (1H, m), 8.34 (1H, dt, *J*=8.0, 1.9 Hz), 7.47 (1H, m), 7.33 – 7.20 (5H, m), 6.40 (1H, dd, *J*=2.9, 1.6 Hz), 4.84 (1H, ddt, *J*=9.9, 5.4, 1.6 Hz), 3.82 – 3.72 (1H, m), 2.77 (1H, ddd, *J*=13.5, 10.2, 9.1 Hz), 2.52 (1H, dt, *J*=13.5, 5.3 Hz), 1.45 (9H, s). **^{13}C NMR (126 MHz, $CDCl_3$)** δ 198.90, 171.86, 153.57, 150.21, 143.16, 136.32, 134.91, 131.66, 128.69, 128.21, 128.01, 126.21, 123.87, 81.38, 54.22, 51.59, 31.93, 28.20. **HRMS** calculated for $C_{22}H_{23}NO_3$ (M-Na): 372.1570 found: 372.1572.

3.2 Screening of the Conditions of the [3+2] Cycloaddition

We screened different bases for the [3+2] cycloaddition between **1a** and **2a**. Interestingly, DIPEA significantly outperformed all the bases tested (Table S1). Indeed, DIPEA might play a dual role as this tertiary amine can act (*i*) as a base to deprotonate the phenol moieties on *Ph*-CDs and (*ii*) as an electron donor to close the photocatalytic cycle.

Table S1. Comparative study on different bases in the [3+2] cycloaddition reaction between **2a** and **1a** (reaction time: 24 h). The general procedure of this reaction is described in Section 3.1.

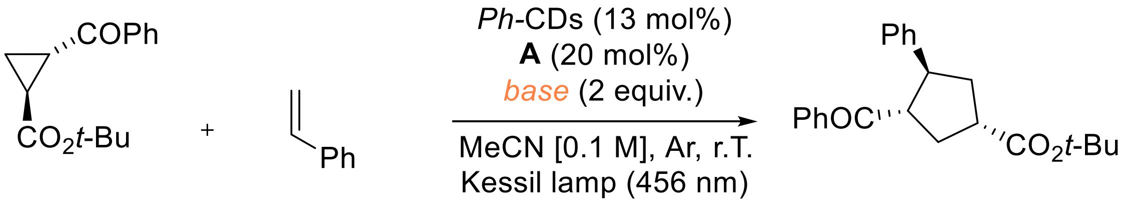

1a 0.1 mmol   **2a** 0.5 mmol   **3a**

Entry	Base	Yield % (d.r.)[a]
1	DIPEA	98 (3:1)
2	DABCO	0
3	TMG	<5
4[b]	NaO*t*-Bu	9
5[b]	Cs_2CO_3	0

[a] ^{1}H-NMR based yields using 1,3,5-trimethoxybenzene as the internal standard. [b] Reactions performed without **A**.

We also performed the [3+2] cycloaddition between **1a** and **2a** using the chiral thioureas **A_1** and **A_2**. Unfortunately, no satisfying results in terms of enantiomeric excess (*ee*) were obtained (Table S2).

Table S2. Study of enantioselective version of [3+2] cycloaddition reaction between **2a** and **1a** using enantiopure thioureas. The general procedure of this reaction is described in Section 3.1.

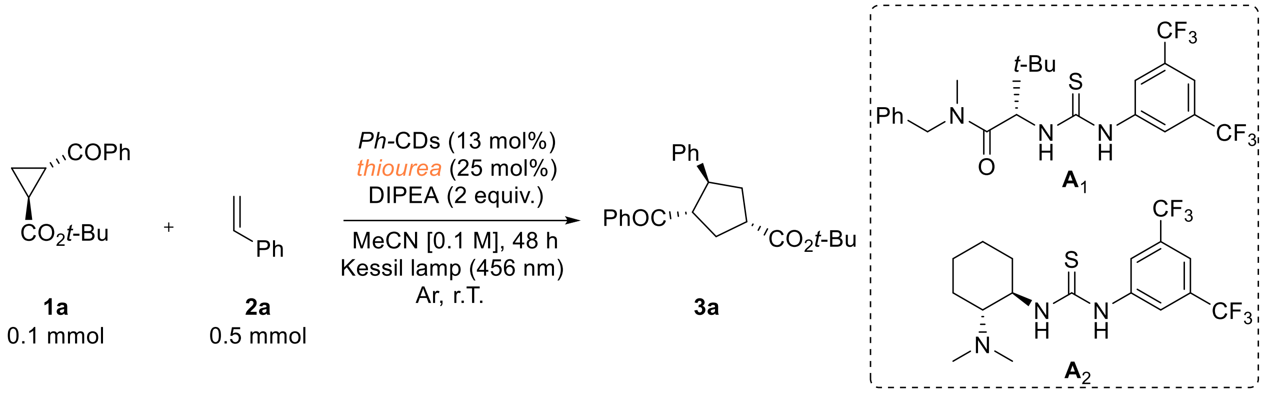

Entry	Thiourea	Yield % [a]	*ee* (**3a** major) [%][b]
1	(*S*)-2-[[3,5-Bis(trifluoromethyl)phenyl]thioureido]-*N*-benzyl-*N*-3,3-trimethylbutanamide (**A**1)	68	7
2	1-[3,5-bis(trifluoromethyl)phenyl]-3-[(1*R*,2*R*)-(-)-2-(dimethylamino)cyclohexyl]thiourea (**A**2)	55	0

[a] The yield was determined by ^{1}H-NMR analysis on the crude mixture using 1,3,5-trimethoxybenzene as internal standard. [b] The enantiomeric excess was evaluated on the crude mixture. HPLC: Phenomenex AD-H; *n*-hexane/iPrOH, 10 to 20% iPrOH, flow-rate 0.75 mL/min; t_1 = 11.6 min; t_2 = 14.4 min.

3.3 Monitoring of the Catalytic Adduct

To confirm the formation of a photocatalytic active complex between *Ph*-CDs, **A** and DIPEA in the initial 2 h of the reaction, we performed an experiment as follows: first (*i*) *Ph*-CDs, **A** and DIPEA were mixed in acetonitrile for 2 h to assess the formation of the photocatalytic system under 456 nm irradiation, then (*ii*) the reactants (**1a** and **2a**) were added to this solution which was stirred for additional 6 h under light irradiation. This experiment almost provided a quantitative yield of product **3a** (Figure S6).

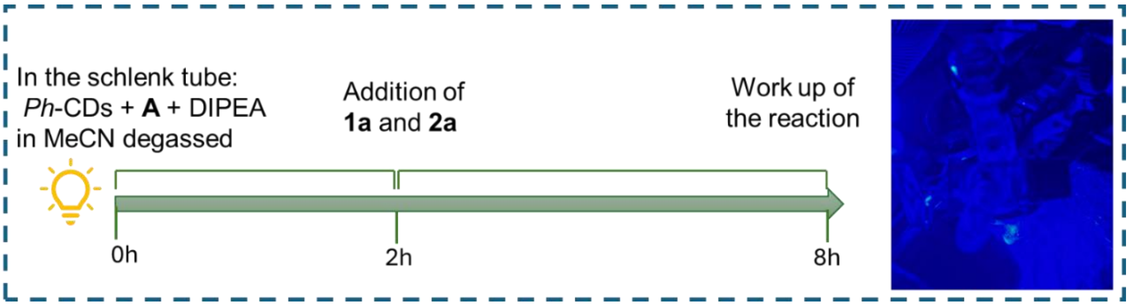

Figure S6. Illustration of the experiment about the formation of the catalytic adduct (*Ph*-CDs + **A** + DIPEA).

3.4 Stern-Volmer Quenching Study

To further demonstrate the feasibility of a single electron transfer (SET) between the catalytic system (composed by *Ph*-CDs, **A** and DIPEA) and cyclopropane **1a**, a series of Stern-Volmer quenching studies were performed in acetonitrile. The emission spectrum was obtained upon excitation at 456 nm and showed a decreased intensity when **1a** was added (Figure S7). The fluorescence quenching turned out to be linear in

the range 25-80 mM. The Stern-Volmer constant (K_{sv}) was derived by the slope of equation below, which is 4.66 M^{-1}.

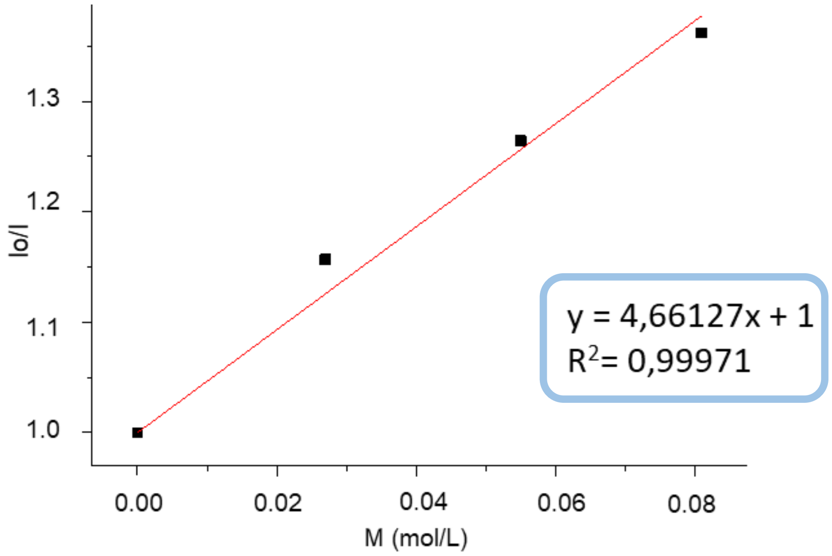

Figure S7. Stern-Volmer quenching study.

4. UV-Vis Experiments

4.1 Absorption Spectra

We carried out UV-Vis experiments of each reaction component (**1a**, **2a**, *Ph*-CDs, DIPEA, **A**) of the [3+2] cycloaddition and their relative mixtures in acetonitrile (Figure S8). Within these experiments, the possible formation of an EDA complex between the components of the reaction was excluded. A red-shifted absorption was observed only for the solution containing *Ph*-CDs + **A** + DIPEA.

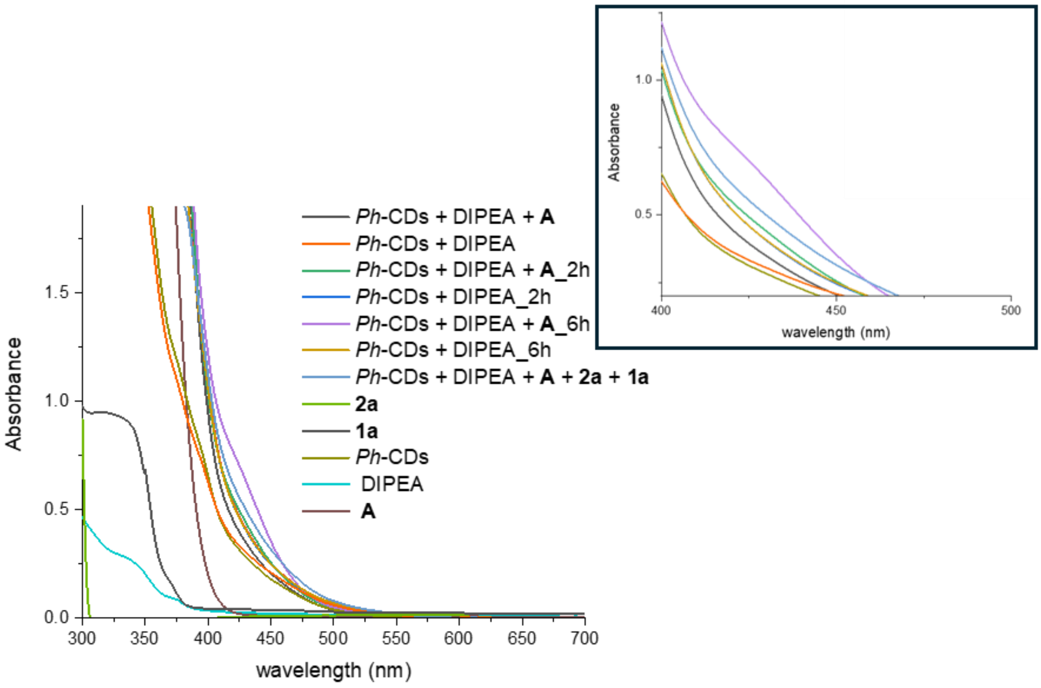

Figure S8. Optical absorption spectra recorded in MeCN: [DIPEA] = 100 mM; [**A**] = 10 mM; [*Ph*-CDs] = 50 mM; [**1a**] = 50 mM; [**2a**] = 250 mM. Inset: spectrum magnification between 400-500 nm.

Absorption Spectra with Molecular Phenol

To test the behaviour of molecular phenols in the presence of DIPEA and **A** in acetonitrile, we carried out UV-Vis experiments using the 4-*tert*-butylphenol as molecular phenol (Figure S9). We recorded absorption spectra of the components (4-*tert*-butylphenol red line, **A** black line, DIPEA blue line) and the mixture containing 4-*tert*-butylphenol + **A** + DIPEA. In particular, a red-shifted absorption was observed after 2 h (violet line) and after 5 h (yellow line) for the solution containing 4-*tert*-butylphenol + **A** + DIPEA.

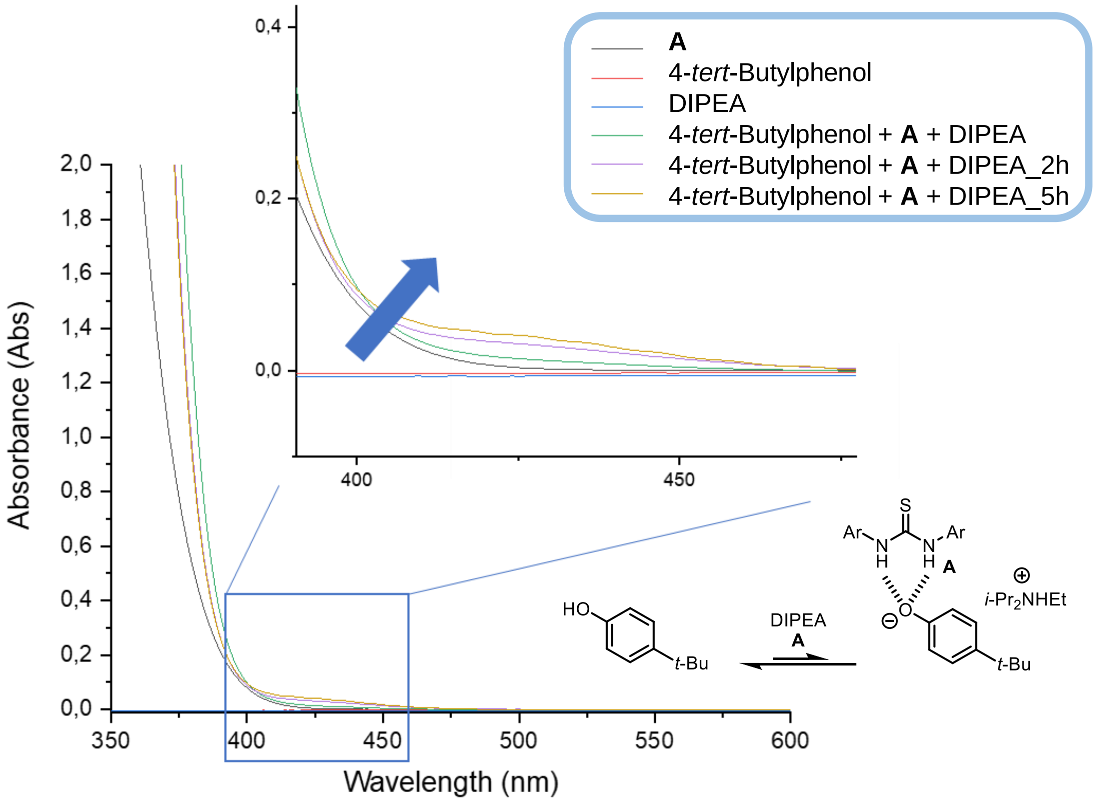

Figure S9. Optical absorption spectra recorded in MeCN: [DIPEA] = 100 mM; [**A**] = 10 mM; [4-*tert*-butylphenol] = 50 mM.

5. Recycling Experiments

1. To conduct the recycling tests, we selected cyclopropane **1a** and styrene **2a** as substrates, performing the reaction under the optimized conditions for 16 hours. The recyclability procedure is illustrated in Figure S10. The reaction was carried out in a Schlenk tube that could be directly attached to rotavapor equipment. After the reaction, the solvent was removed under reduced pressure. The resulting crude mixture (solid powder) was washed with diethyl ether (2 mL) and sonicated to precipitate the *Ph*-CDs. The solid was allowed to settle, and the supernatant containing the molecular species was removed, leaving the catalyst in the same reaction vessel. This extraction process using diethyl ether was repeated three times.

2. Subsequently, the recovered *Ph*-CDs were dried and reused for up to three photocatalytic reactions in the same reaction vessel. Meanwhile, the combined organic phases were collected and dried over Na_2SO_4. The crude product was then dissolved in a deuterated solvent, spiked with an internal standard, and analyzed via NMR.

3. At the end of the recycling process, the catalyst was recovered by filtration through a PTFE membrane , washed with an aqueous solution of HCl (0.1 M) and dried. Notably, the recyclability tests were performed three times to evaluate the reproducibility of the process and assess standard deviations. The reaction yield

of each catalytic cycle along with the corresponding error bar are shown within Figure S11. Furthermore, UV-Vis and fluorescence analyses of *Ph*-CDs obtained after three recycling cycles were recorded (Figure S12).

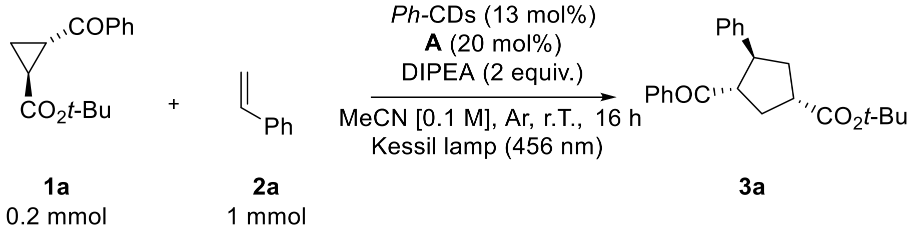

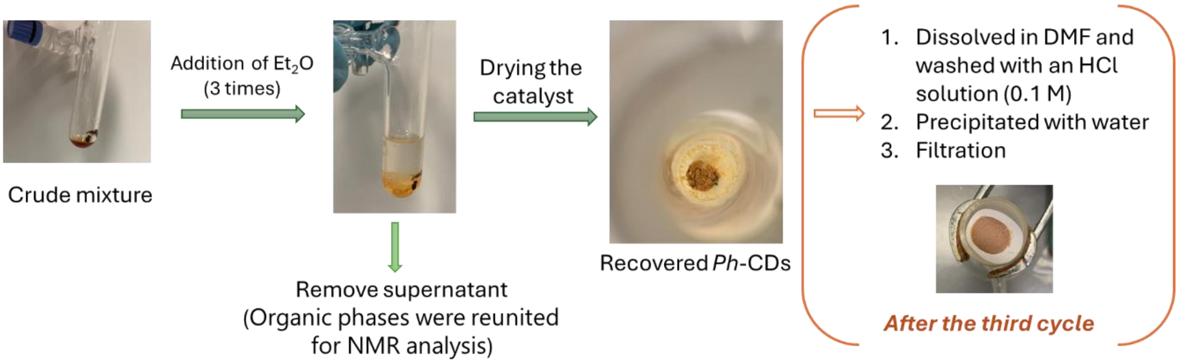

Figure S10. Illustration of the recycling test procedure.

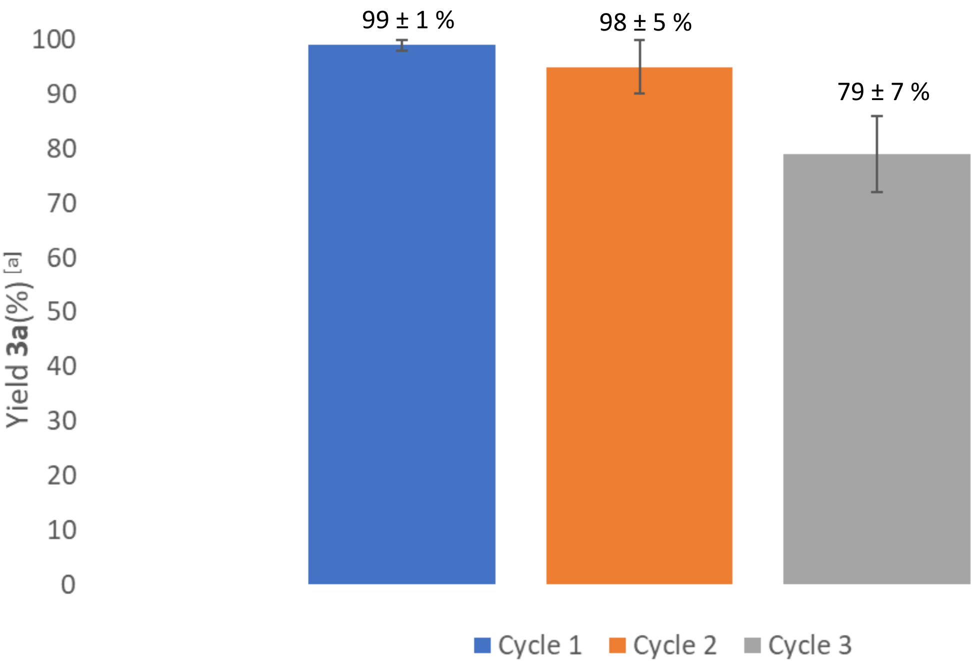

Figure S11. Histogram of the reaction yields of **3a** after each recycling cycle.

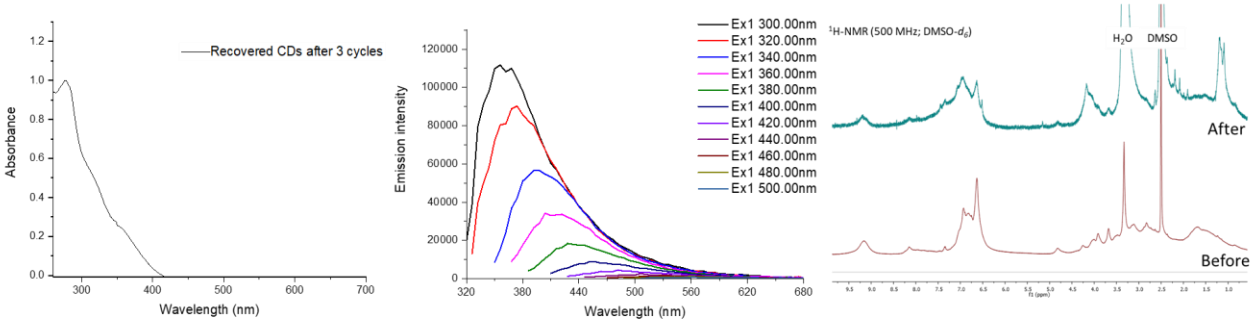

Figure S12. Optical absorption and fluorescence in MeCN of recovered *Ph*-CDs after 3 recycling cycles. All UV-Vis and fluorescence spectra were measured in MeCN at 0.1 mg/mL concentration of *Ph*-CDs. ^{1}H-NMR spectra in DMSO-d_6 of *Ph*-CDs before and after 3 recycling cycles.

6. Kinetic Study of the [3+2] Cycloaddition

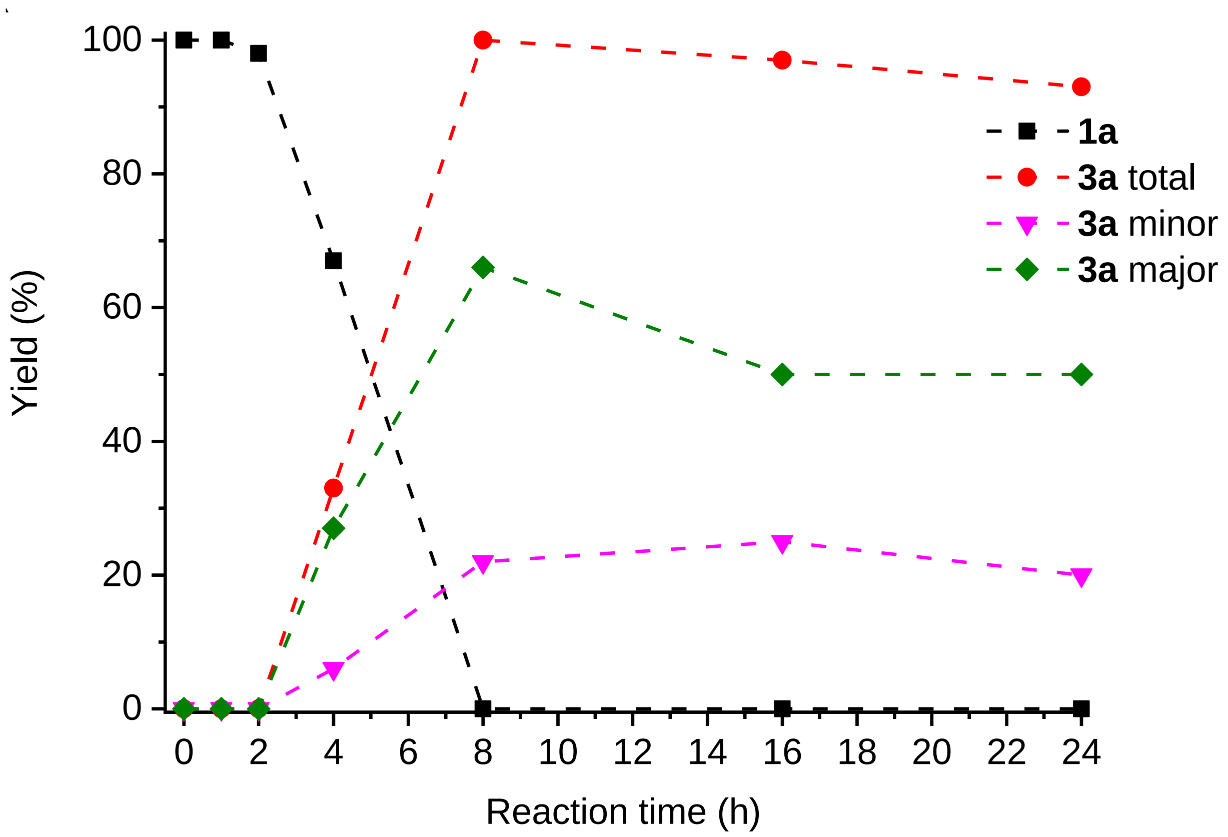

Figure S13. Kinetic study of the model reaction over time. **3a** major: major diastereoisomer of **3a**; **3a** minor: minor diastereoisomer of **3a**; **3a** total: overall yield of **3a**. The yield was determined by ^{1}H-NMR analysis on the crude mixture using 1,3,5-trimethoxybenzene as internal standard.

7. NMR Spectra

7.1 ^{1}H -^{13}C-^{19}F NMR

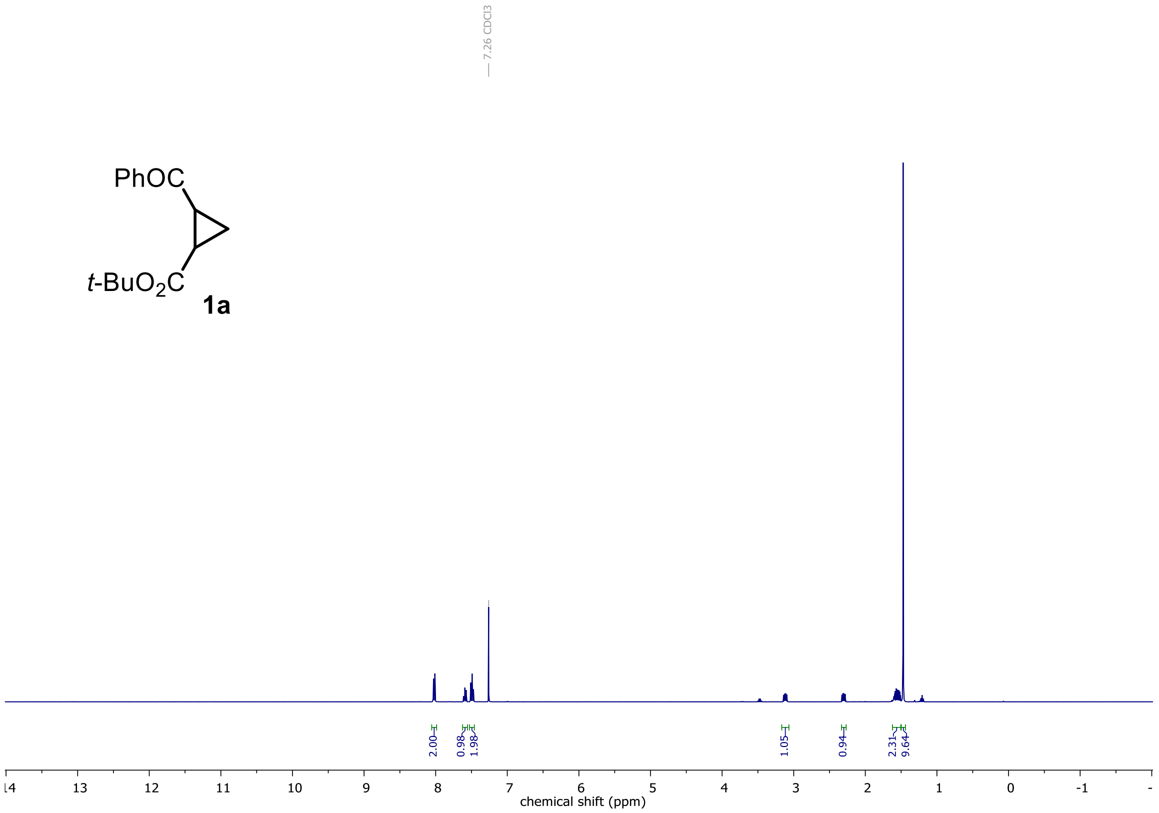

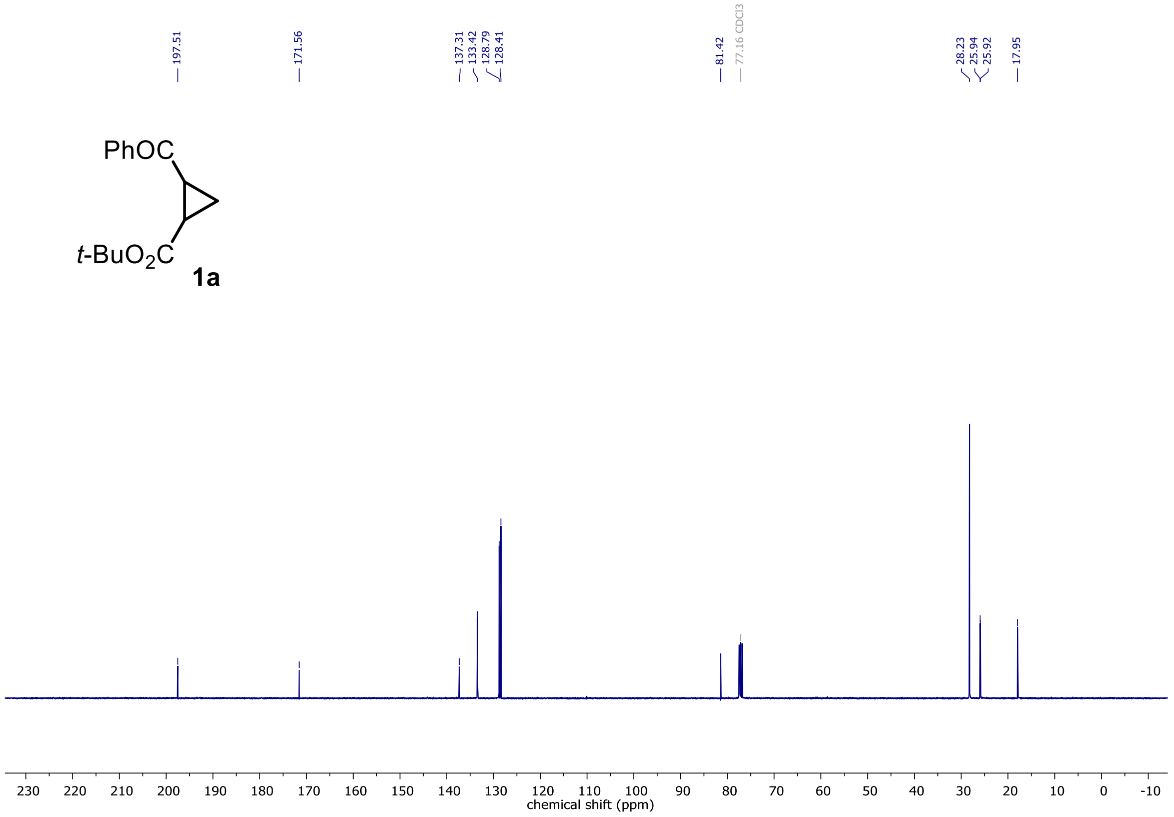

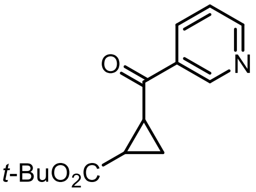

1b

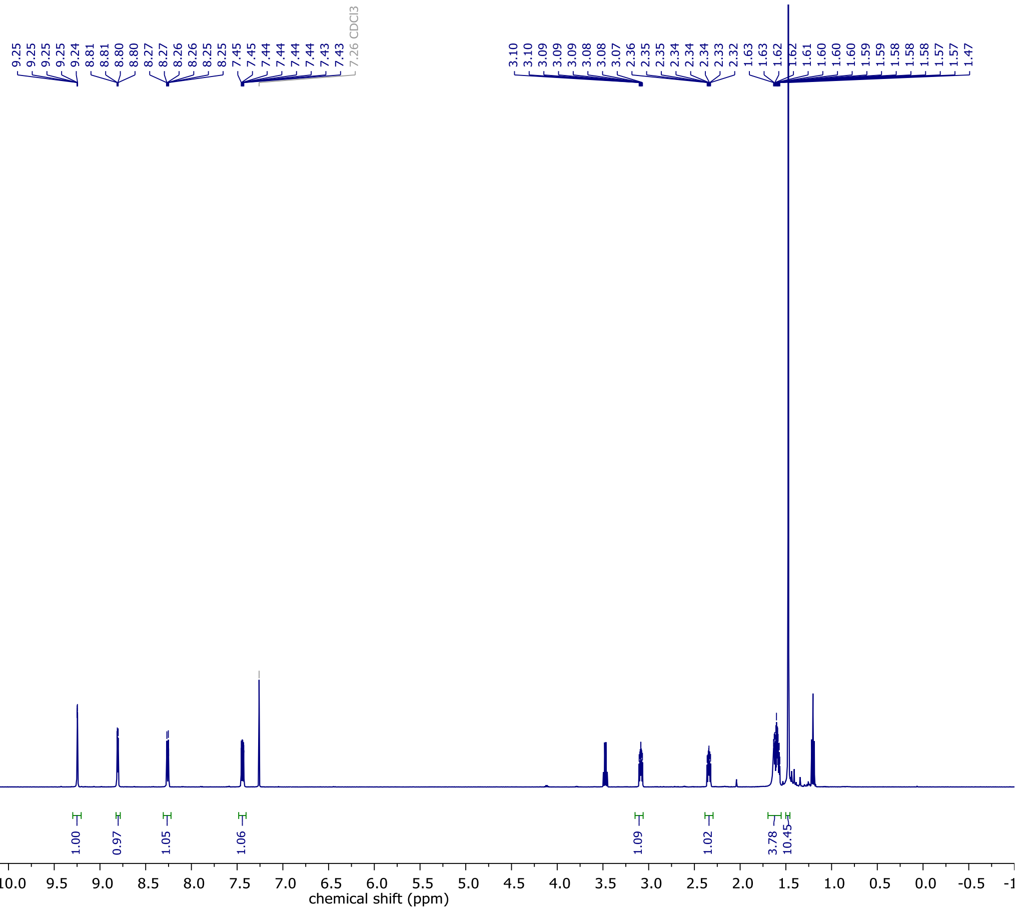
9.25 9.25 9.25 9.25 9.24 8.81 8.81 8.80 8.80 8.27 8.26 8.26 8.25 7.45 7.45 7.44 7.44 7.44 7.43 7.43 7.26 CDCl3
3.10 3.10 3.09 3.09 3.09 3.08 3.08 3.07 2.36 2.35 2.35 2.34 2.34 2.33 2.32 1.63 1.63 1.62 1.62 1.61 1.60 1.60 1.59 1.59 1.58 1.58 1.58 1.57 1.57 1.47
1.00
0.97
1.05
1.06
1.09
1.02
3.78
10.45
chemical shift (ppm)

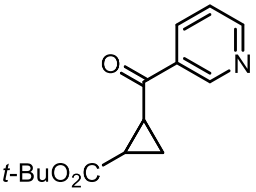

1b

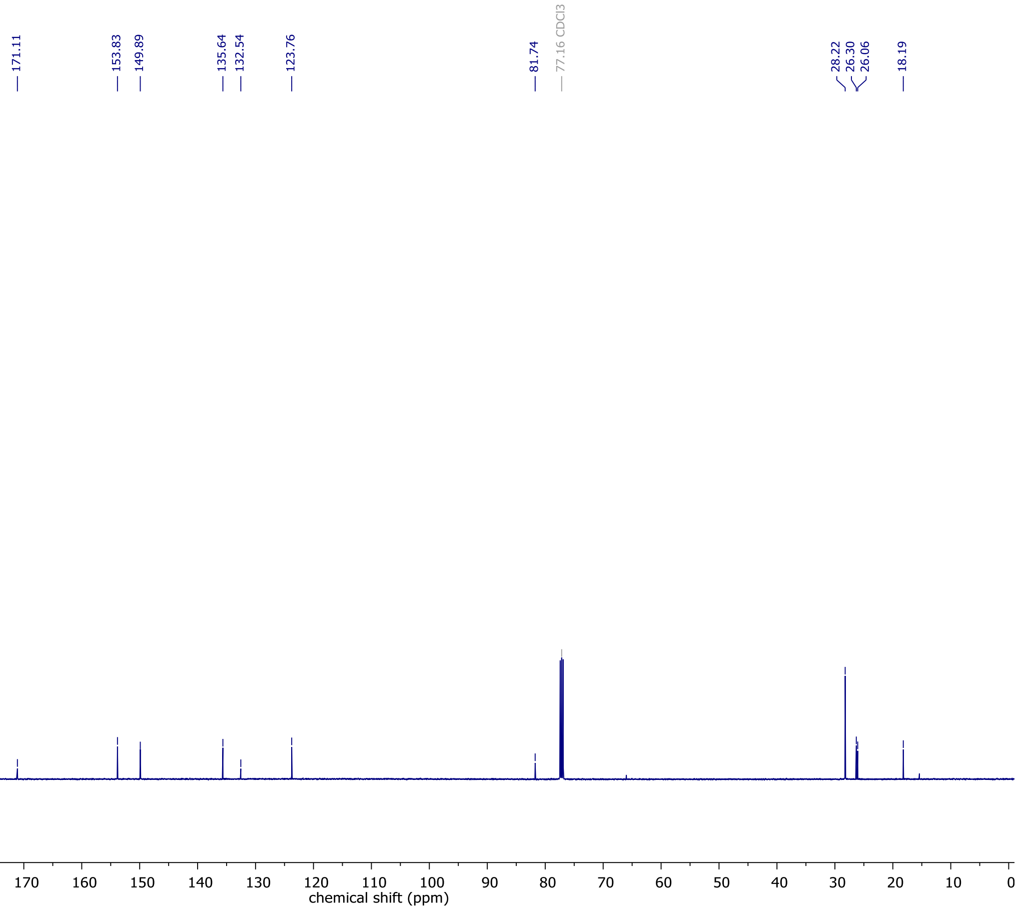
196.48
171.11
153.83
149.89
135.64
132.54
123.76
81.74
77.16 CDCl3
28.22
26.30
26.06
18.19
chemical shift (ppm)

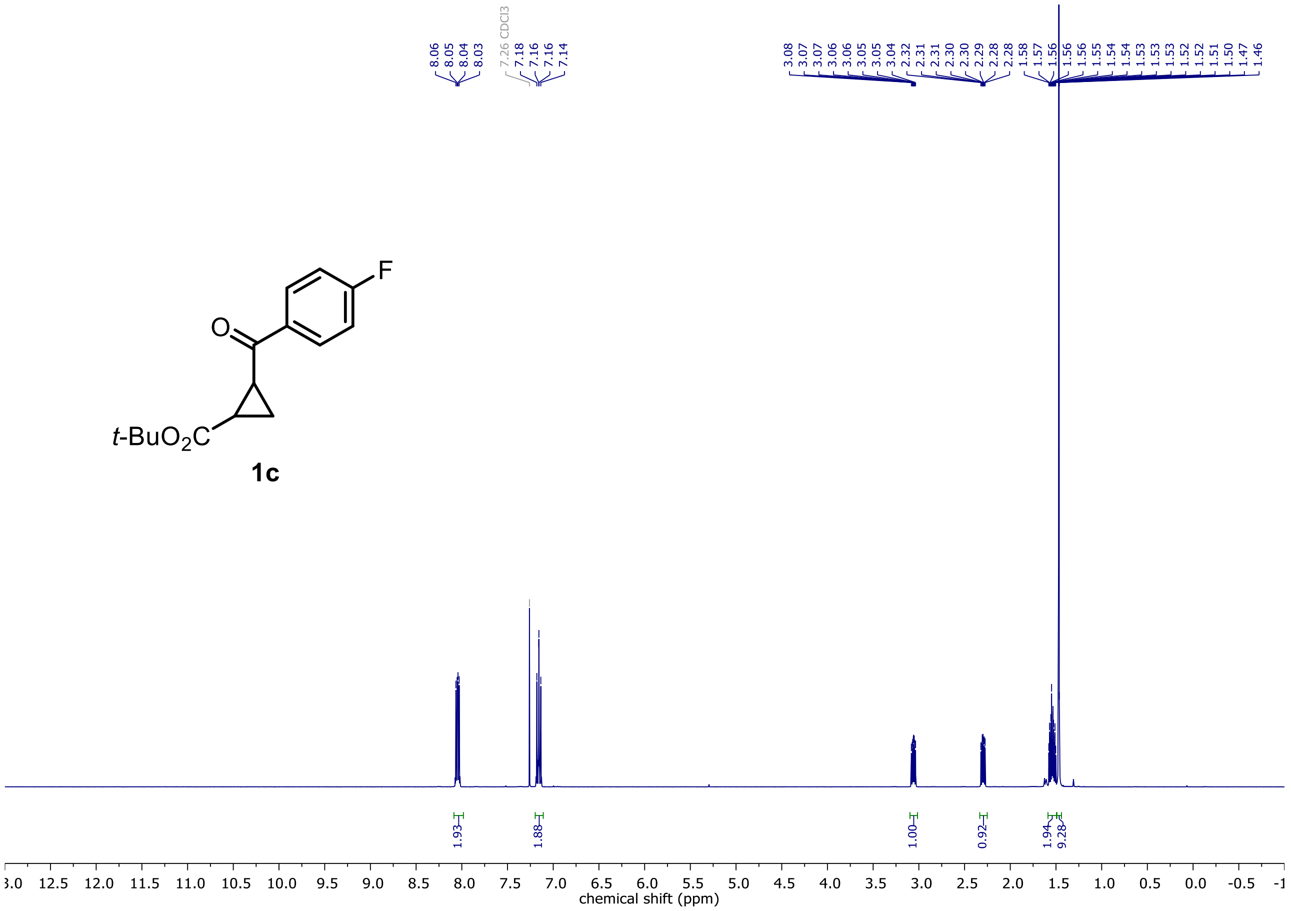

8.06 8.05 8.04 8.03
7.26 CDCl3
7.18 7.16 7.16 7.14
3.08 3.07 3.07 3.06 3.06 3.05 3.05 3.04 2.32 2.31 2.31 2.30 2.30 2.29 2.28 2.28 1.58 1.57 1.56 1.56 1.56 1.55 1.54 1.54 1.53 1.53 1.53 1.52 1.52 1.51 1.50 1.47 1.46
F
O
t-BuO$_2$C
1c
1.93
1.88
1.00
0.92
1.94
9.28
chemical shift (ppm)

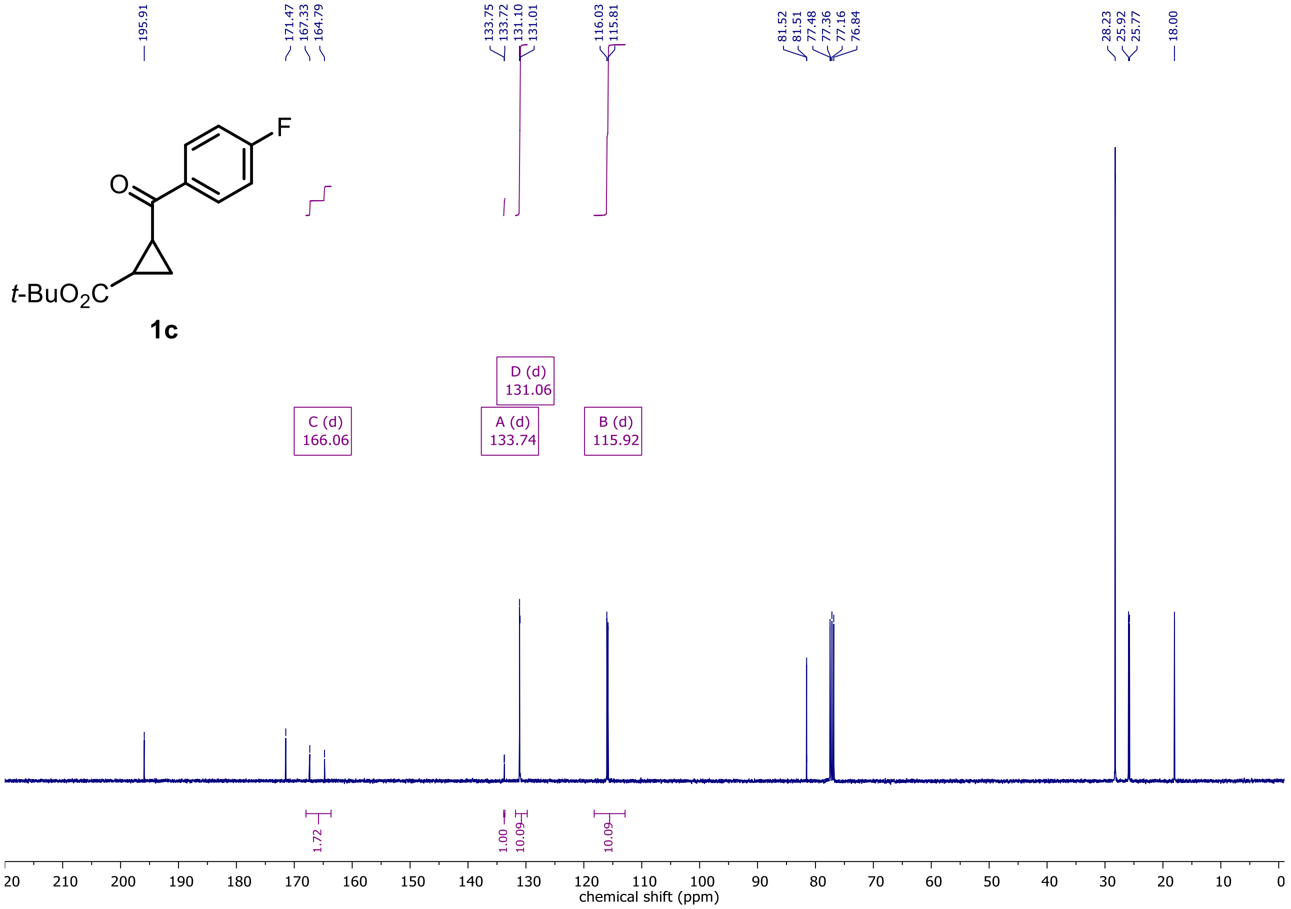

195.91
171.47 167.33 164.79
133.75 133.72 131.10 131.01
116.03 115.81
81.52 81.51 77.48 77.36 77.16 76.84
28.23 25.92 25.77
18.00
F
O
t-BuO$_2$C
1c
D (d) 131.06
C (d) 166.06
A (d) 133.74
B (d) 115.92
1.72
1.00
10.09
10.09
chemical shift (ppm)

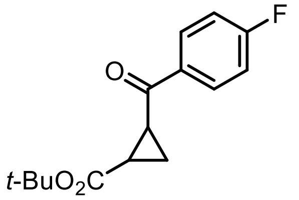
F
O
t-BuO2C

1c

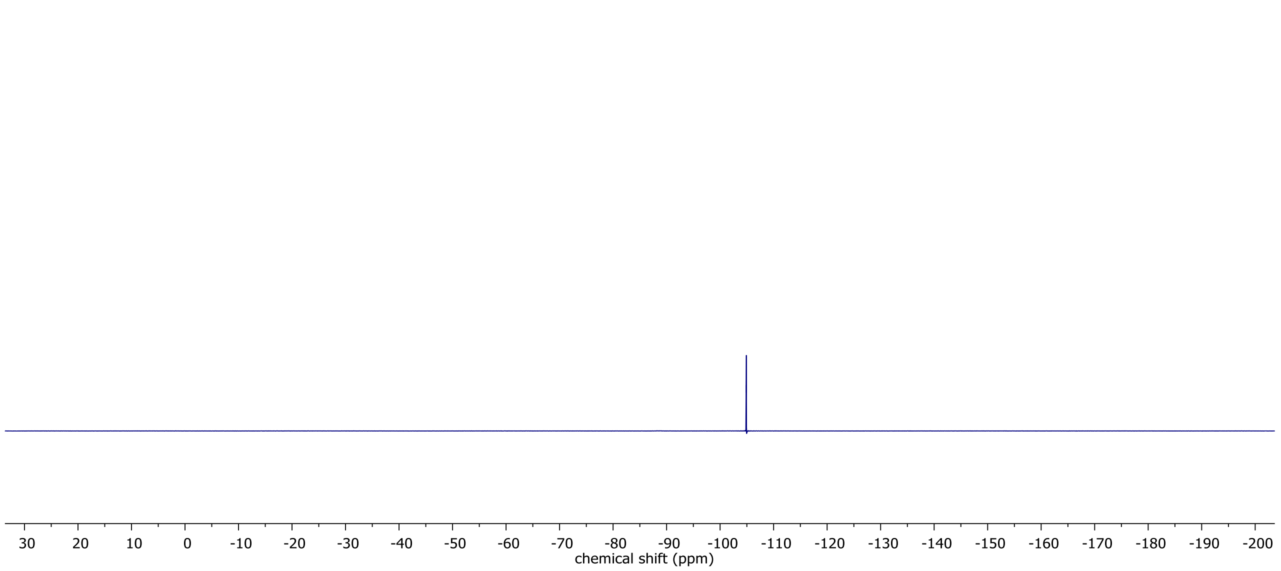
-104.89
-104.92
-104.93
30 20 10 0 -10 -20 -30 -40 -50 -60 -70 -80 -90 -100 -110 -120 -130 -140 -150 -160 -170 -180 -190 -200
chemical shift (ppm)

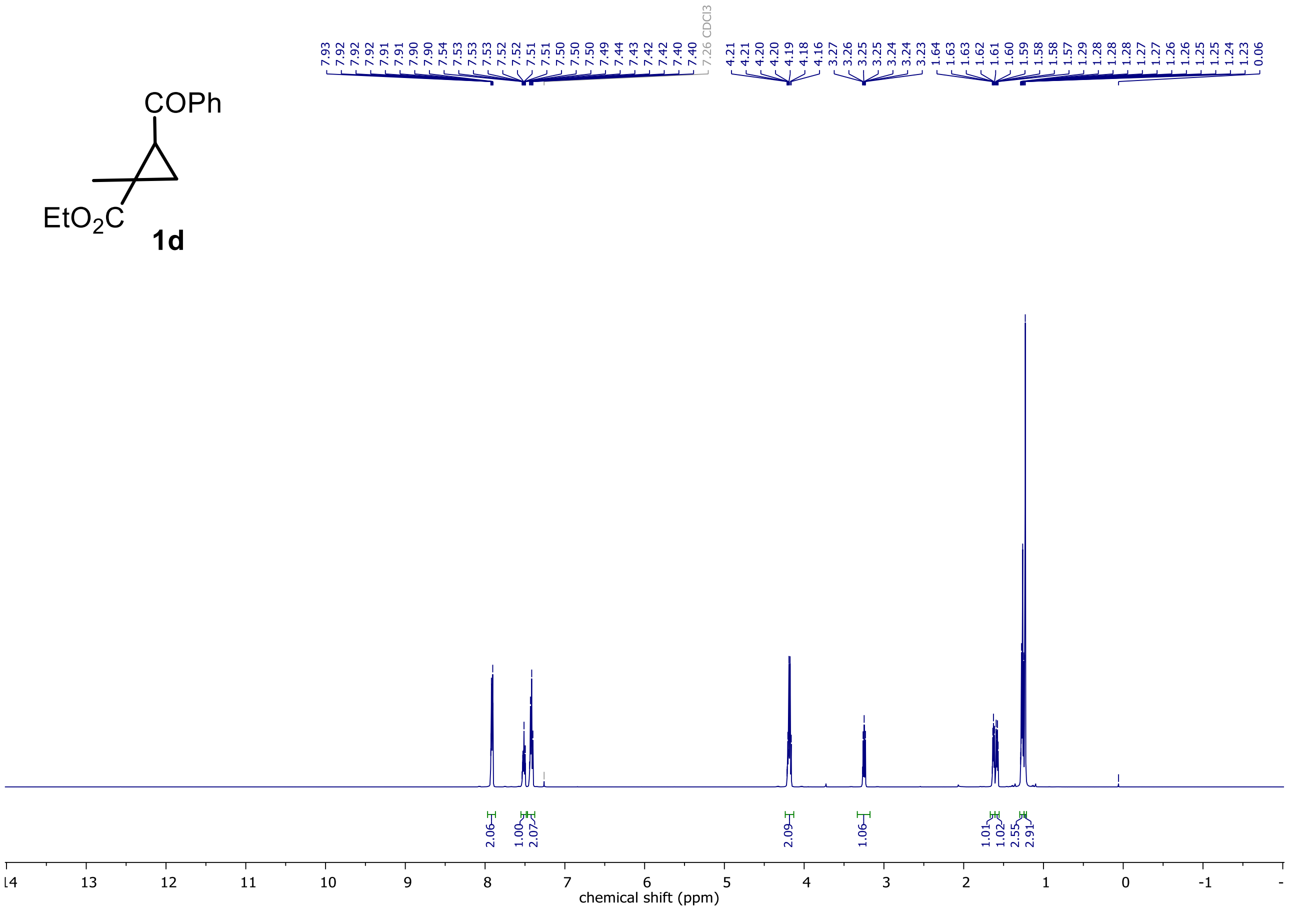
COPh
EtO2C
1d
7.93 7.92 7.92 7.92 7.91 7.91 7.90 7.90 7.54 7.53 7.53 7.53 7.52 7.52 7.51 7.51 7.50 7.50 7.49 7.44 7.43 7.42 7.42 7.40 7.40
7.26 CDCl3
4.21 4.21 4.20 4.20 4.19 4.18 4.16 3.27 3.26 3.25 3.25 3.24 3.24 3.23 1.64 1.63 1.63 1.62 1.61 1.60 1.59 1.58 1.58 1.57 1.29 1.28 1.28 1.28 1.27 1.27 1.26 1.26 1.25 1.25 1.24 1.23 0.06
2.06
1.00
2.07
2.09
1.06
1.01
1.02
2.55
2.91
14 13 12 11 10 9 8 7 6 5 4 3 2 1 0 -1
chemical shift (ppm)

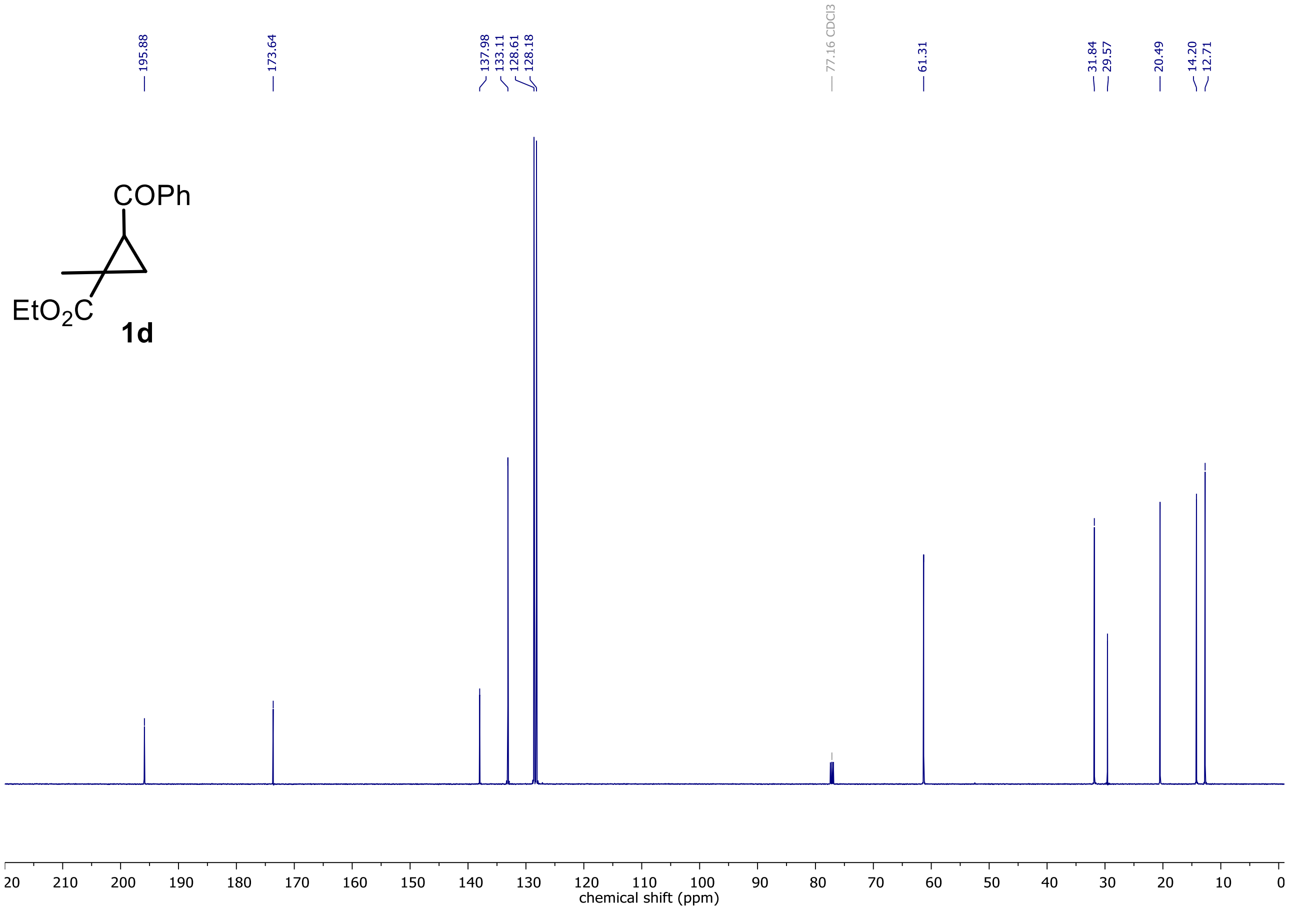
195.88
173.64
137.98 133.11 128.61 128.18
77.16 CDCl3
61.31
31.84 29.57
20.49
14.20 12.71
COPh
EtO2C
1d
220 210 200 190 180 170 160 150 140 130 120 110 100 90 80 70 60 50 40 30 20 10 0
chemical shift (ppm)

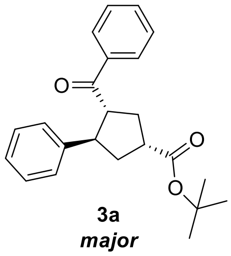

3a
major

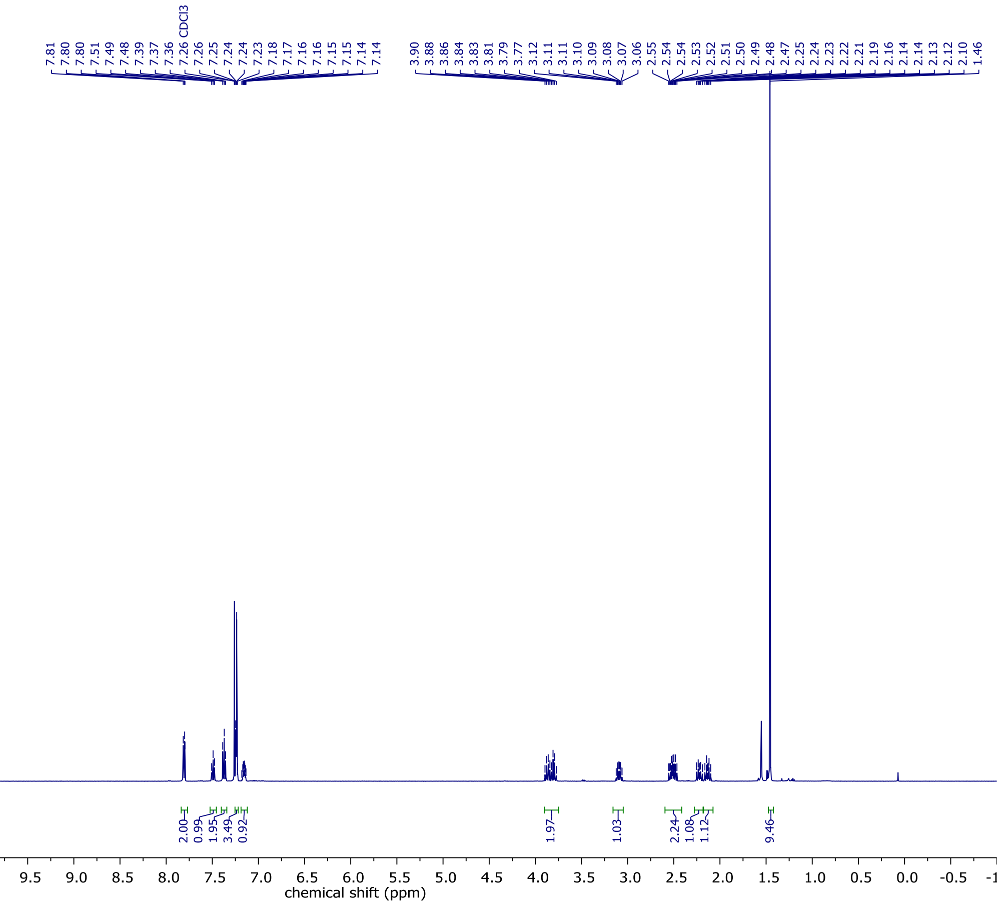

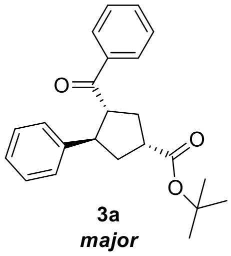

3a
major

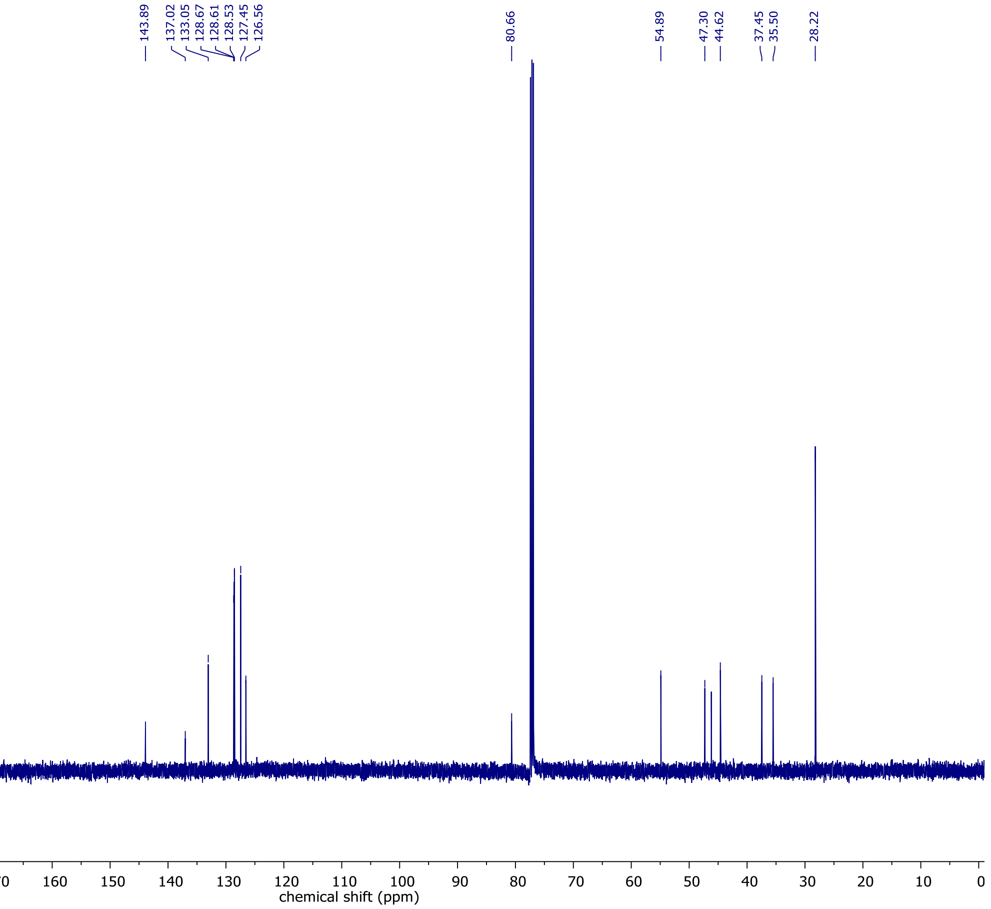

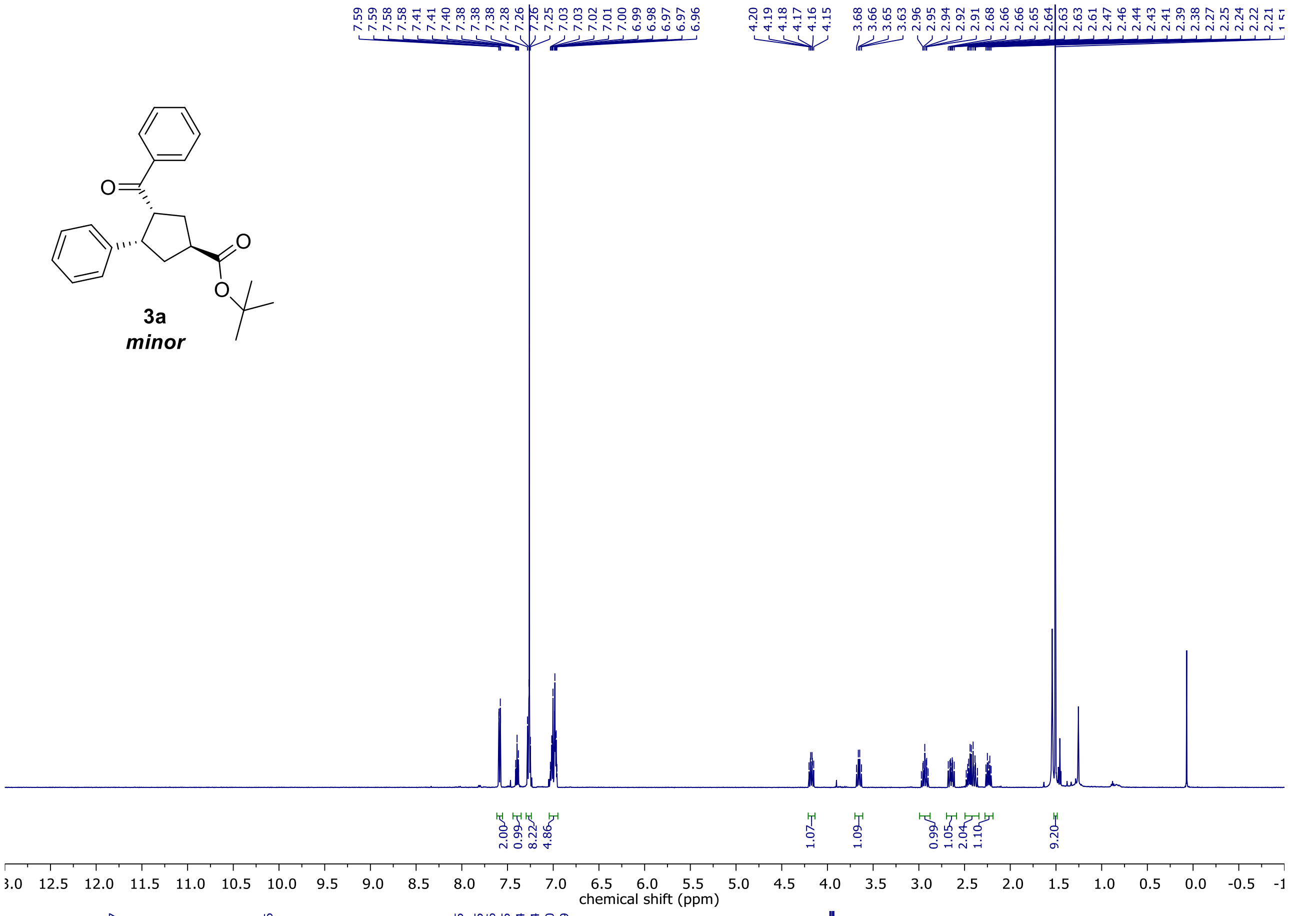
3a
minor
7.59 7.59 7.58 7.58 7.41 7.40 7.38 7.38 7.38 7.28 7.26 7.26 7.25 7.03 7.03 7.02 7.01 7.00 6.99 6.98 6.97 6.97 6.96
4.20 4.19 4.18 4.17 4.16 4.15
3.68 3.66 3.65 3.63
2.96 2.95 2.94 2.92 2.91 2.68 2.66 2.66 2.65 2.64 2.63 2.63 2.61 2.47 2.46 2.44 2.43 2.41 2.39 2.38 2.27 2.25 2.24 2.22 2.21
2.00 0.99 8.22 4.86 1.07 1.09 0.99 1.05 2.04 1.10 9.20
chemical shift (ppm)

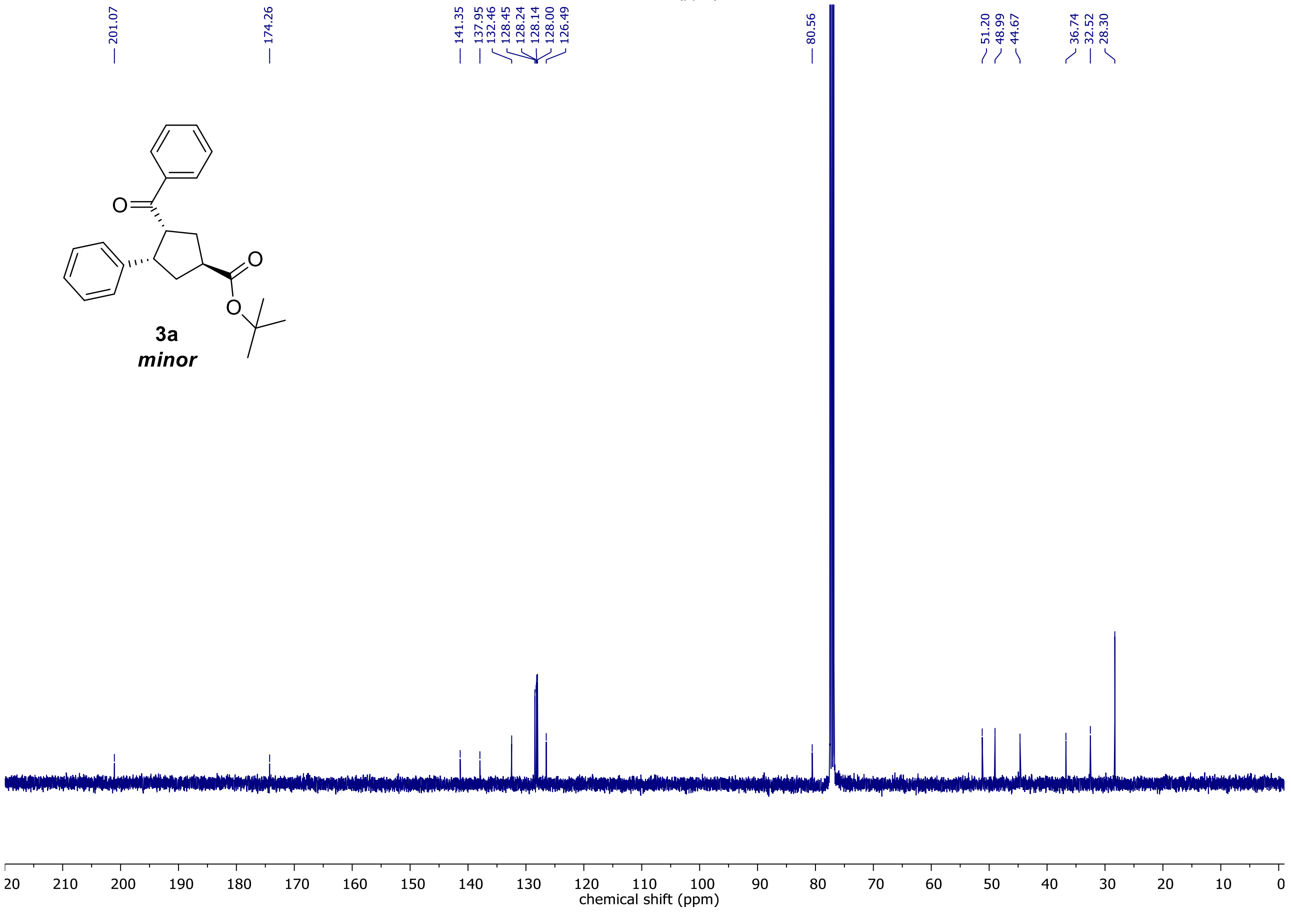
3a
minor
201.07
174.26
141.35 137.95 132.46 128.45 128.24 128.14 128.00 126.49
80.56
51.20 48.99 44.67
36.74 32.52 28.30
chemical shift (ppm)

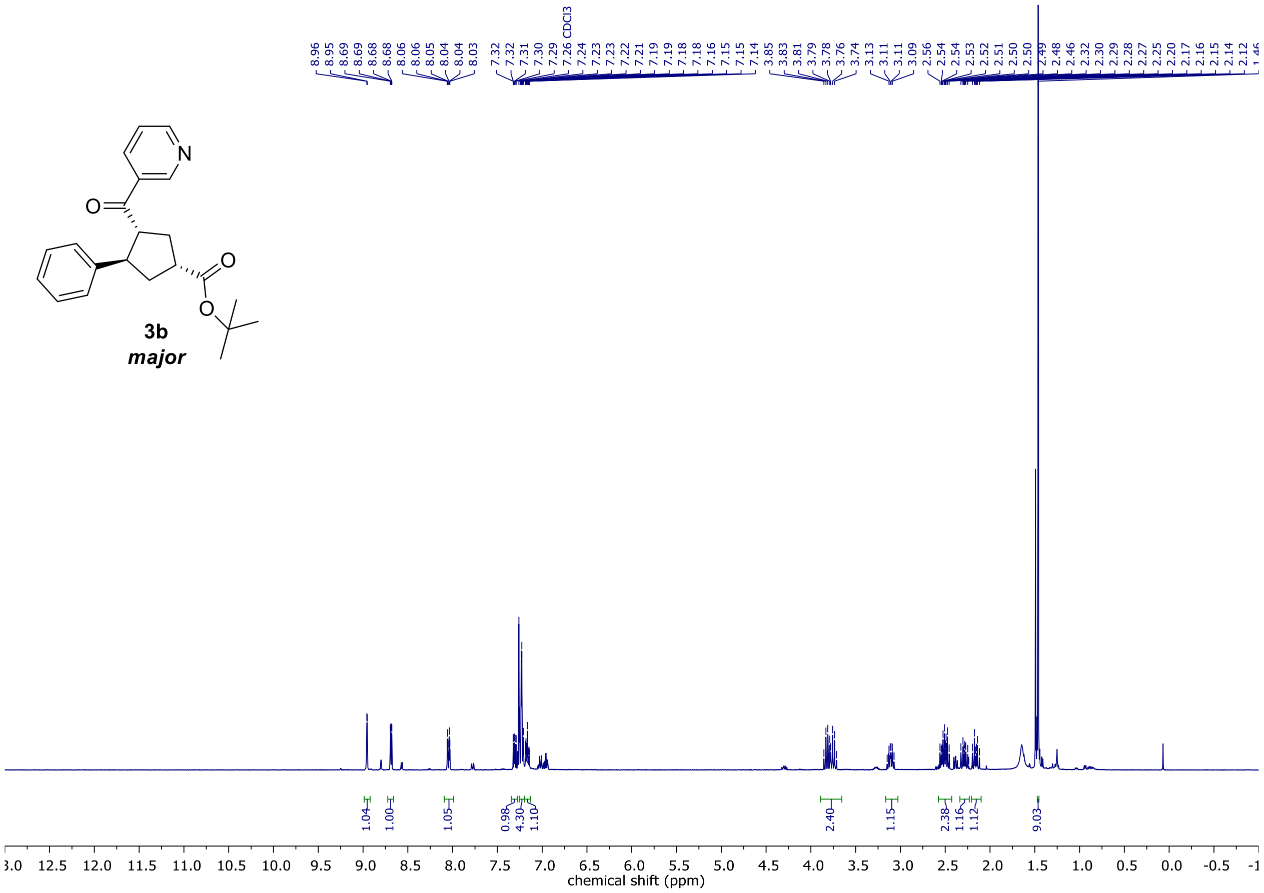

3b
major
8.96 8.95 8.69 8.69 8.68 8.68 8.06 8.06 8.05 8.04 8.04 8.03 7.32 7.32 7.31 7.30 7.29 7.26 CDCl3 7.24 7.23 7.23 7.22 7.21 7.19 7.19 7.18 7.16 7.15 7.15 7.14 3.85 3.83 3.81 3.79 3.78 3.76 3.74 3.13 3.11 3.11 3.09 2.56 2.54 2.54 2.53 2.52 2.51 2.50 2.50 2.49 2.48 2.46 2.32 2.30 2.29 2.28 2.27 2.25 2.20 2.17 2.16 2.15 2.14 2.12
1.04 1.00 1.05 0.98 4.30 1.10 2.40 1.15 2.38 1.16 1.12 9.03
12.5 12.0 11.5 11.0 10.5 10.0 9.5 9.0 8.5 8.0 7.5 7.0 6.5 6.0 5.5 5.0 4.5 4.0 3.5 3.0 2.5 2.0 1.5 1.0 0.5 0.0 -0.5
chemical shift (ppm)

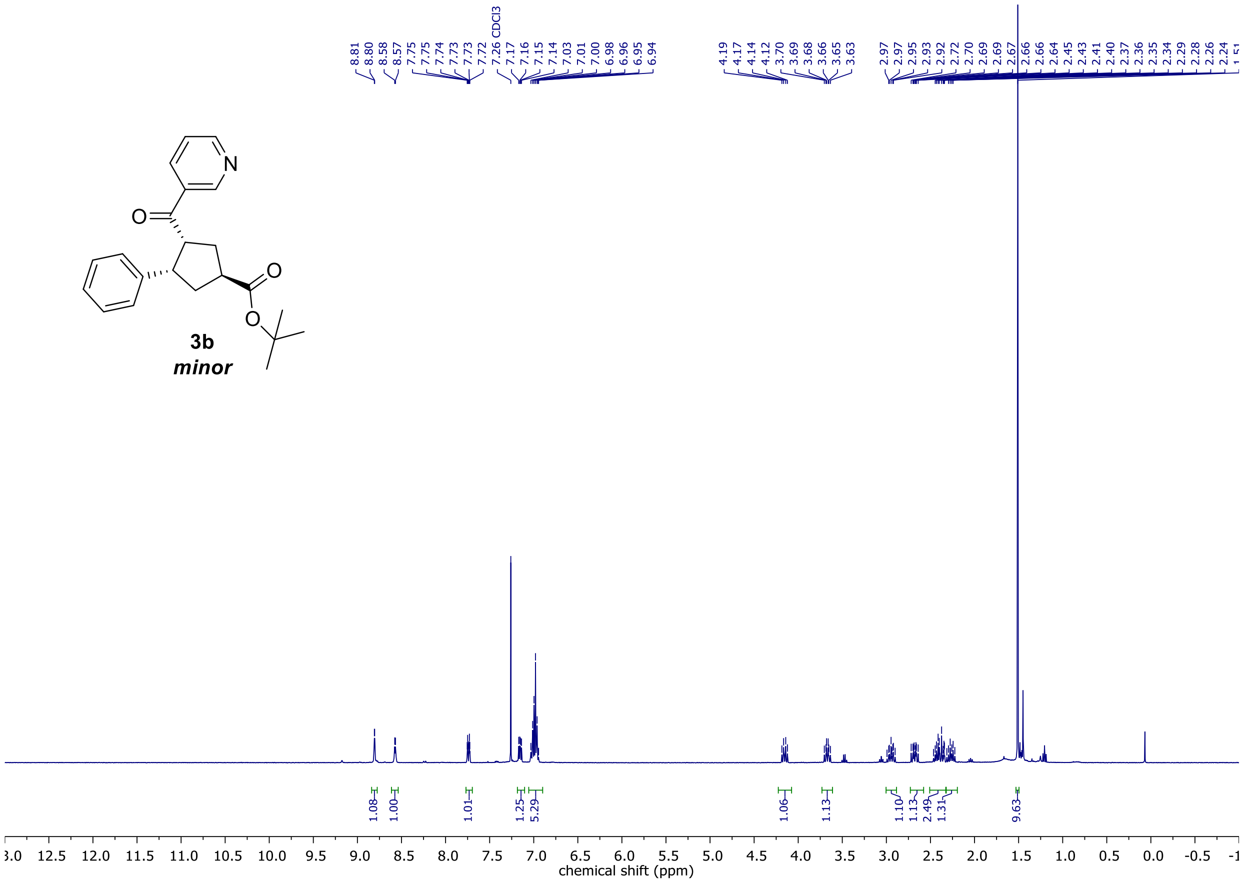
8.81 8.80 8.58 8.57 7.75 7.75 7.74 7.73 7.73 7.72 7.26 CDCl3 7.17 7.16 7.15 7.14 7.03 7.01 7.00 6.98 6.96 6.95 6.94
4.19 4.17 4.14 4.12 3.70 3.69 3.68 3.66 3.65 3.63
2.97 2.97 2.95 2.93 2.92 2.72 2.70 2.69 2.69 2.67 2.66 2.66 2.64 2.45 2.43 2.41 2.40 2.37 2.36 2.35 2.34 2.29 2.28 2.26 2.24
3b
minor
1.08 1.00 1.01 1.25 5.29 1.06 1.13 1.10 1.13 2.49 1.31 9.63
chemical shift (ppm)

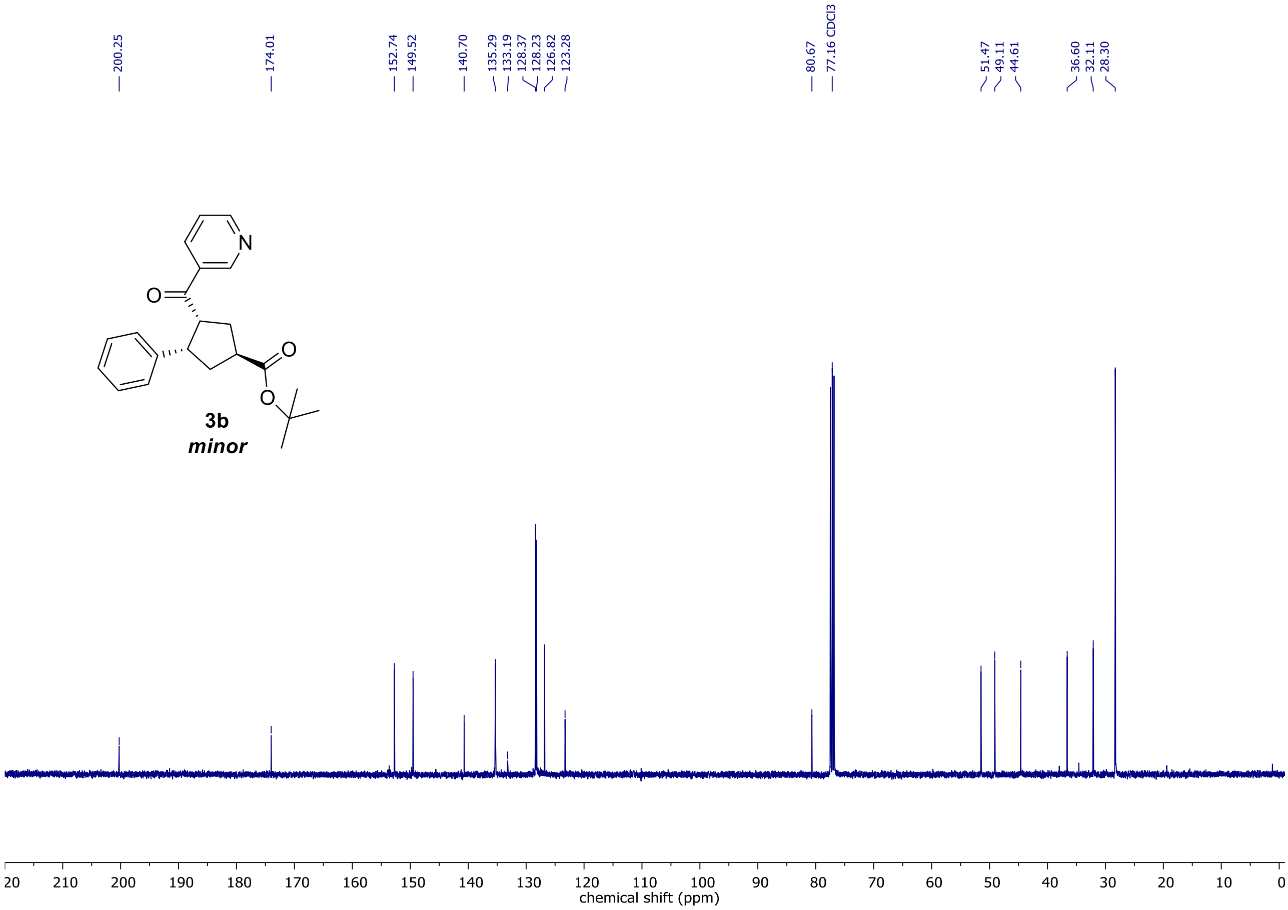
200.25 174.01 152.74 149.52 140.70 135.29 133.19 128.37 128.23 126.82 123.28 80.67 77.16 CDCl3 51.47 49.11 44.61 36.60 32.11 28.30
3b
minor
chemical shift (ppm)

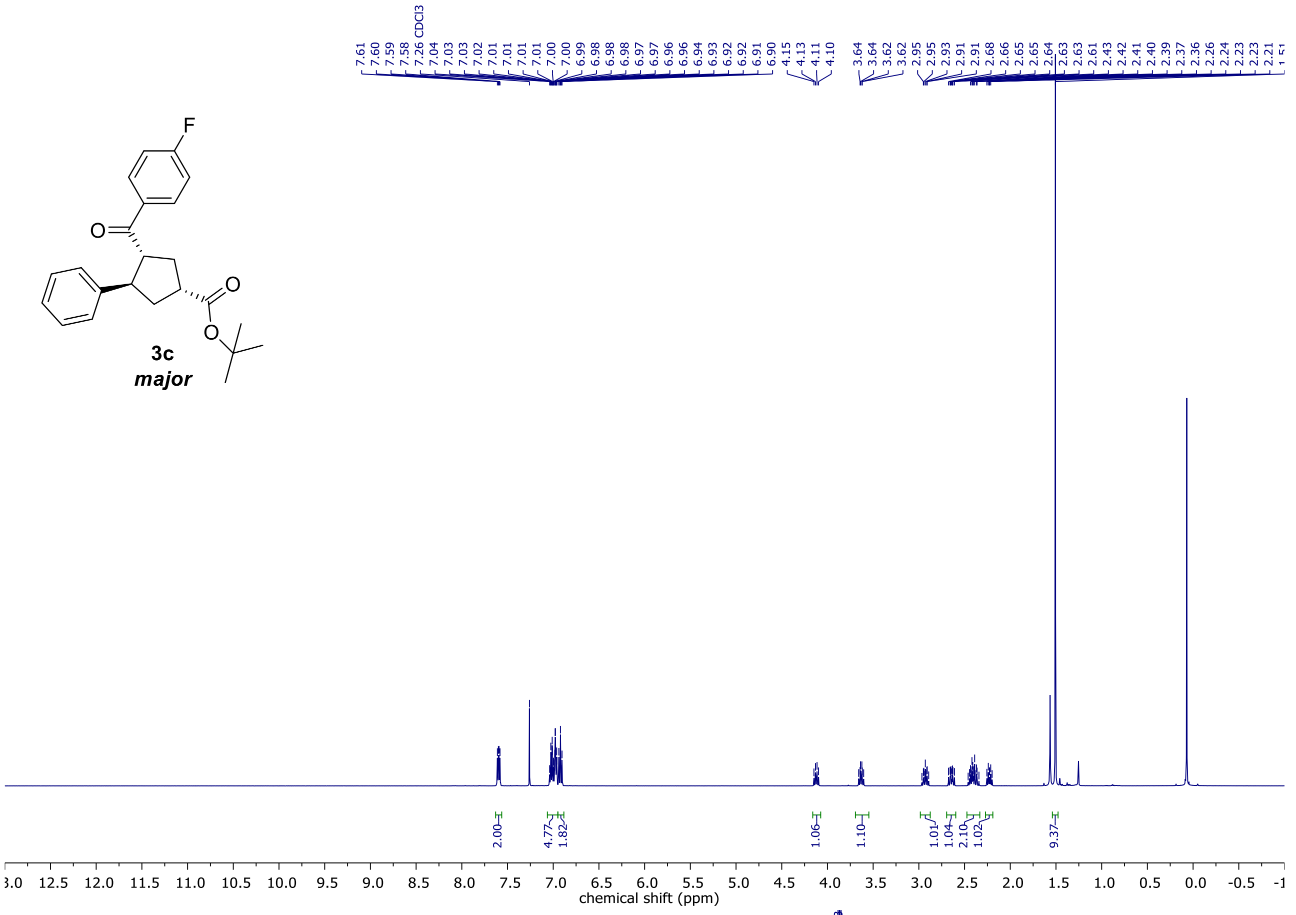

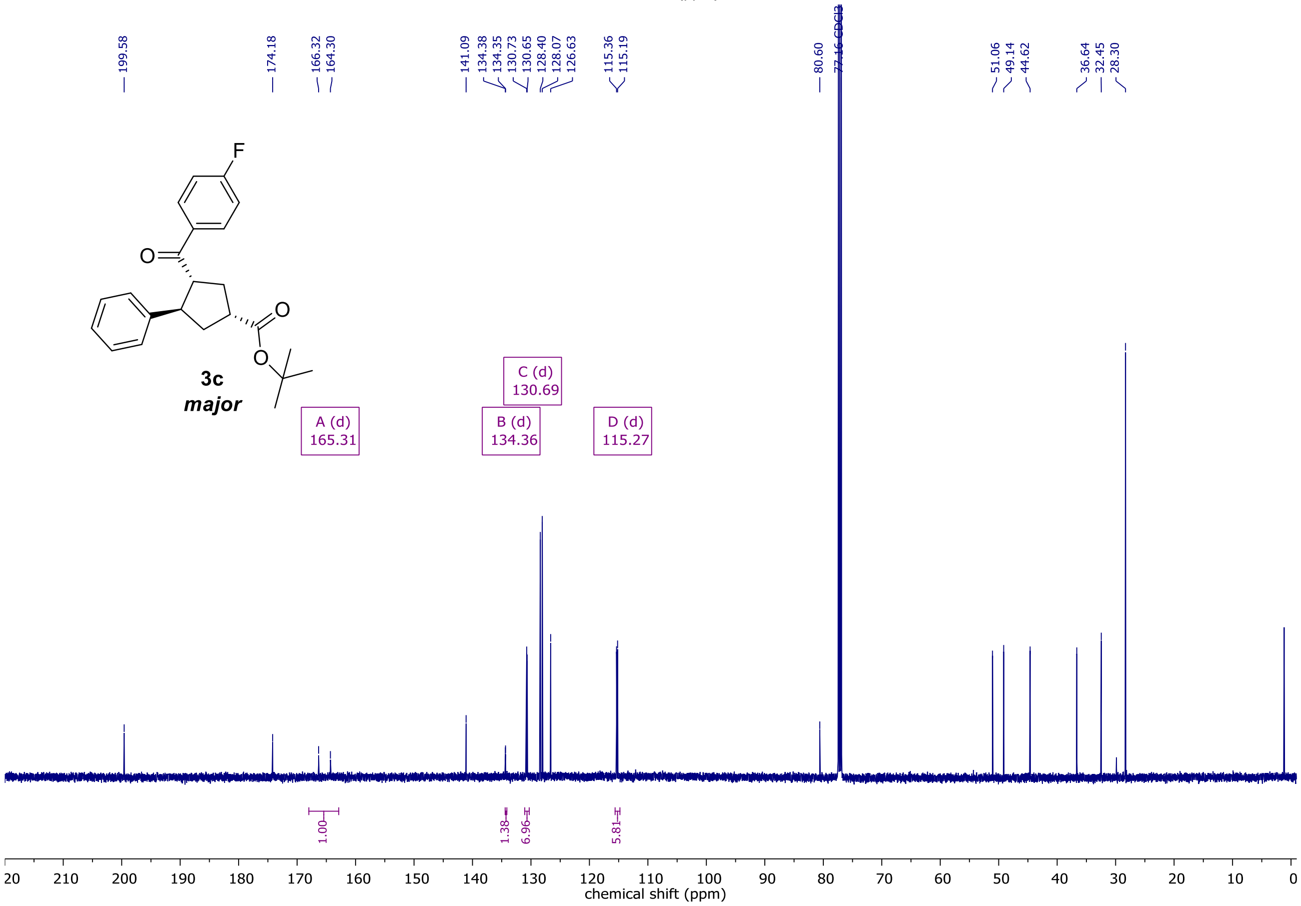

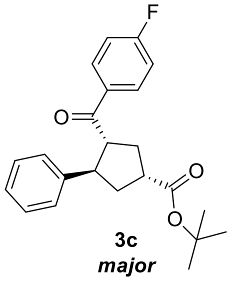

3c
major

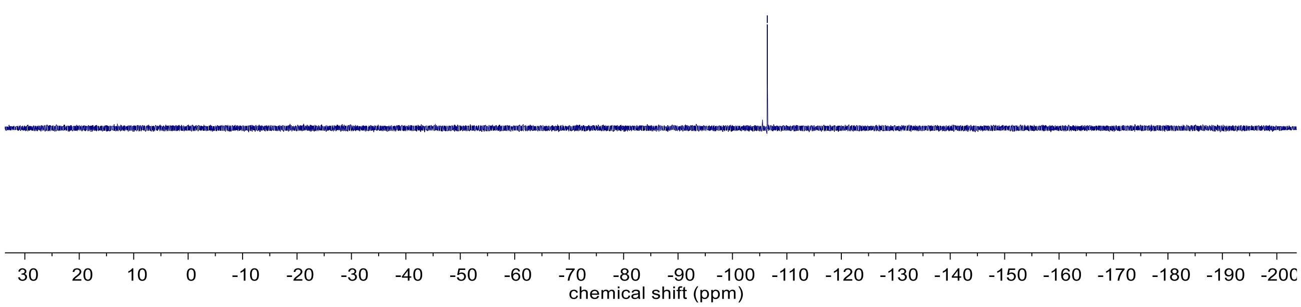

-106.40
30 20 10 0 -10 -20 -30 -40 -50 -60 -70 -80 -90 -100 -110 -120 -130 -140 -150 -160 -170 -180 -190 -200
chemical shift (ppm)

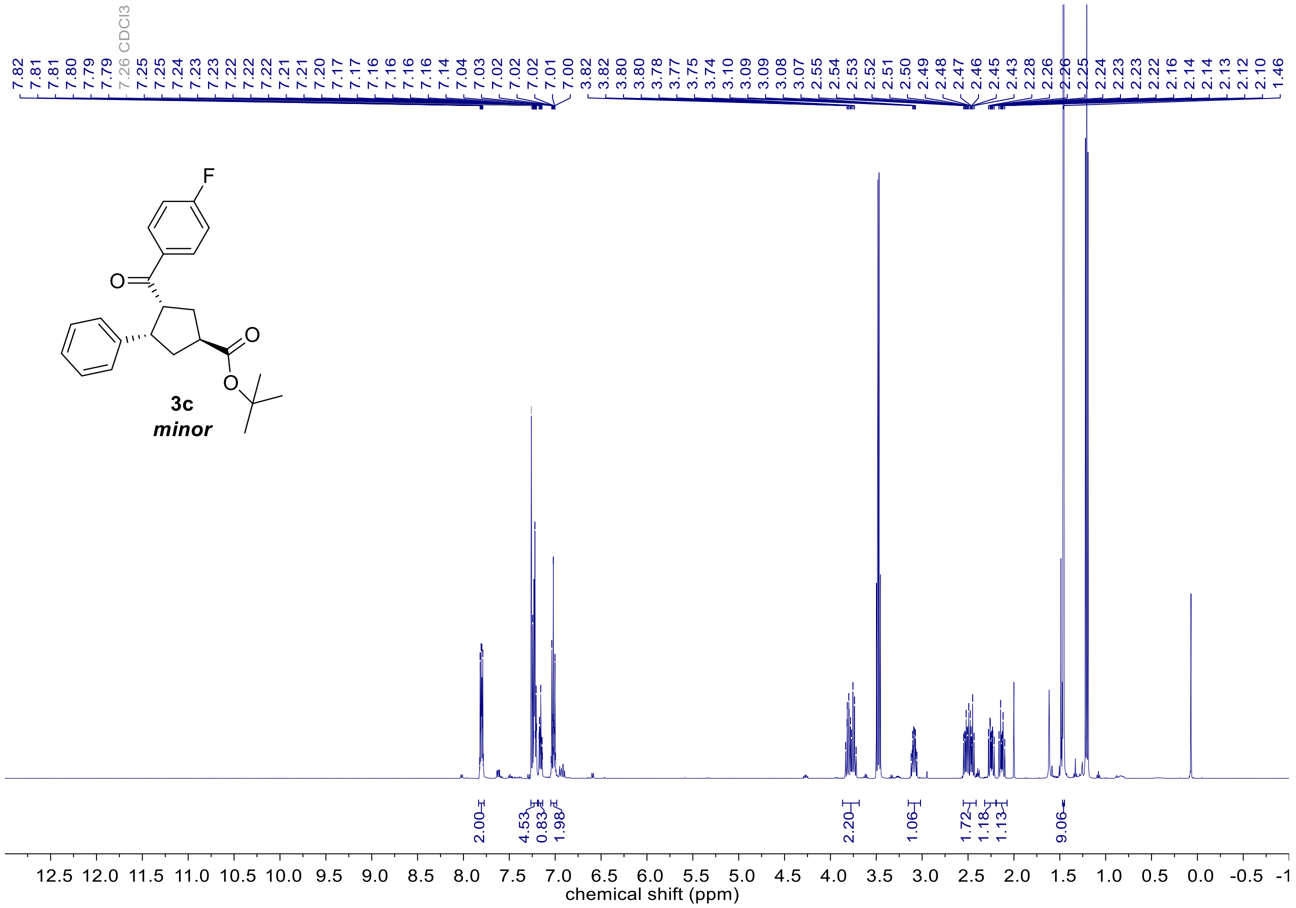
7.82 7.81 7.81 7.80 7.79 7.26 CDCl3 7.25 7.25 7.24 7.23 7.23 7.22 7.22 7.22 7.21 7.21 7.20 7.17 7.16 7.16 7.16 7.14 7.04 7.03 7.02 7.02 7.02 7.01 7.00 3.82 3.82 3.80 3.78 3.77 3.75 3.74 3.10 3.09 3.09 3.08 3.07 2.55 2.54 2.53 2.52 2.51 2.50 2.49 2.48 2.47 2.46 2.45 2.43 2.28 2.26 2.26 2.25 2.24 2.23 2.23 2.22 2.16 2.14 2.13 2.12 2.10 1.46
F
O
3c
minor
2.00 4.53 0.83 1.98 2.20 1.06 1.72 1.18 1.13 9.06
chemical shift (ppm)

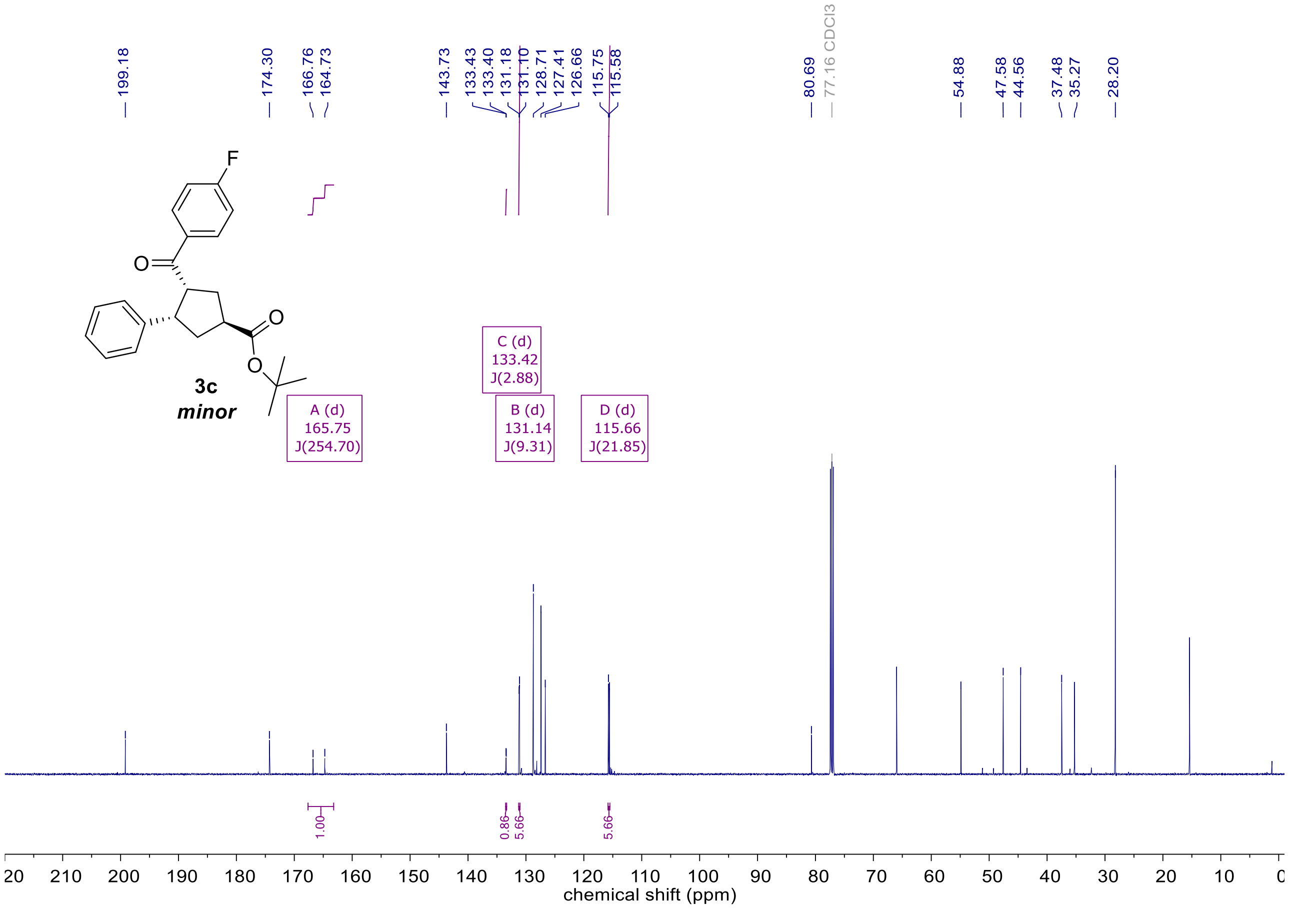
199.18 174.30 166.76 164.73 143.73 133.43 133.40 131.18 131.10 128.71 127.41 126.66 115.75 115.58 80.69 77.16 CDCl3 54.88 47.58 44.56 37.48 35.27 28.20
F
O
3c
minor
C (d) 133.42 J(2.88)
A (d) 165.75 J(254.70)
B (d) 131.14 J(9.31)
D (d) 115.66 J(21.85)
1.00 0.86 5.66 5.66
chemical shift (ppm)

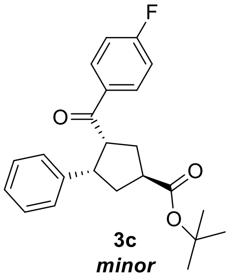

3c
minor

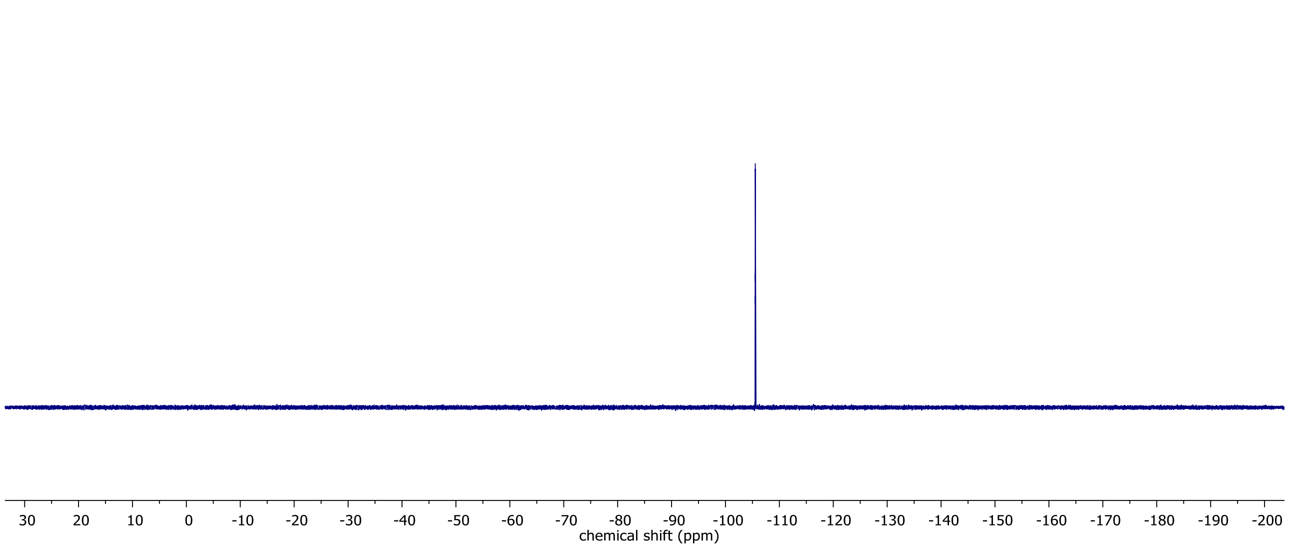

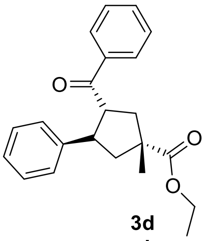

3d
major

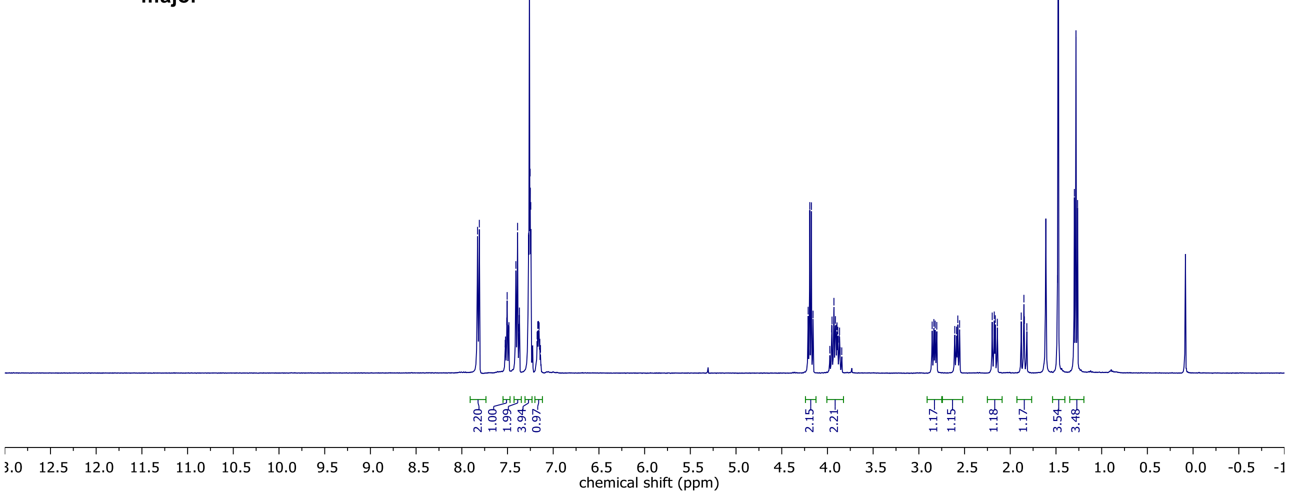

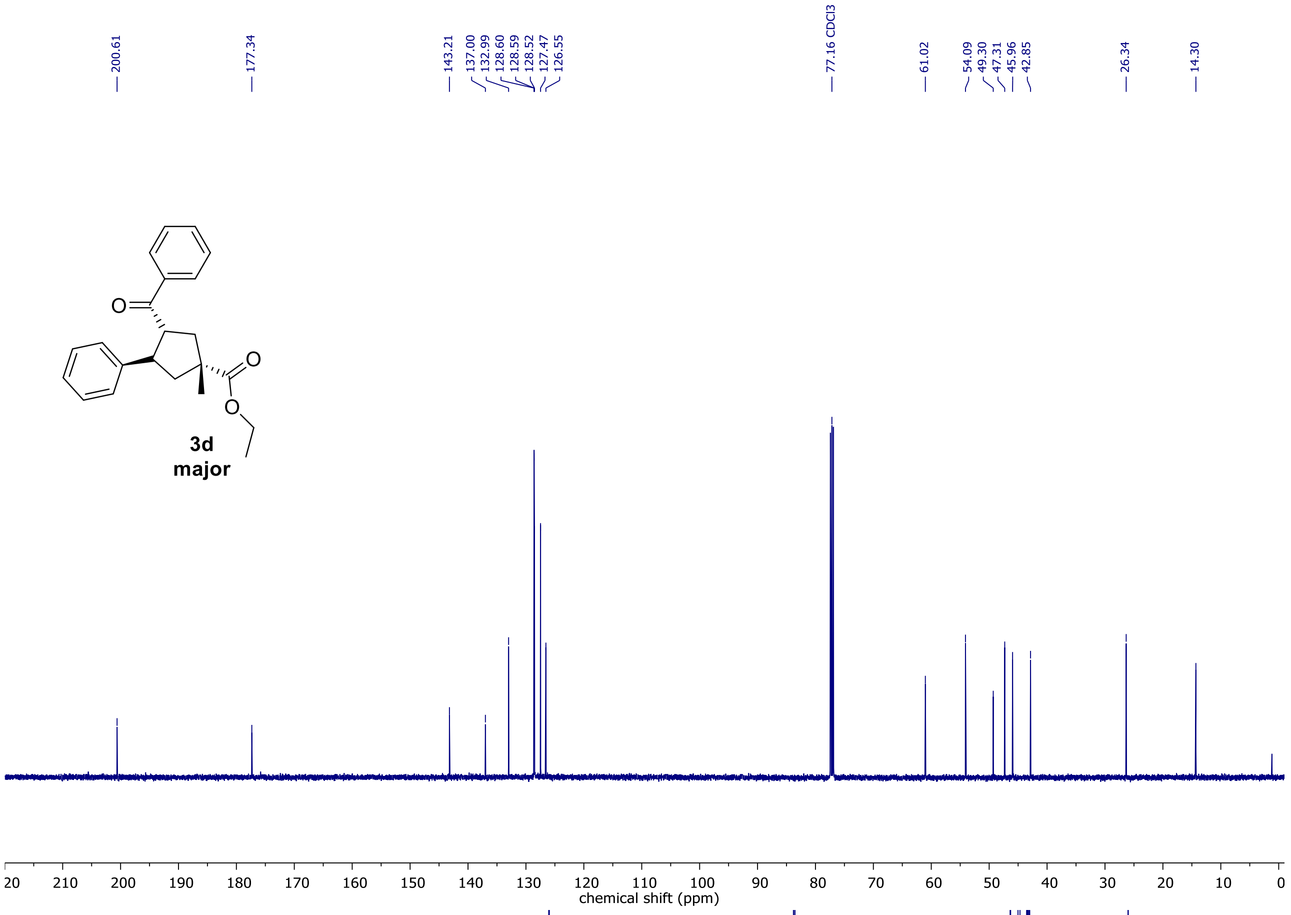
200.61
177.34
143.21
137.00
132.99
128.60
128.59
128.52
127.47
126.55
77.16 CDCl3
61.02
54.09
49.30
47.31
45.96
42.85
26.34
14.30
O
O
O
3d
major
220 210 200 190 180 170 160 150 140 130 120 110 100 90 80 70 60 50 40 30 20 10 0
chemical shift (ppm)

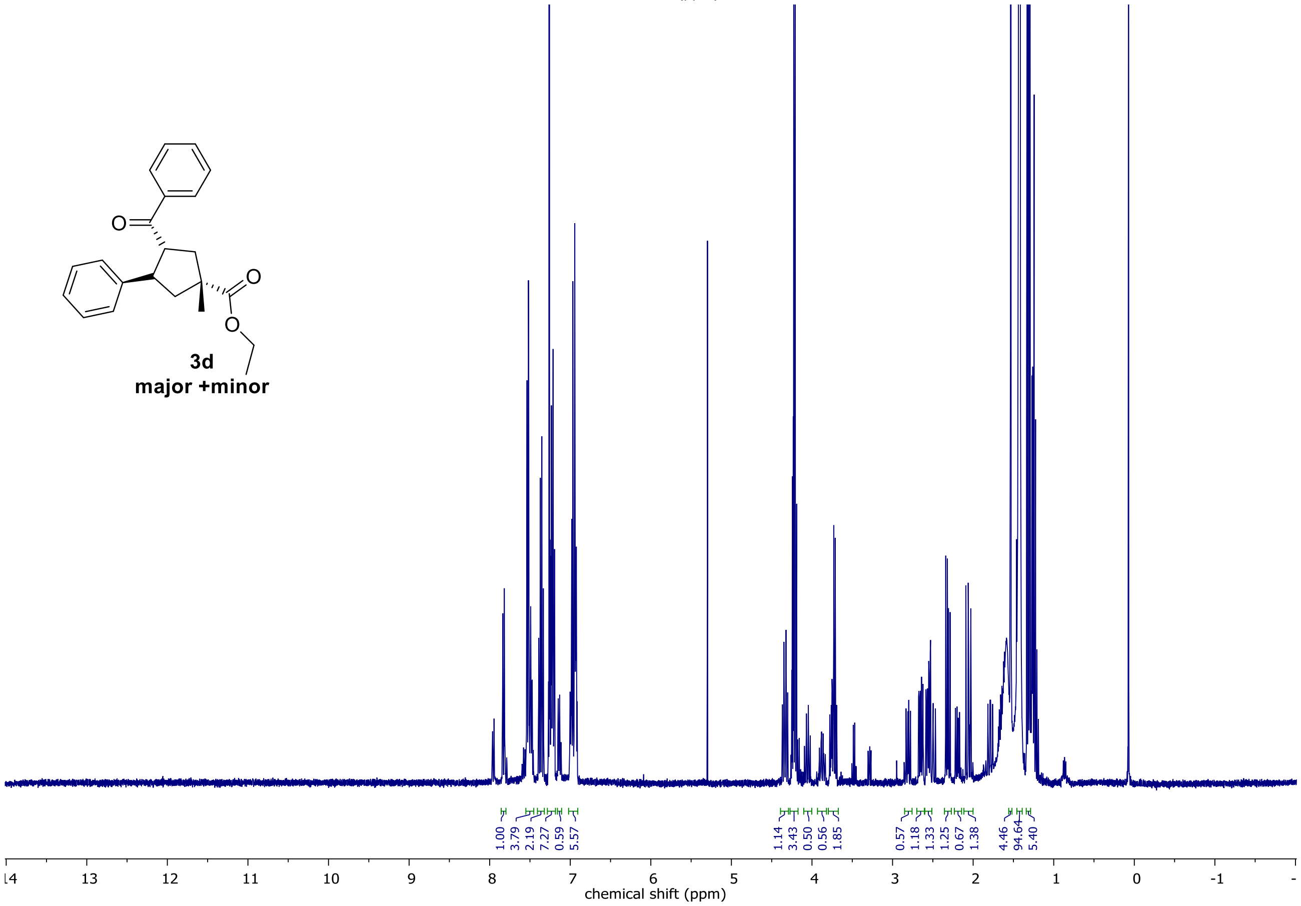
O
O
O
3d
major +minor
1.00
3.79
2.19
7.27
0.59
5.57
1.14
3.43
0.50
0.56
1.85
0.57
1.18
1.33
1.25
0.67
1.38
4.46
94.64
5.40
14 13 12 11 10 9 8 7 6 5 4 3 2 1 0 -1
chemical shift (ppm)

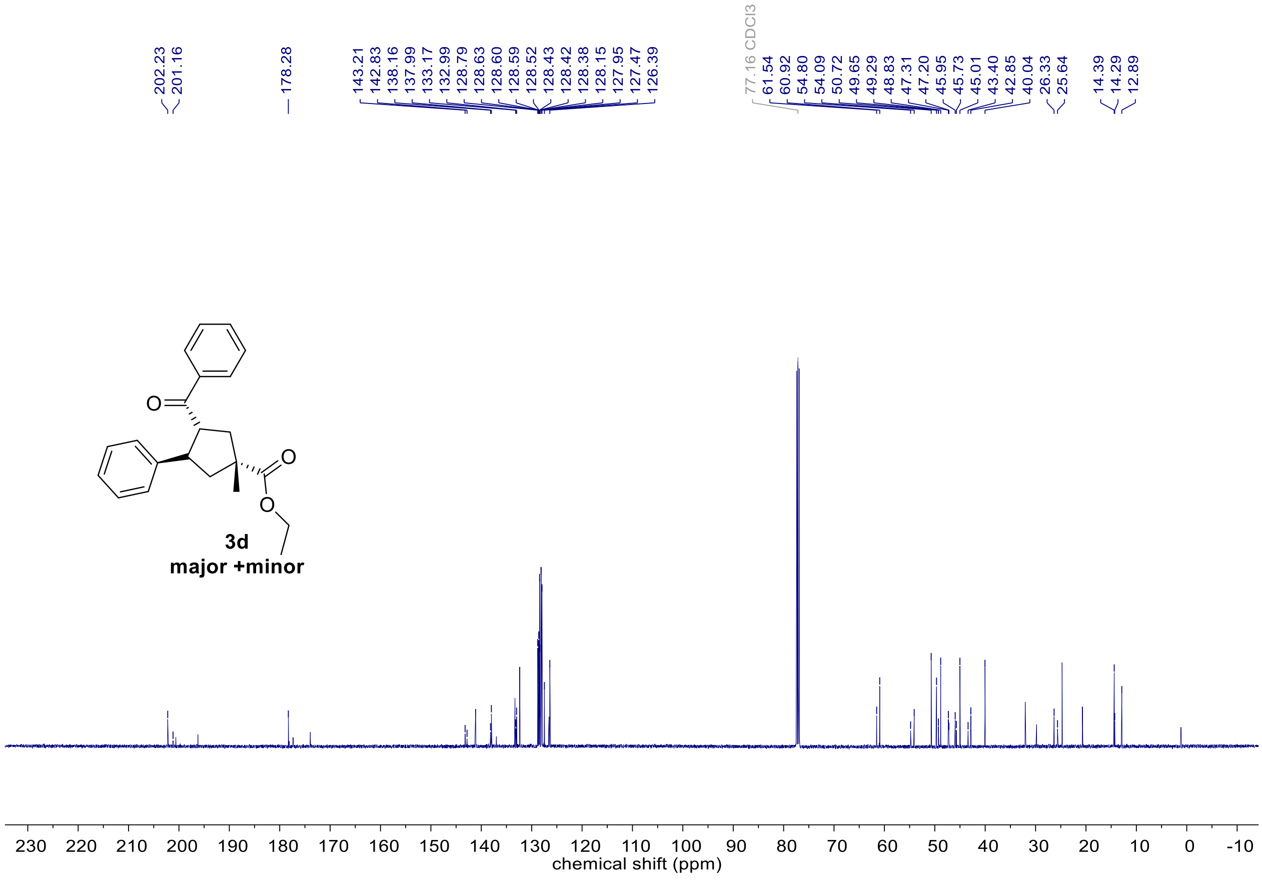
202.23
201.16
178.28
143.21
142.83
138.16
137.99
133.17
132.99
128.79
128.63
128.60
128.59
128.52
128.43
128.42
128.38
128.15
127.95
127.47
126.39
77.16 CDCl3
61.54
60.92
54.80
54.09
50.72
49.65
49.29
48.83
47.31
47.20
45.95
45.73
45.01
43.40
42.85
40.04
26.33
25.64
14.39
14.29
12.89
O
O
O
3d
major +minor
230 220 210 200 190 180 170 160 150 140 130 120 110 100 90 80 70 60 50 40 30 20 10 0 -10
chemical shift (ppm)

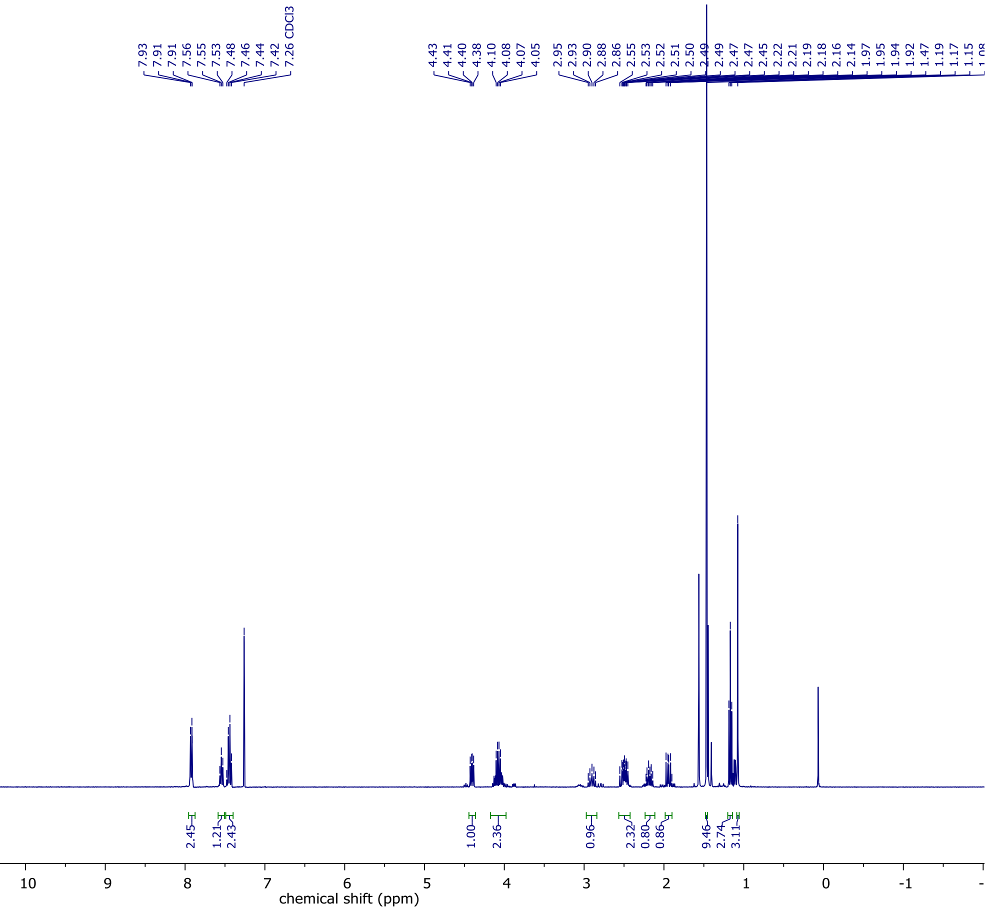

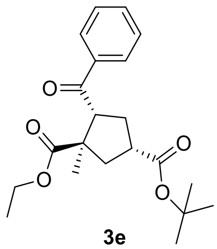

3e
major

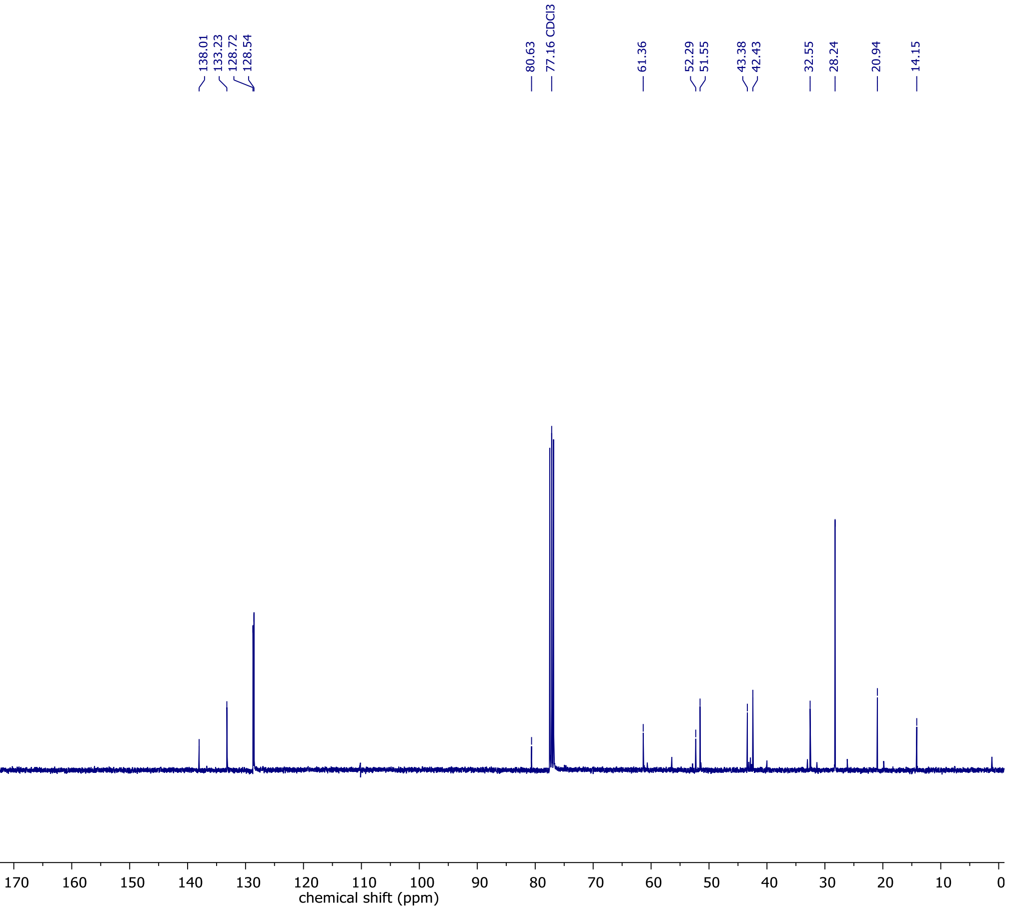

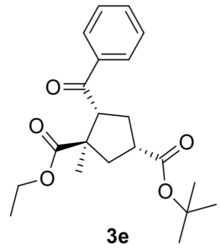

3e
major

3e
minor

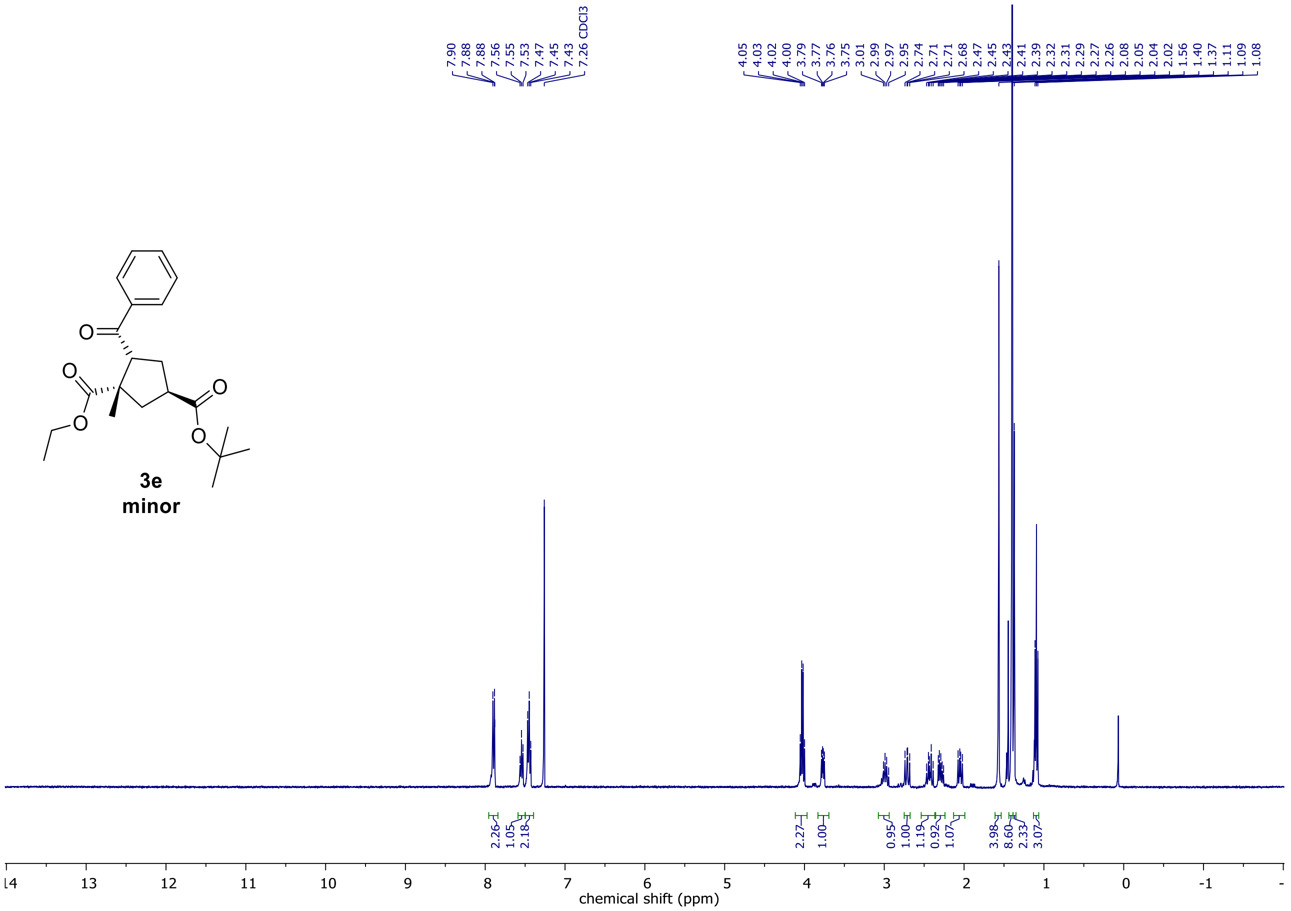

3e
minor

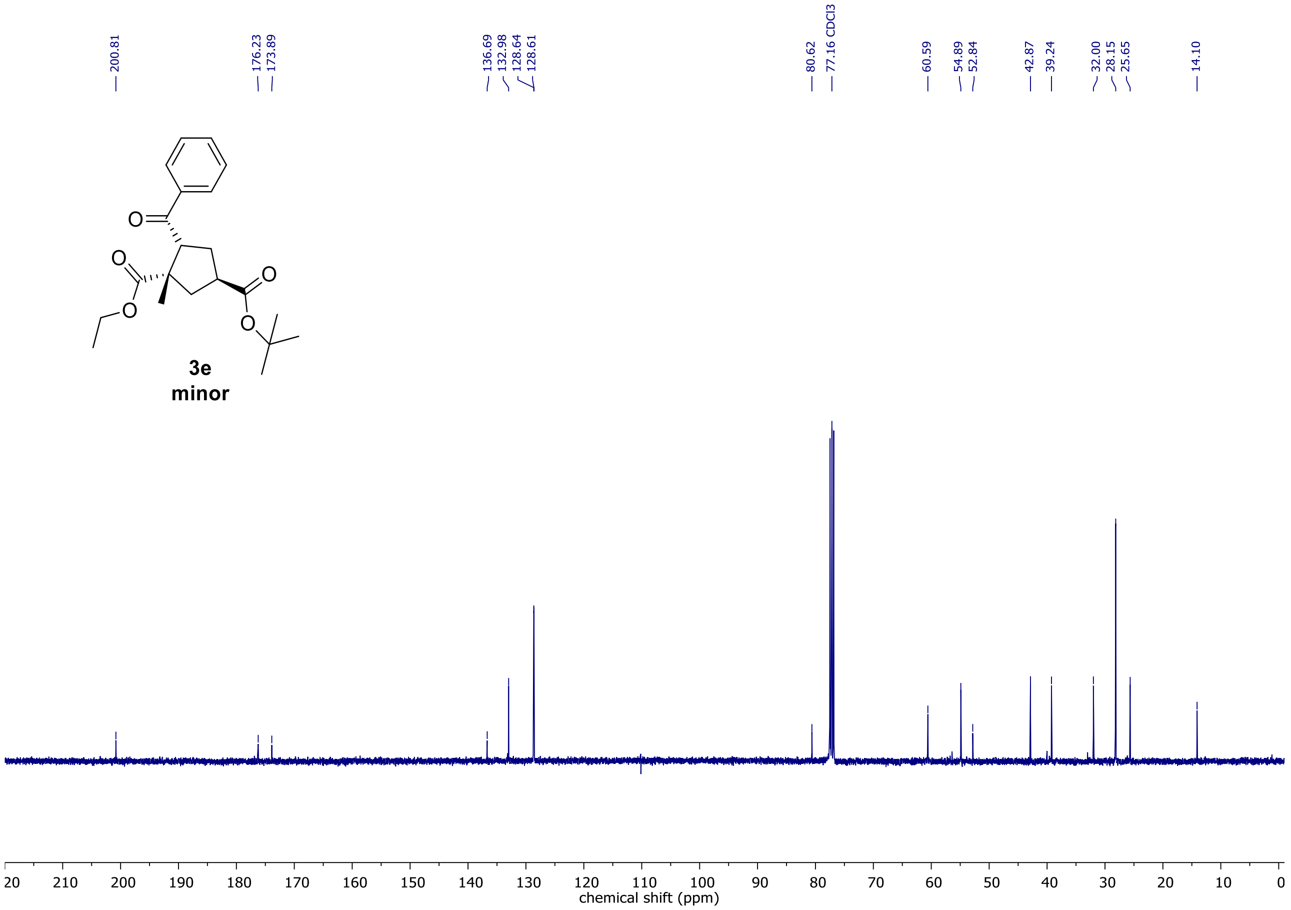

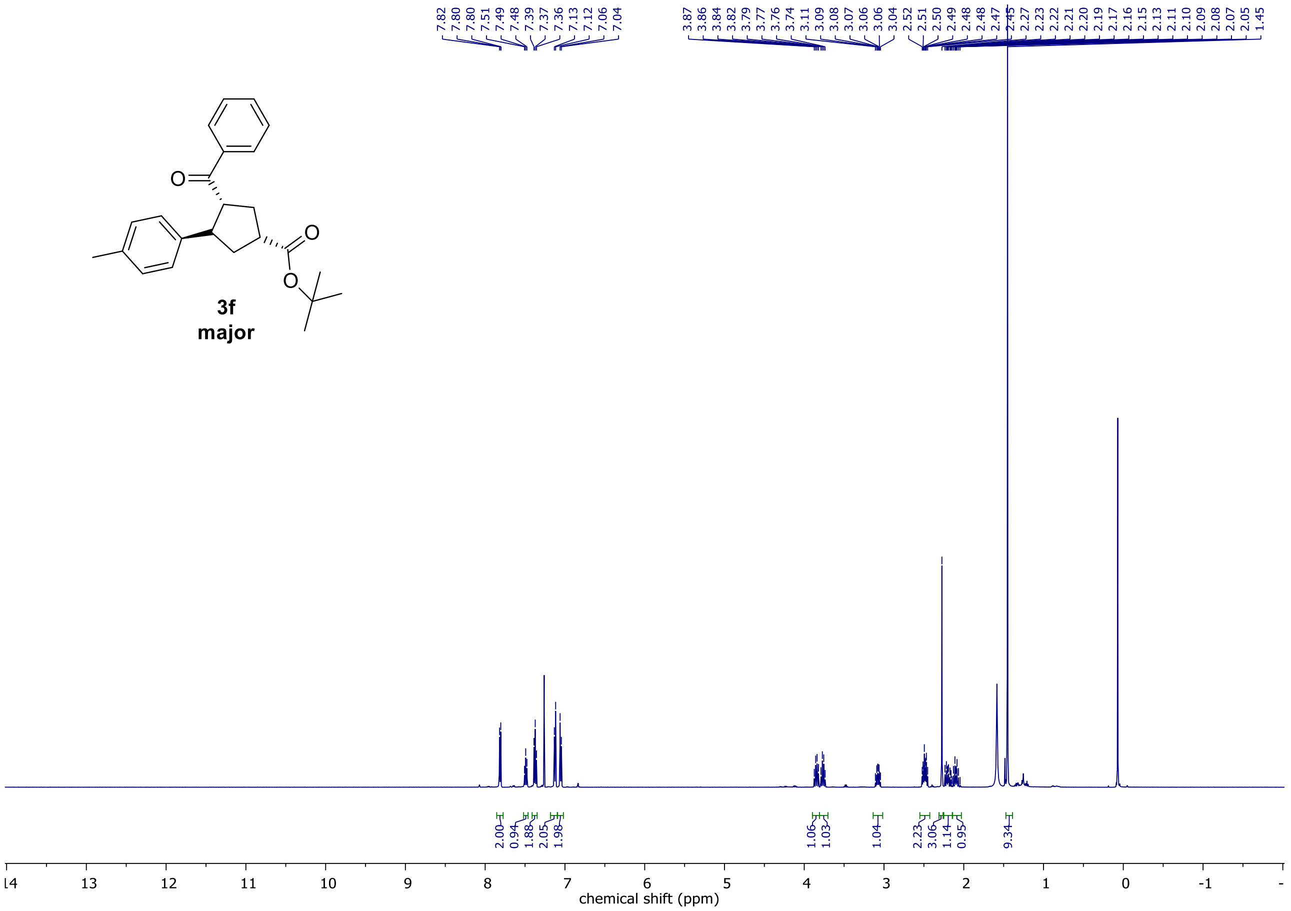

3f
major
7.82
7.80
7.80
7.51
7.49
7.48
7.39
7.37
7.36
7.13
7.12
7.06
7.04
3.87
3.86
3.84
3.82
3.79
3.77
3.76
3.74
3.11
3.09
3.08
3.07
3.06
3.06
3.04
2.52
2.51
2.50
2.49
2.48
2.48
2.47
2.45
2.27
2.23
2.22
2.21
2.20
2.19
2.17
2.16
2.15
2.13
2.11
2.10
2.09
2.08
2.07
2.05
1.45
2.00
0.94
1.88
2.05
1.98
1.06
1.03
1.04
2.23
3.06
1.14
0.95
9.34
14 13 12 11 10 9 8 7 6 5 4 3 2 1 0 -1
chemical shift (ppm)

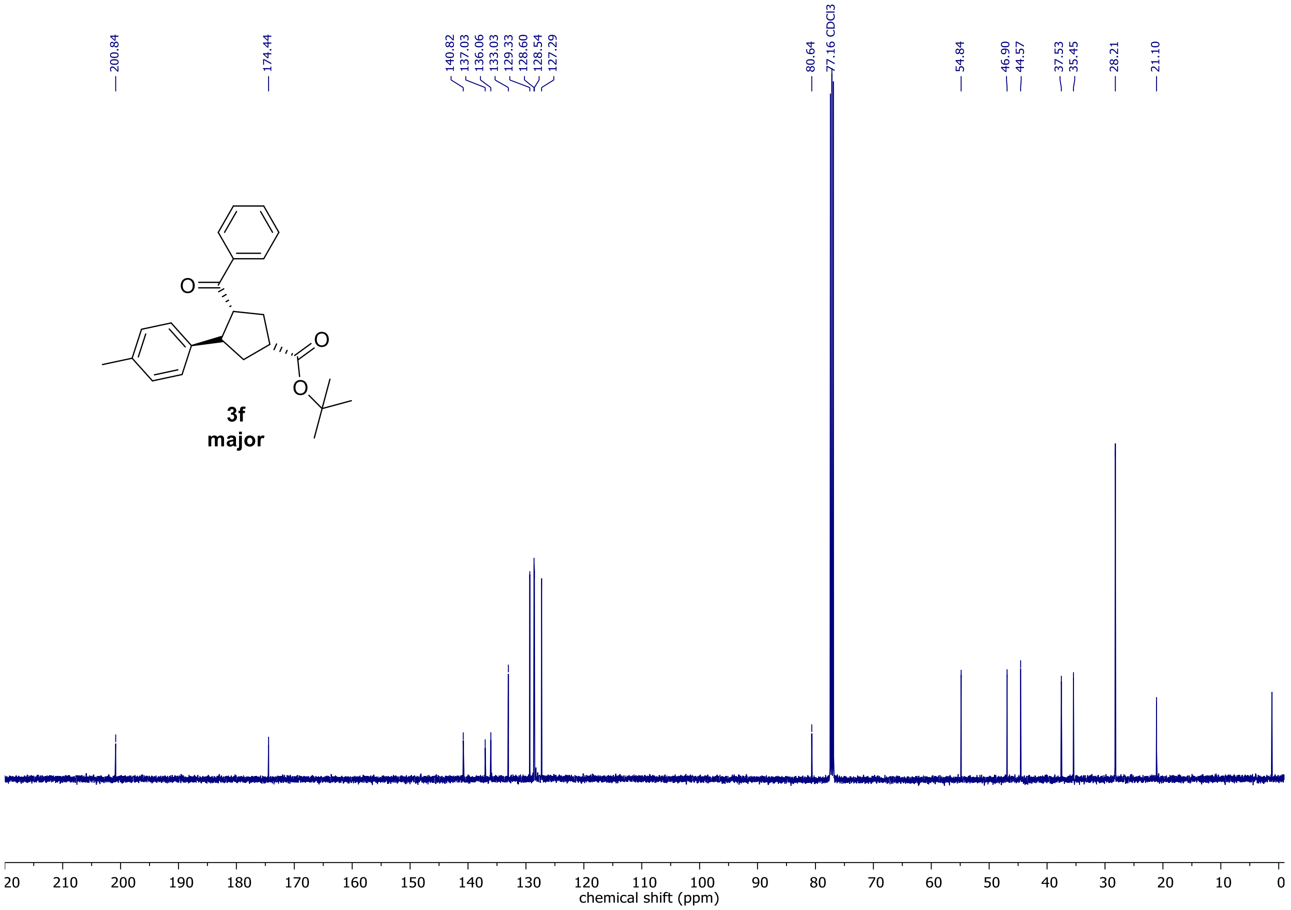

200.84
174.44
140.82
137.03
136.06
133.03
129.33
128.60
128.54
127.29
80.64
77.16 CDCl3
54.84
46.90
44.57
37.53
35.45
28.21
21.10
3f
major
220 210 200 190 180 170 160 150 140 130 120 110 100 90 80 70 60 50 40 30 20 10 0
chemical shift (ppm)

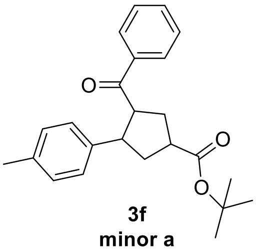

3f
minor a

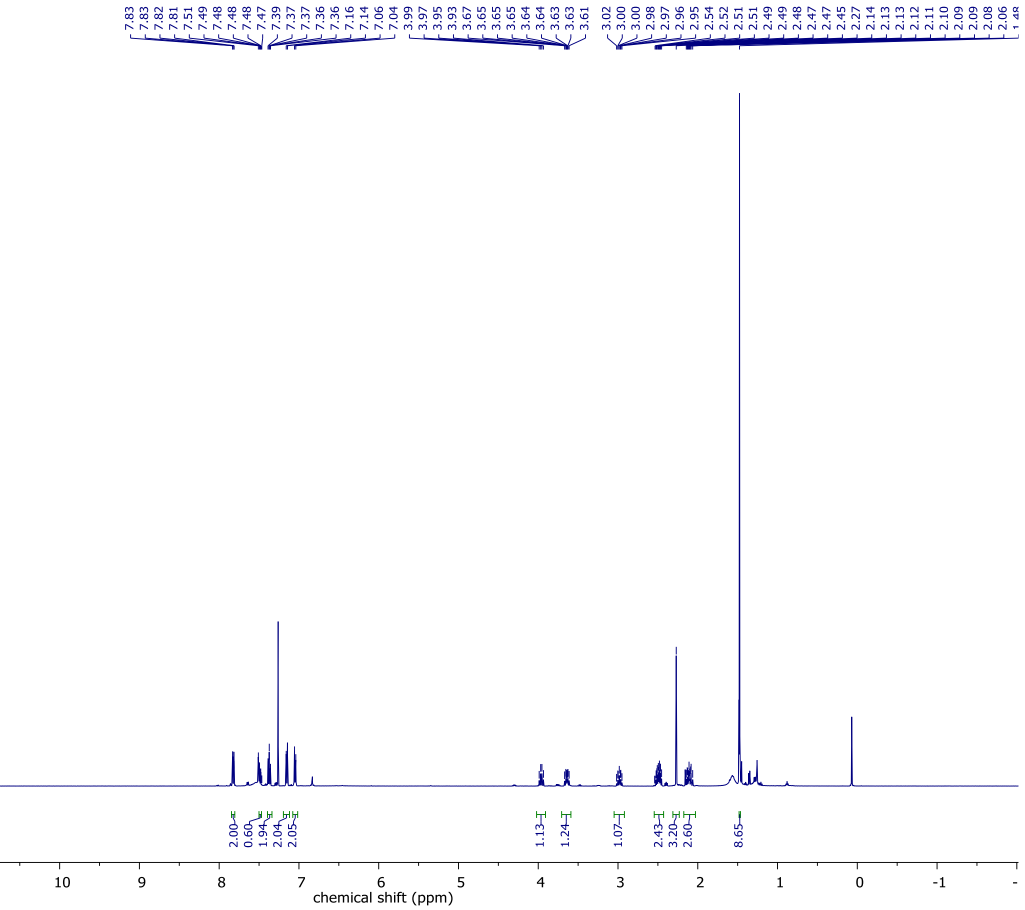

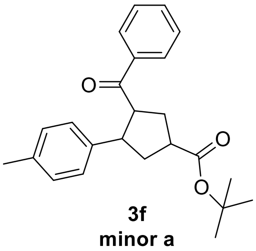

3f
minor a

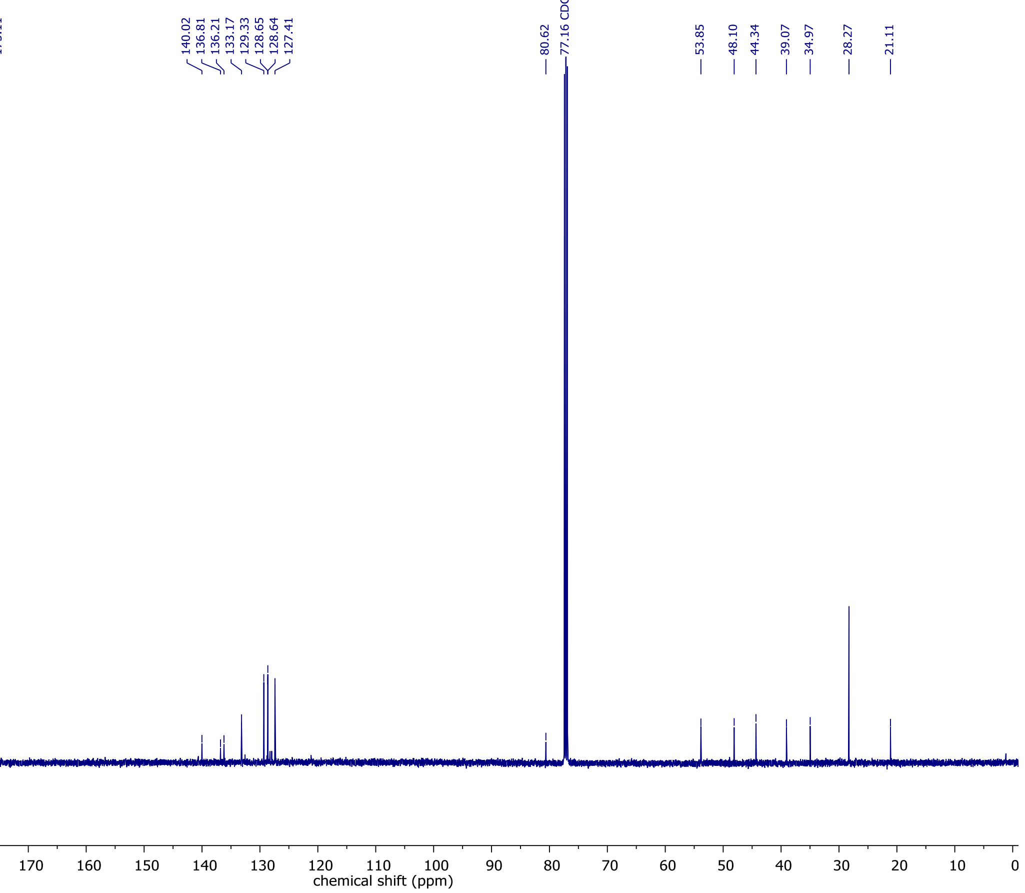

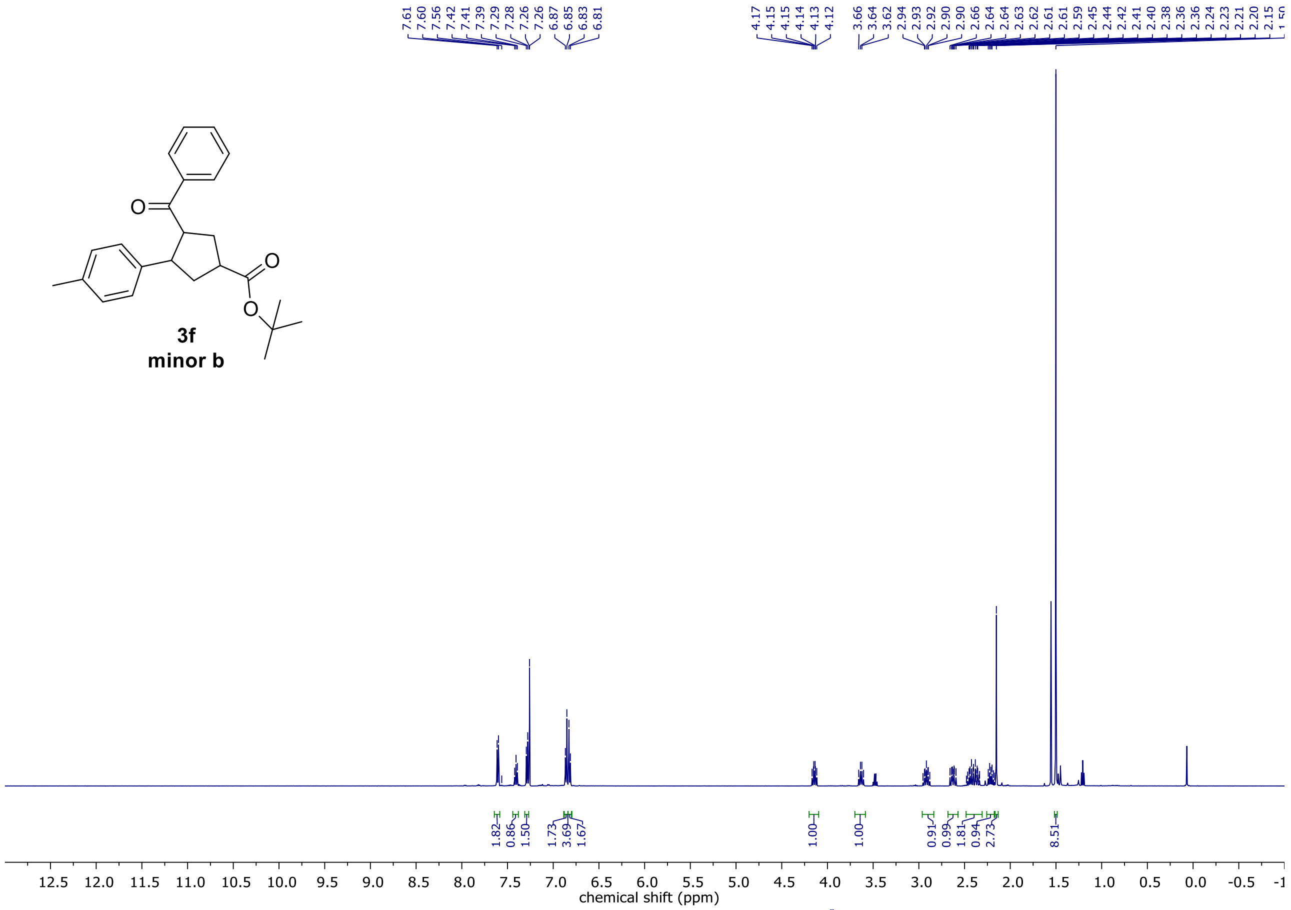

7.61 7.60 7.56 7.42 7.41 7.39 7.29 7.28 7.26 7.26 6.87 6.85 6.83 6.81
4.17 4.15 4.15 4.14 4.13 4.12 3.66 3.64 3.62 2.94 2.93 2.92 2.90 2.66 2.64 2.64 2.63 2.62 2.61 2.61 2.59 2.45 2.44 2.42 2.41 2.40 2.38 2.36 2.36 2.24 2.23 2.21 2.20 2.15
O
O
O
3f
minor b
1.82 0.86 1.50 1.73 3.69 1.67 1.00 1.00 0.91 0.99 1.81 0.94 2.73 8.51
12.5 12.0 11.5 11.0 10.5 10.0 9.5 9.0 8.5 8.0 7.5 7.0 6.5 6.0 5.5 5.0 4.5 4.0 3.5 3.0 2.5 2.0 1.5 1.0 0.5 0.0 -0.5
chemical shift (ppm)

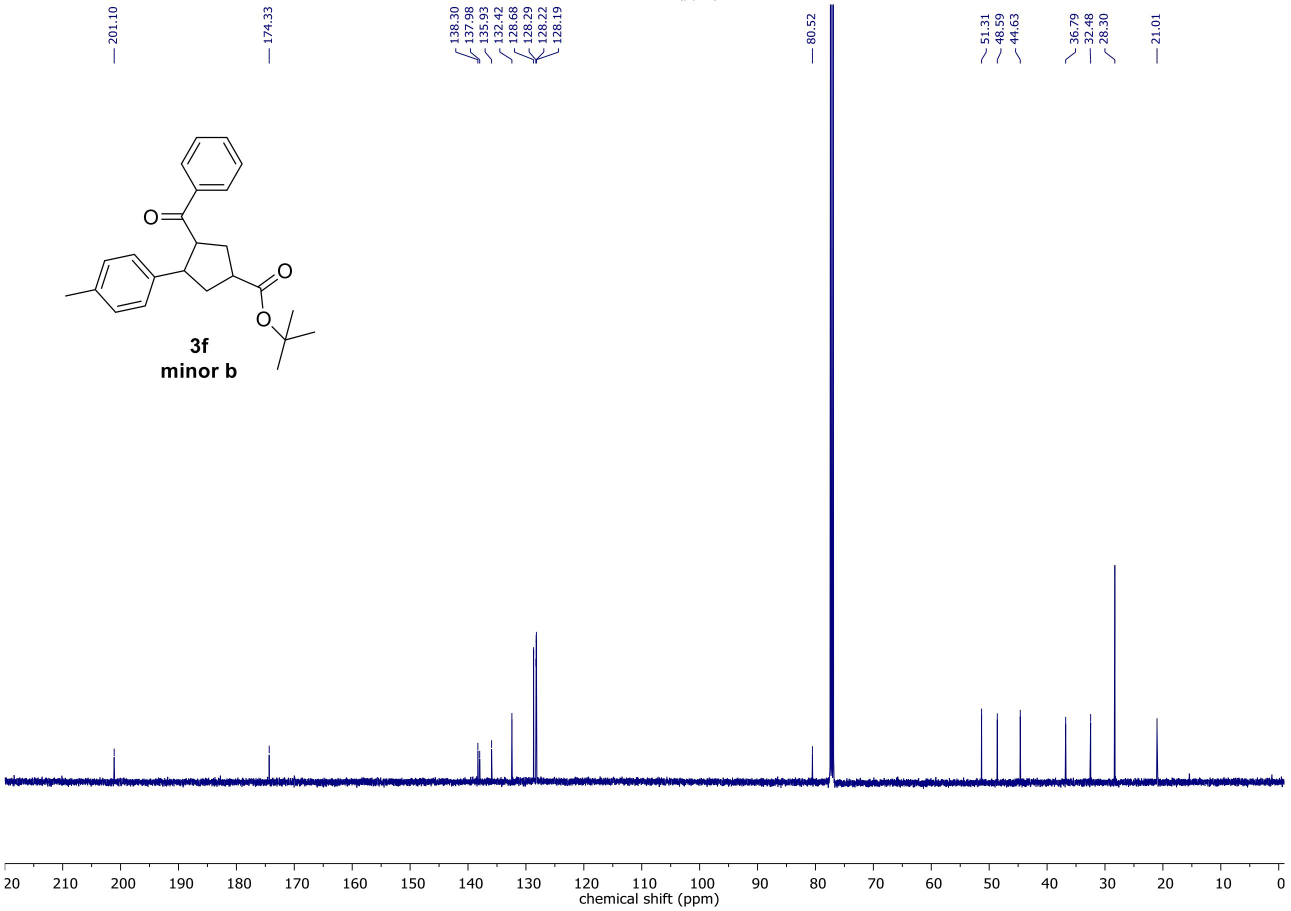

201.10
174.33
138.30 137.98 135.93 132.42 128.68 128.29 128.22 128.19
80.52
51.31 48.59 44.63
36.79 32.48 28.30
21.01
O
O
O
3f
minor b
210 200 190 180 170 160 150 140 130 120 110 100 90 80 70 60 50 40 30 20 10 0
chemical shift (ppm)

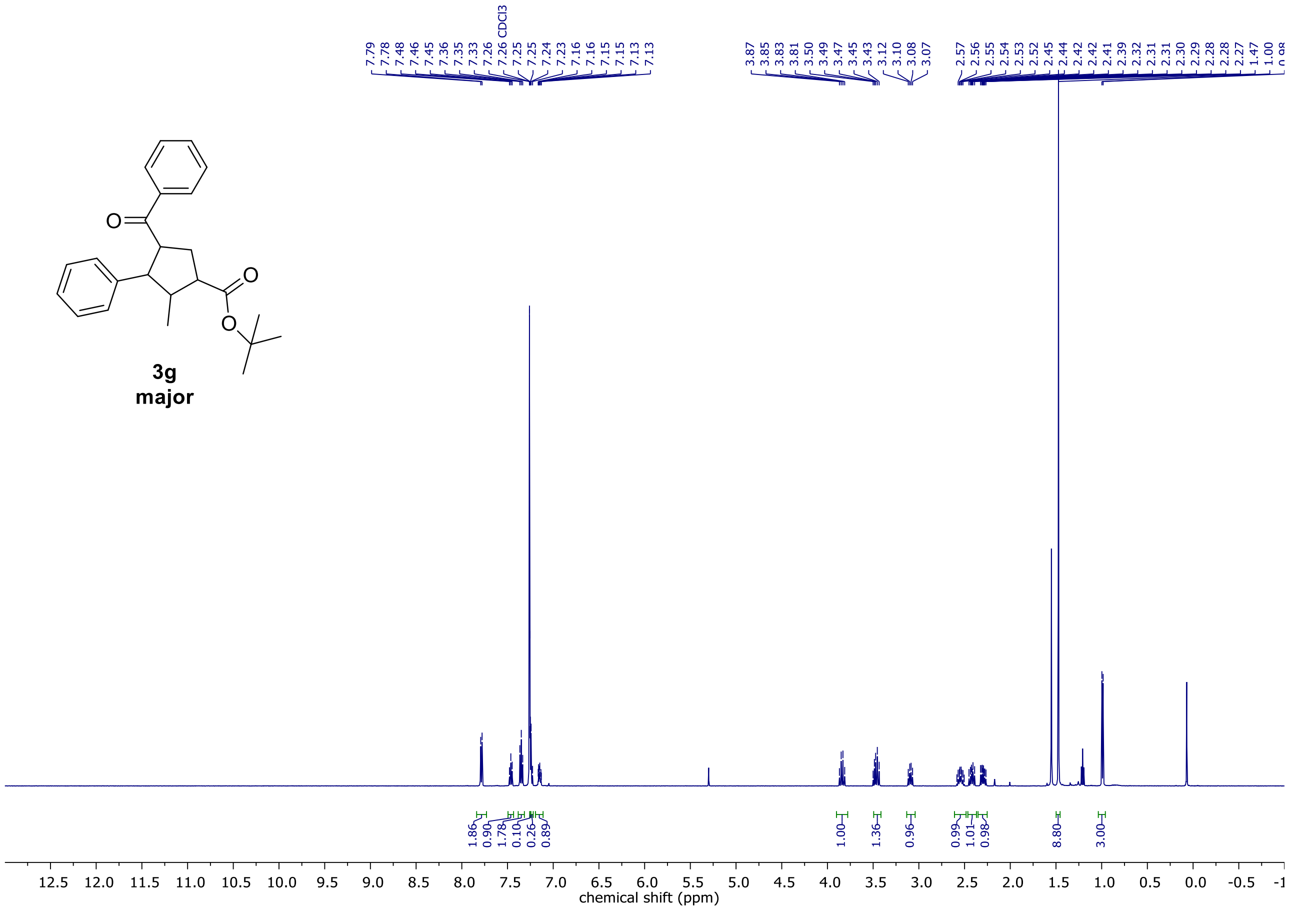
7.79 7.78 7.48 7.46 7.45 7.36 7.35 7.33 7.26 CDCl3 7.25 7.25 7.24 7.23 7.16 7.16 7.15 7.13 7.13
3.87 3.85 3.83 3.81 3.50 3.49 3.47 3.45 3.43 3.12 3.10 3.08 3.07
2.57 2.56 2.55 2.54 2.53 2.52 2.45 2.44 2.42 2.42 2.41 2.39 2.32 2.31 2.30 2.29 2.28 2.28 2.27 1.47 1.00
1.86 0.90 1.78 0.10 0.26 0.89 1.00 1.36 0.96 0.99 1.01 0.98 8.80 3.00
3g
major
chemical shift (ppm)

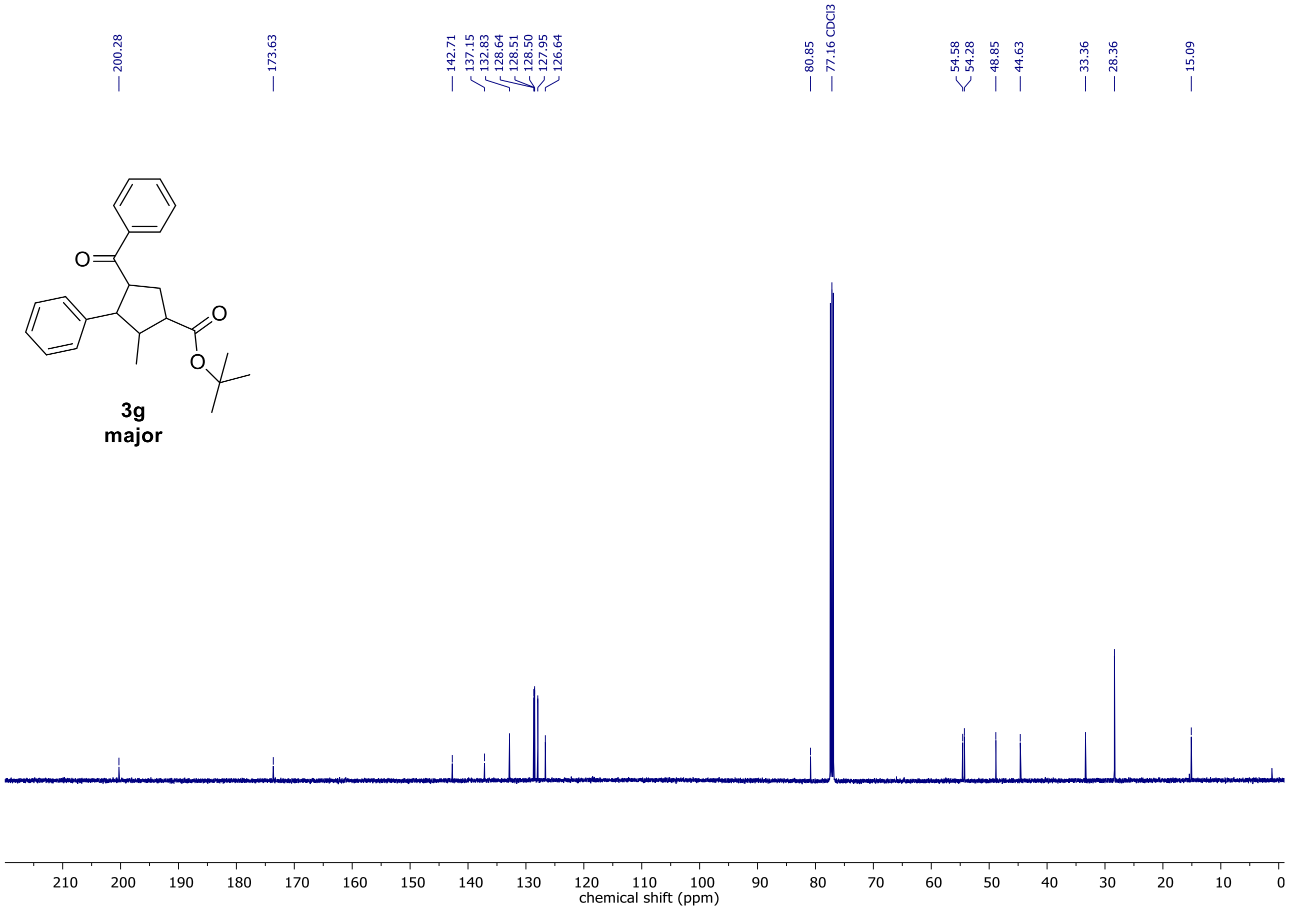
200.28 173.63 142.71 137.15 132.83 128.64 128.51 128.50 127.95 126.64 80.85 77.16 CDCl3 54.58 54.28 48.85 44.63 33.36 28.36 15.09
3g
major
chemical shift (ppm)

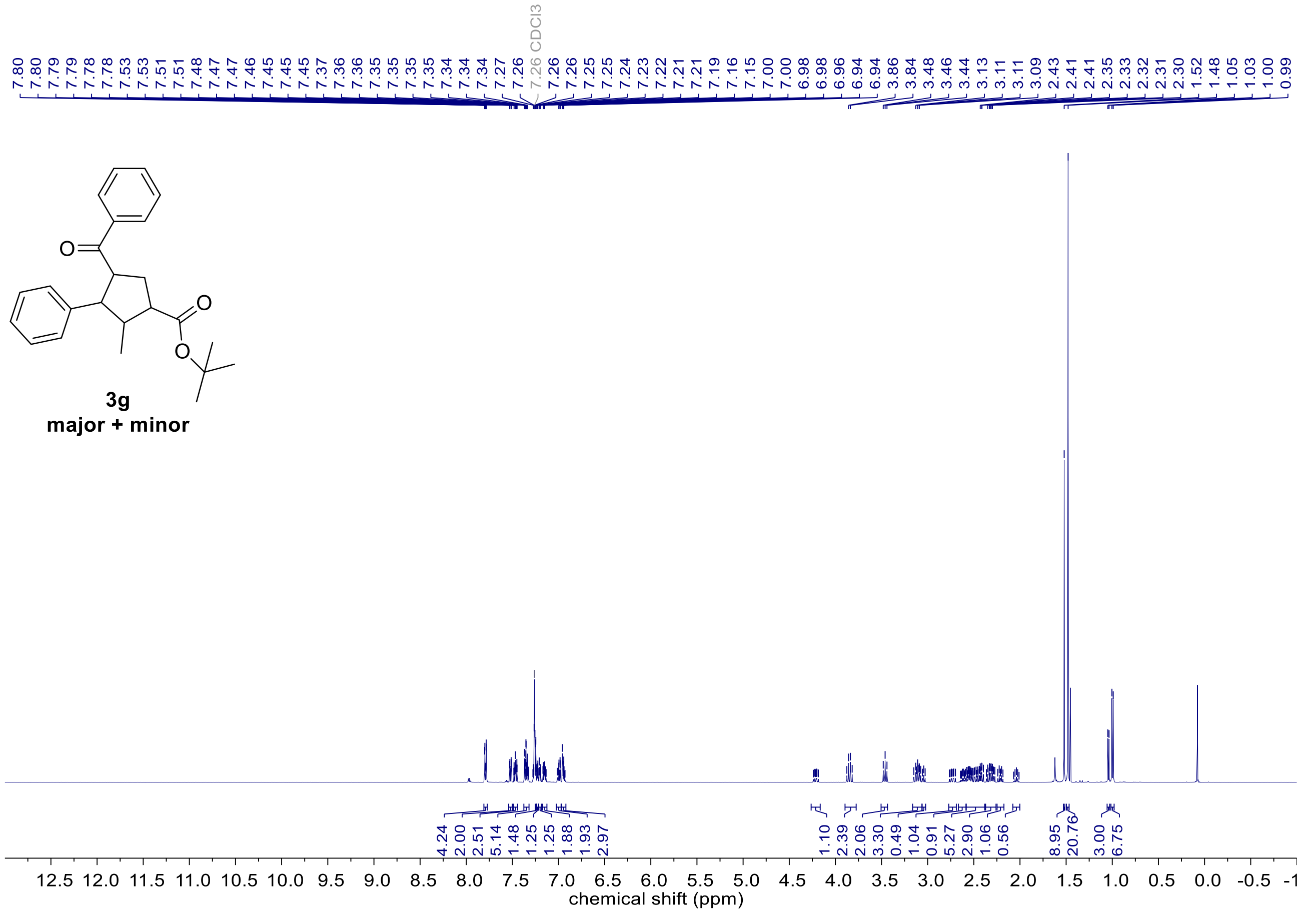

3g
major + minor

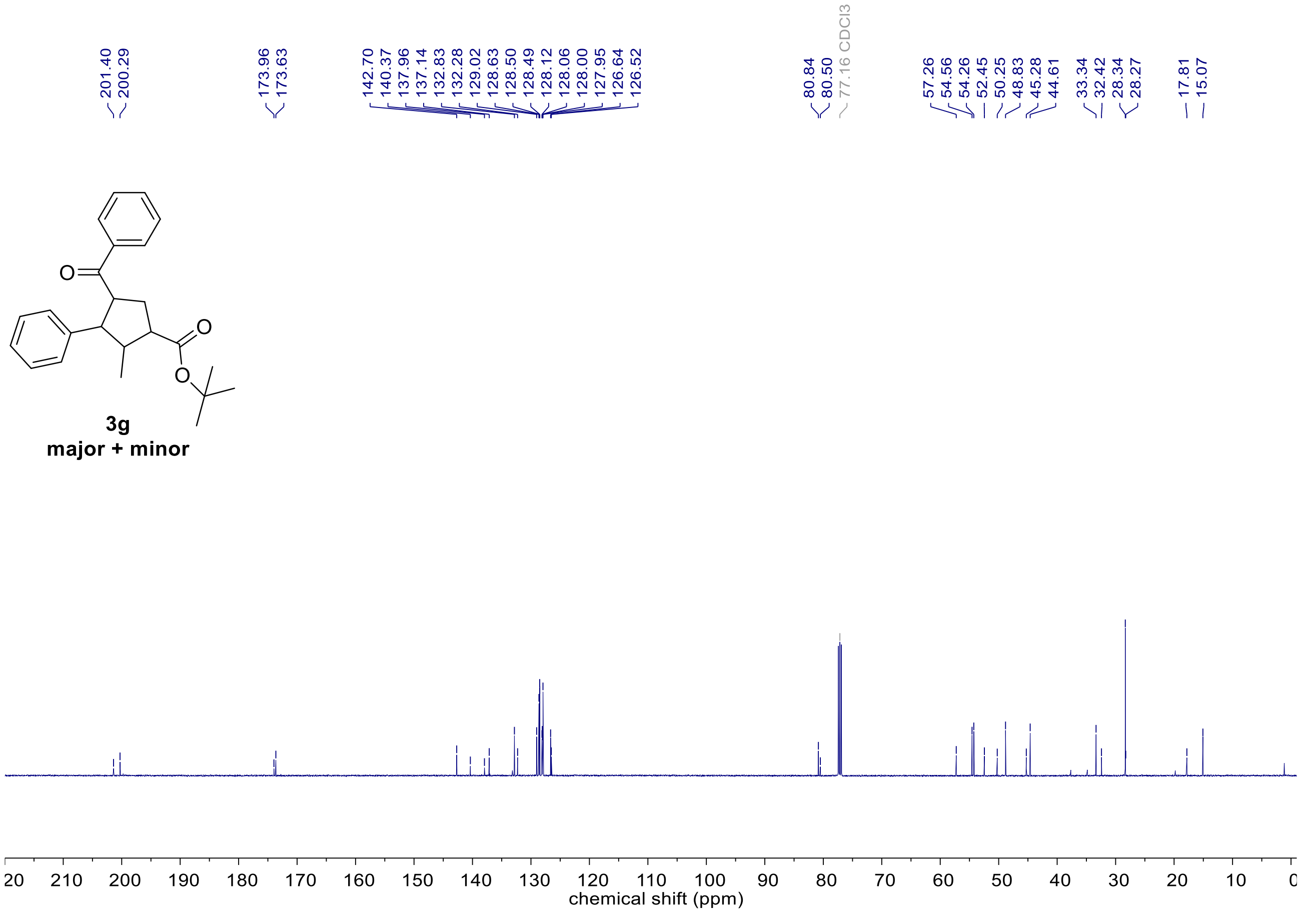

3g
major + minor

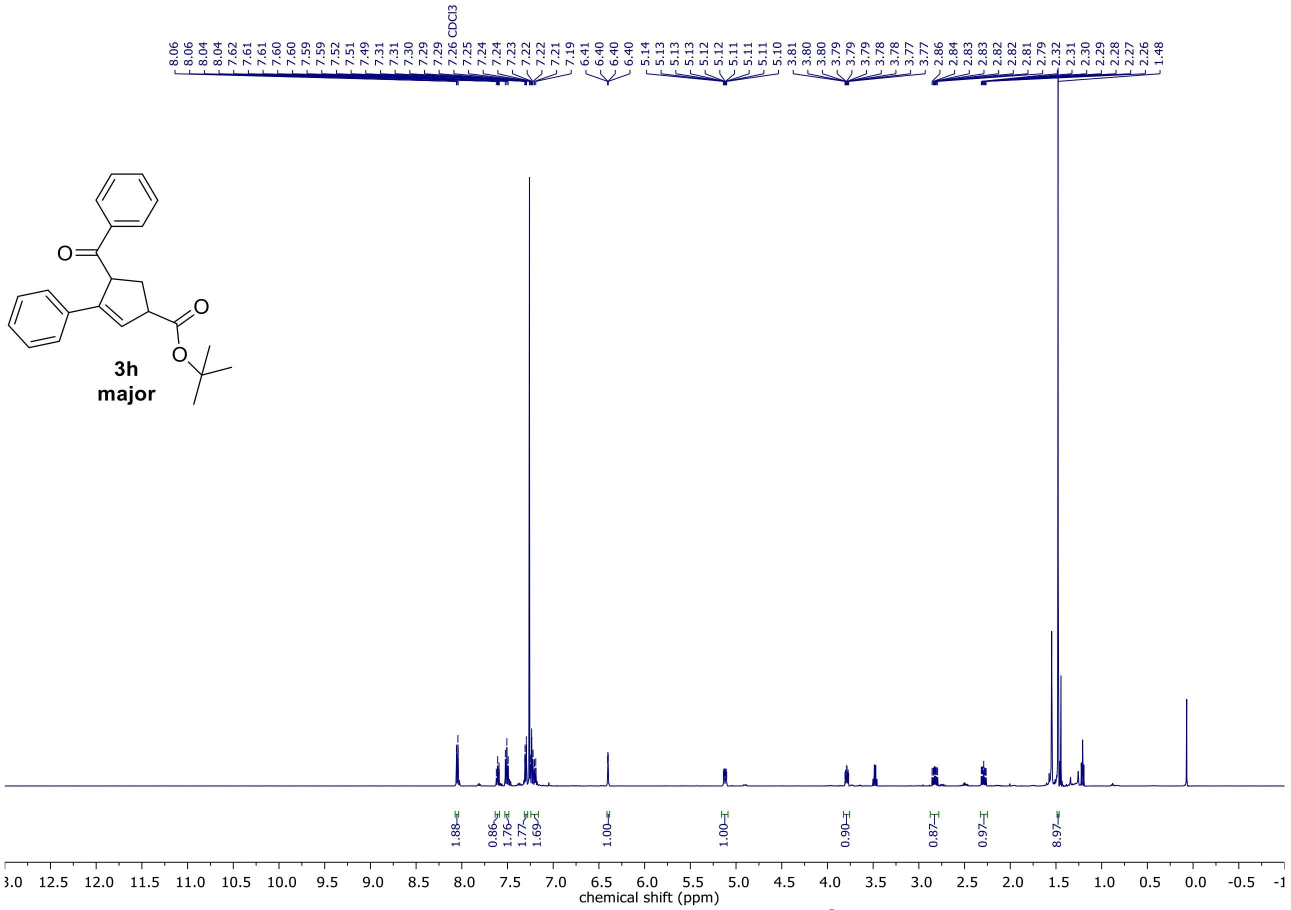
8.06 8.06 8.04 8.04 7.62 7.61 7.61 7.60 7.60 7.59 7.59 7.52 7.51 7.49 7.31 7.31 7.30 7.29 7.29 7.26 CDCl3 7.25 7.24 7.24 7.23 7.22 7.22 7.22 7.21 7.19 6.41 6.40 6.40 6.40 5.14 5.13 5.13 5.13 5.12 5.12 5.11 5.11 5.11 5.10 3.81 3.80 3.80 3.79 3.79 3.78 3.78 3.77 3.77 2.86 2.84 2.83 2.83 2.82 2.82 2.81 2.79 2.32 2.31 2.30 2.29 2.28 2.27 2.26 1.48
3h
major
1.88 0.86 1.76 1.77 1.69 1.00 1.00 0.90 0.87 0.97 8.97
chemical shift (ppm)

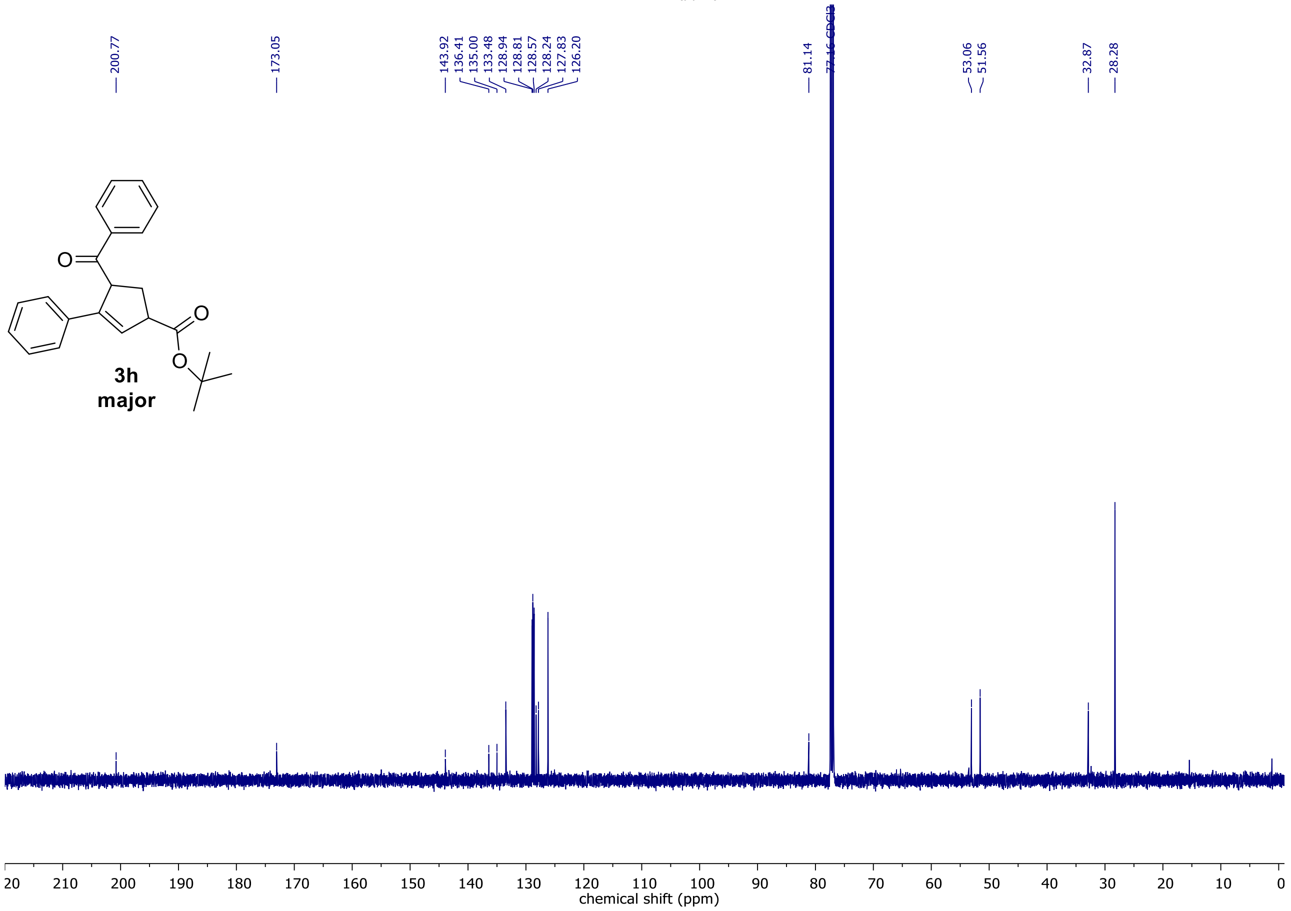
200.77 173.05 143.92 136.41 135.00 133.48 128.94 128.81 128.57 128.24 127.83 126.20 81.14 77.16 CDCl3 53.06 51.56 32.87 28.28
3h
major
chemical shift (ppm)

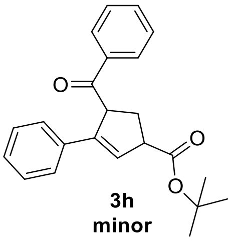

3h
minor

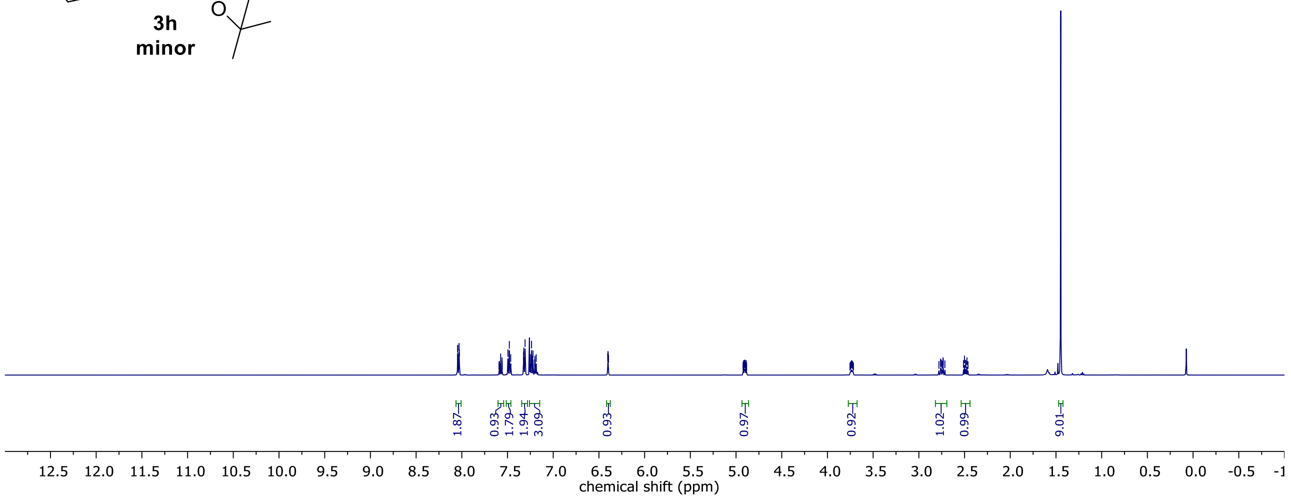

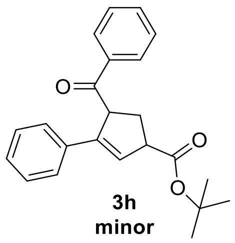

3h
minor

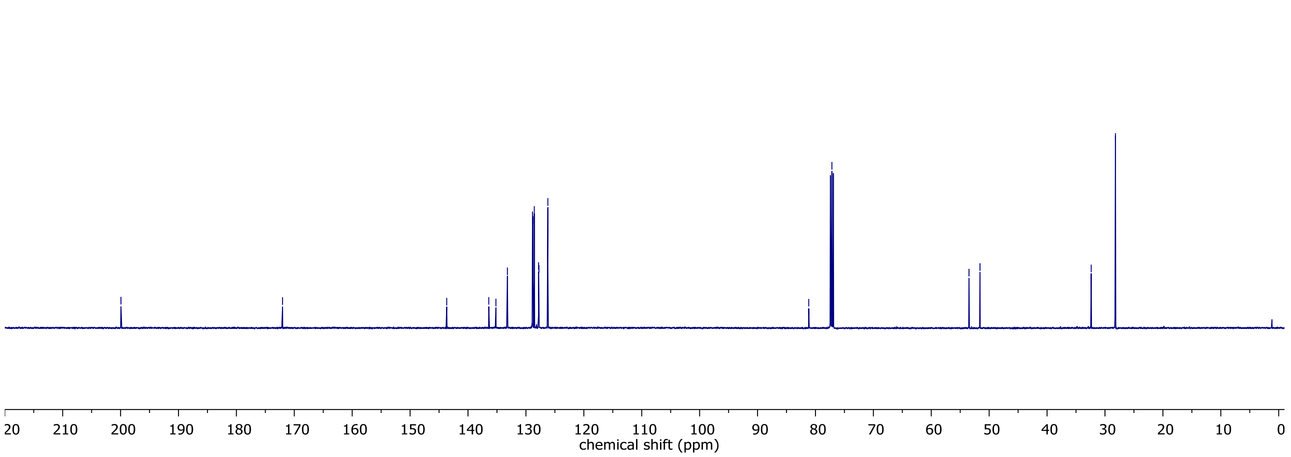

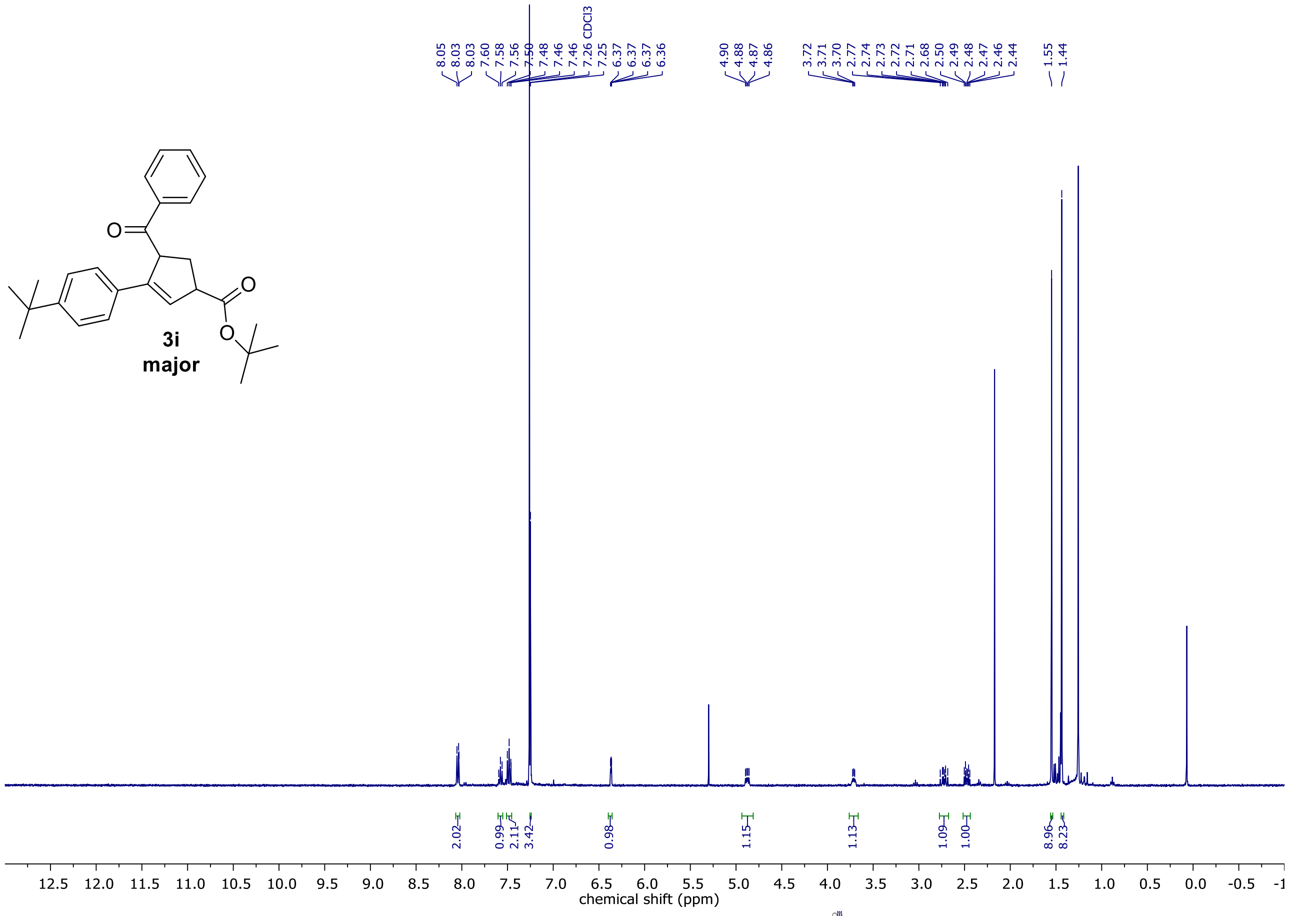
8.05
8.03
8.03
7.60
7.58
7.56
7.50
7.48
7.46
7.46
7.26 CDCl3
7.25
6.37
6.37
6.37
6.36
4.90
4.88
4.87
4.86
3.72
3.71
3.70
2.77
2.74
2.73
2.72
2.71
2.68
2.50
2.49
2.48
2.47
2.46
2.44
1.55
1.44
O
O
O
3i
major
2.02
0.99
2.11
3.42
0.98
1.15
1.13
1.09
1.00
8.96
8.23
chemical shift (ppm)

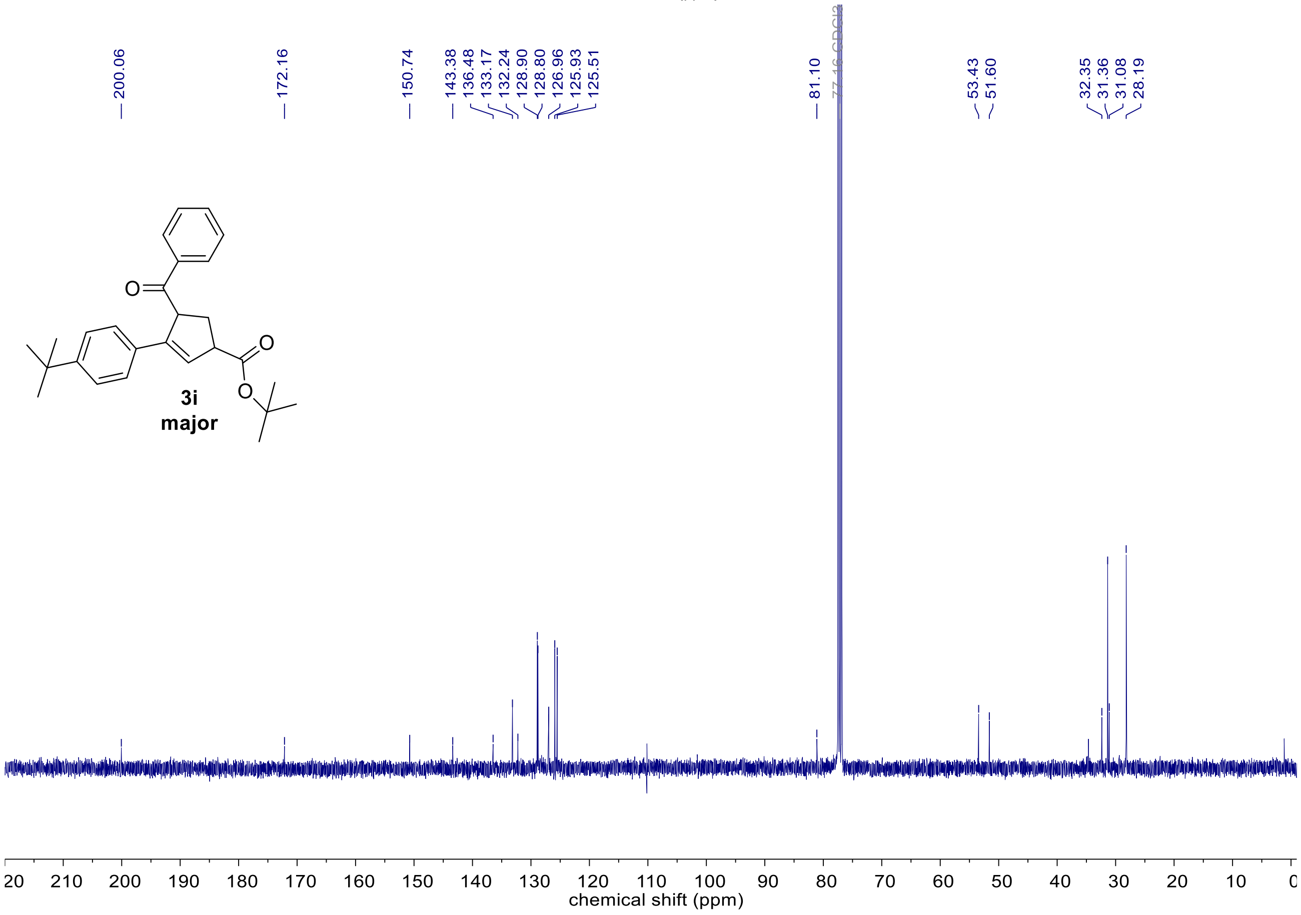
200.06
172.16
150.74
143.38
136.48
133.17
132.24
128.90
128.80
126.96
125.93
125.51
81.10
77.16 CDCl3
53.43
51.60
32.35
31.36
31.08
28.19
O
O
O
3i
major
chemical shift (ppm)

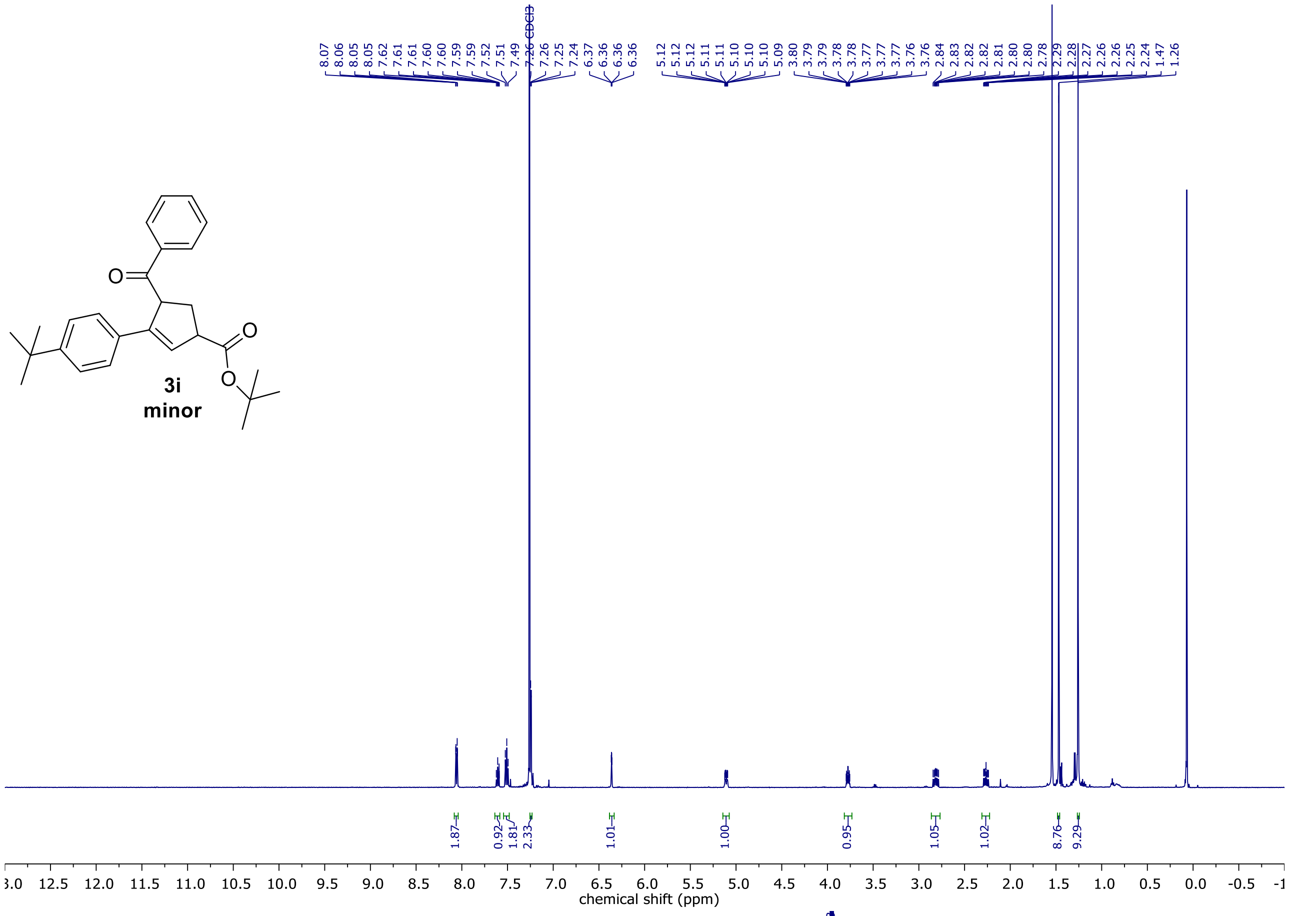
3i
minor
chemical shift (ppm)

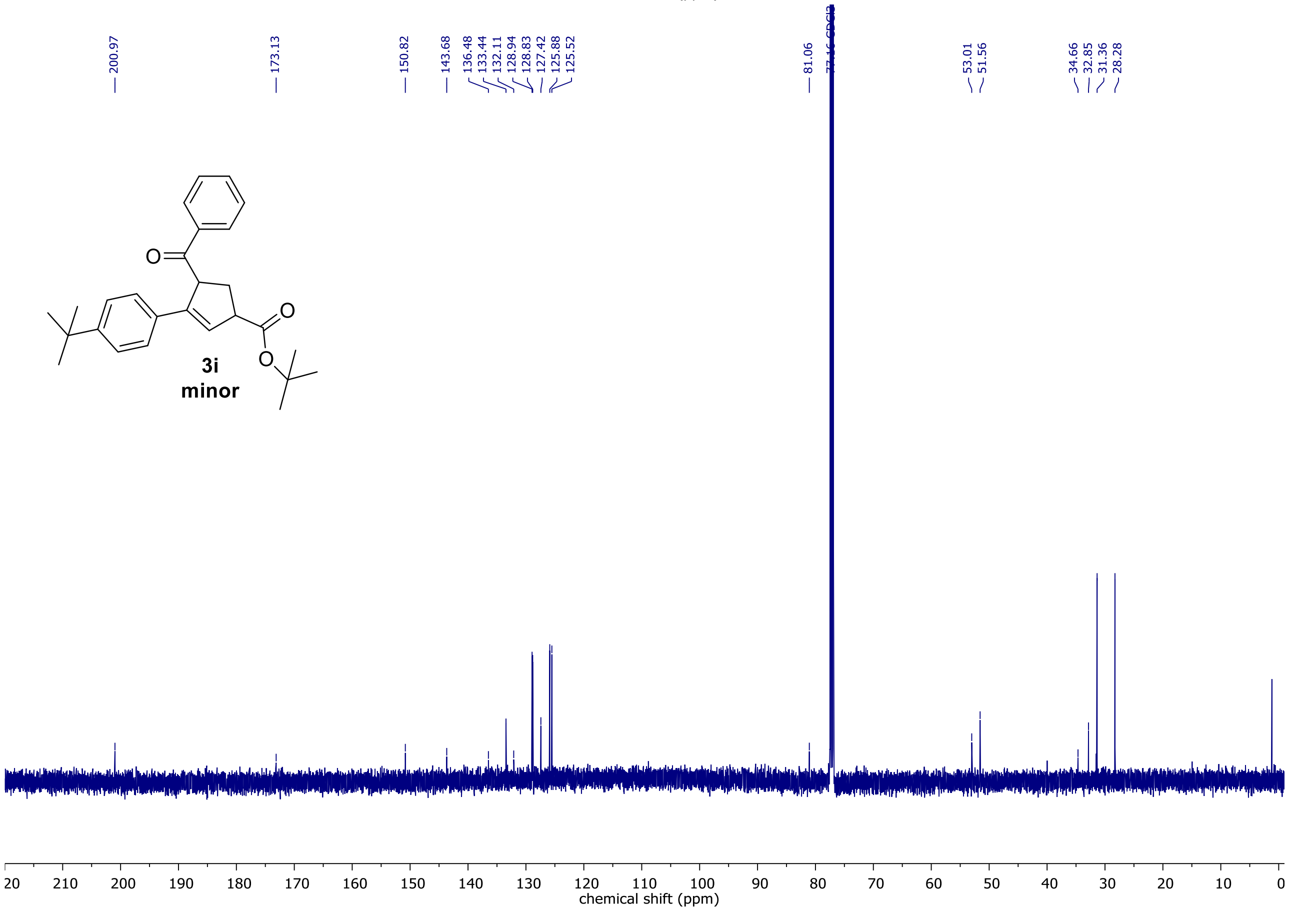
3i
minor
chemical shift (ppm)

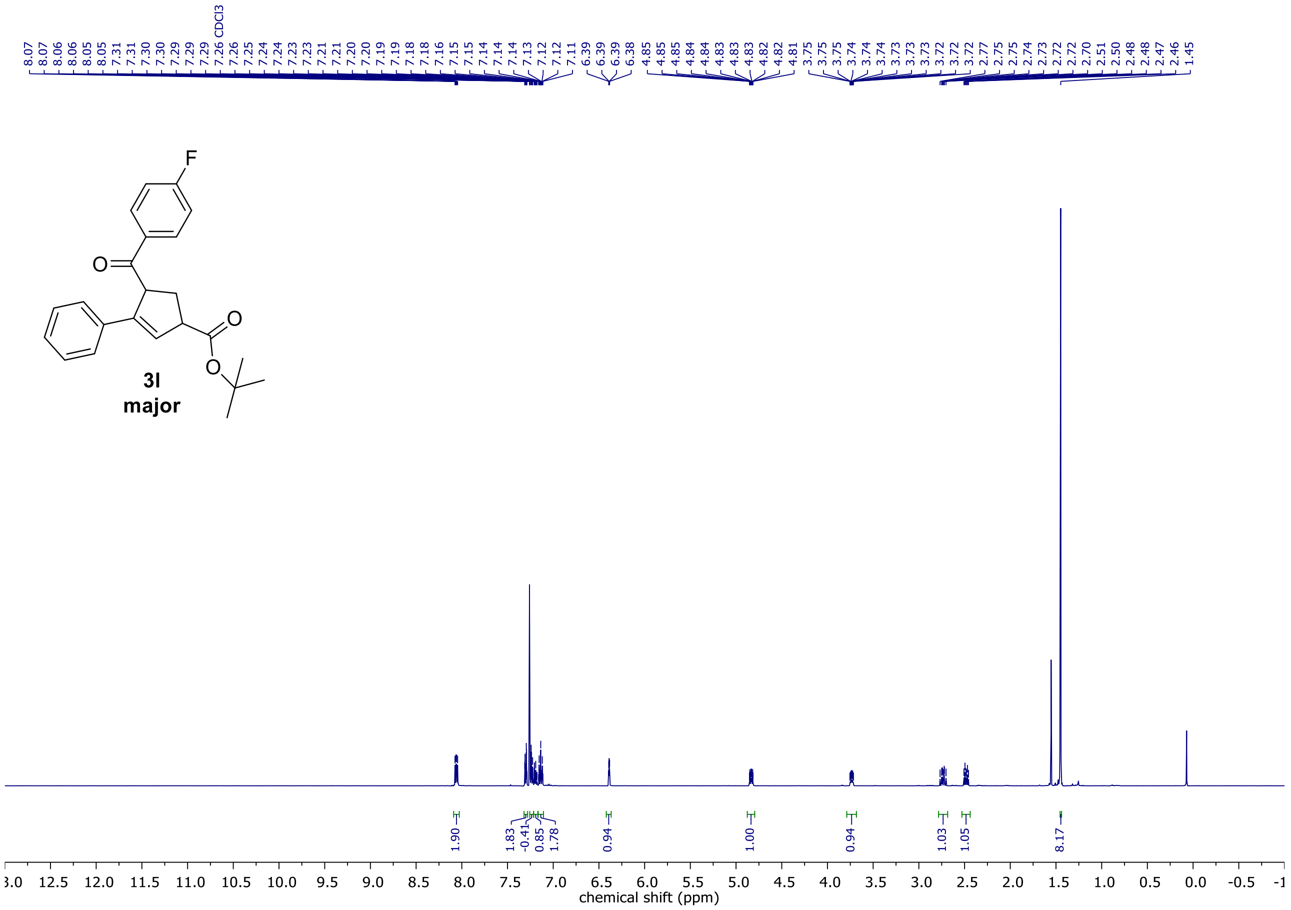
3l
major
chemical shift (ppm)

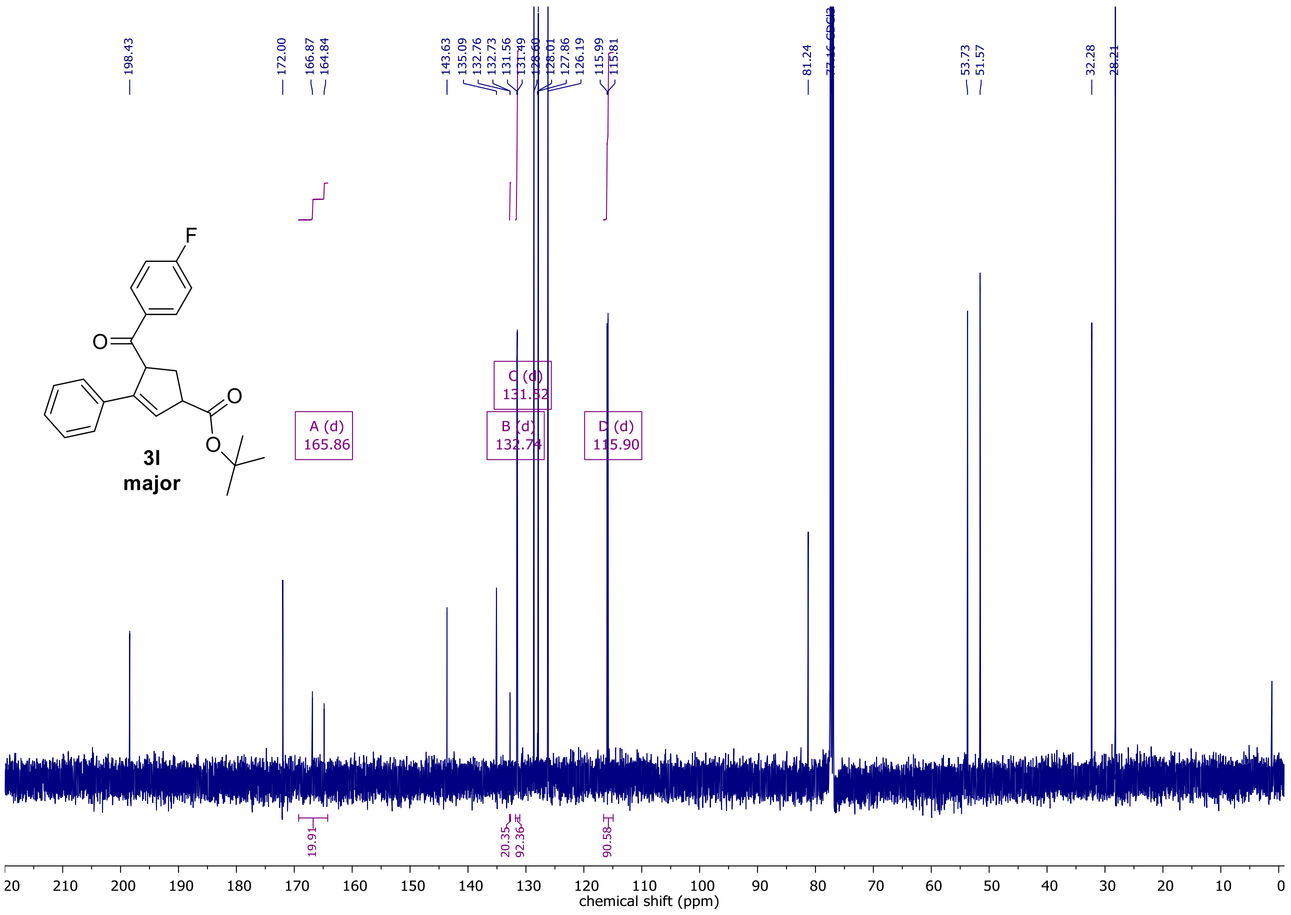
3l
major
A (d)
165.86
B (d)
132.74
C (d)
131.52
D (d)
115.90
chemical shift (ppm)

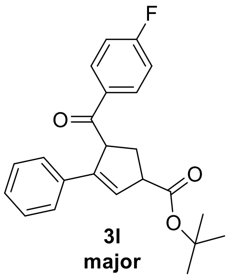

3l
major

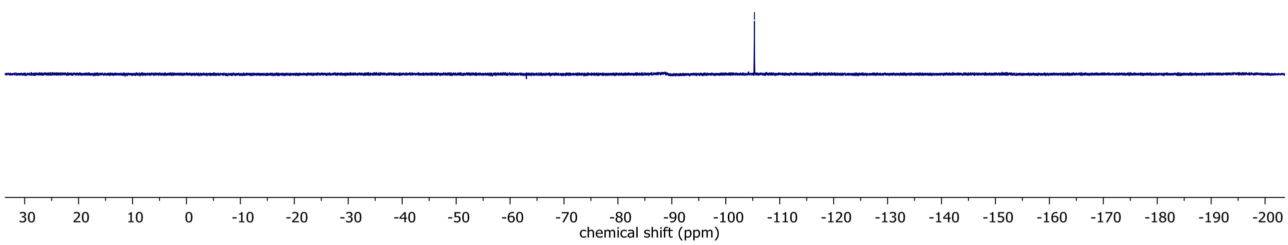
-105.31
30 20 10 0 -10 -20 -30 -40 -50 -60 -70 -80 -90 -100 -110 -120 -130 -140 -150 -160 -170 -180 -190 -200
chemical shift (ppm)

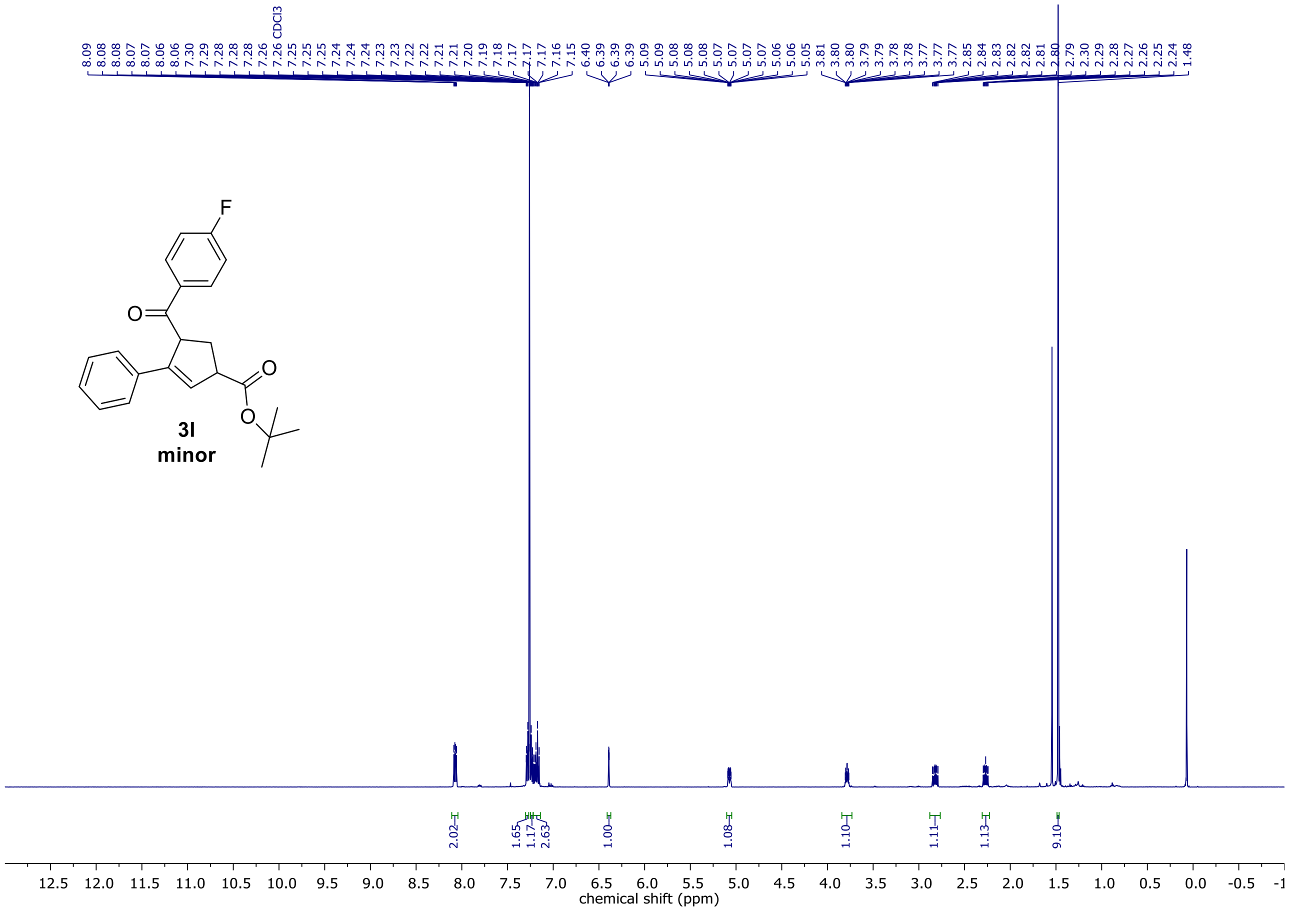

3l
minor
chemical shift (ppm)

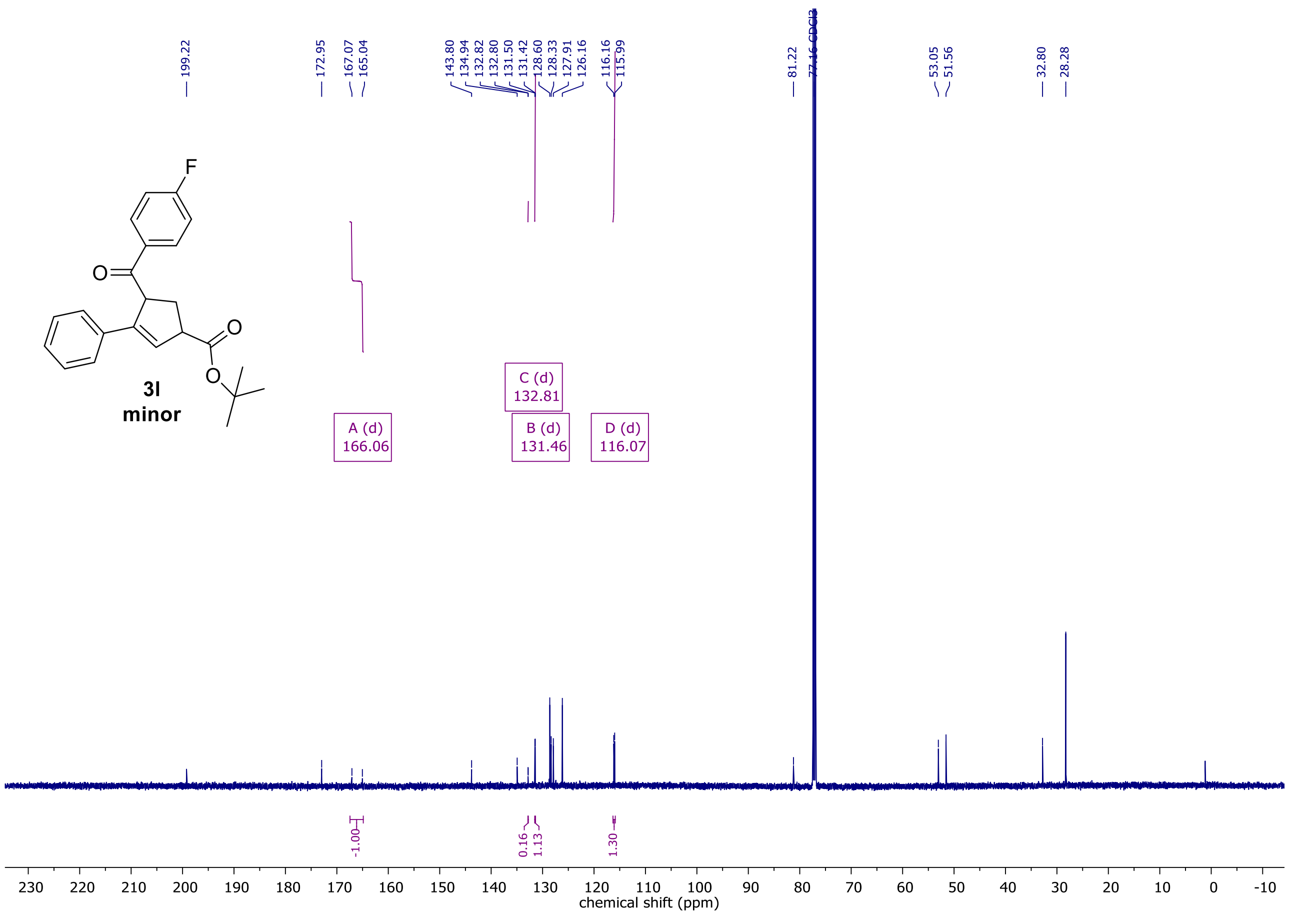
199.22
172.95
167.07
165.04
143.80
134.94
132.82
132.80
131.50
131.42
128.60
128.33
127.91
126.16
116.16
115.99
81.22
77.16 CDCl3
53.05
51.56
32.80
28.28
F
O
O
O
3l
minor
A (d)
166.06
C (d)
132.81
B (d)
131.46
D (d)
116.07
-1.00
0.16
1.13
1.30
230 220 210 200 190 180 170 160 150 140 130 120 110 100 90 80 70 60 50 40 30 20 10 0 -10
chemical shift (ppm)

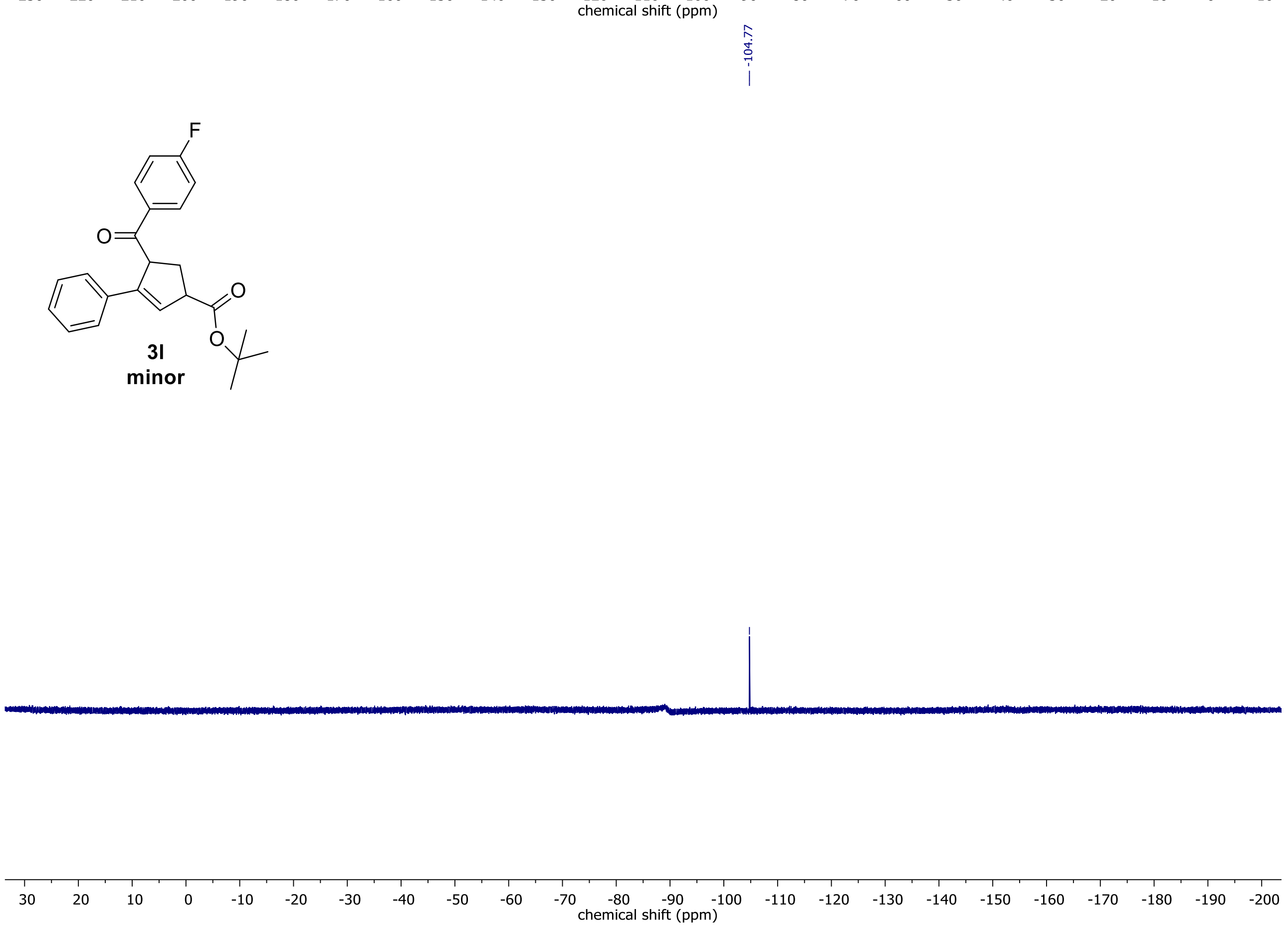
-104.77
F
O
O
O
3l
minor
30 20 10 0 -10 -20 -30 -40 -50 -60 -70 -80 -90 -100 -110 -120 -130 -140 -150 -160 -170 -180 -190 -200
chemical shift (ppm)

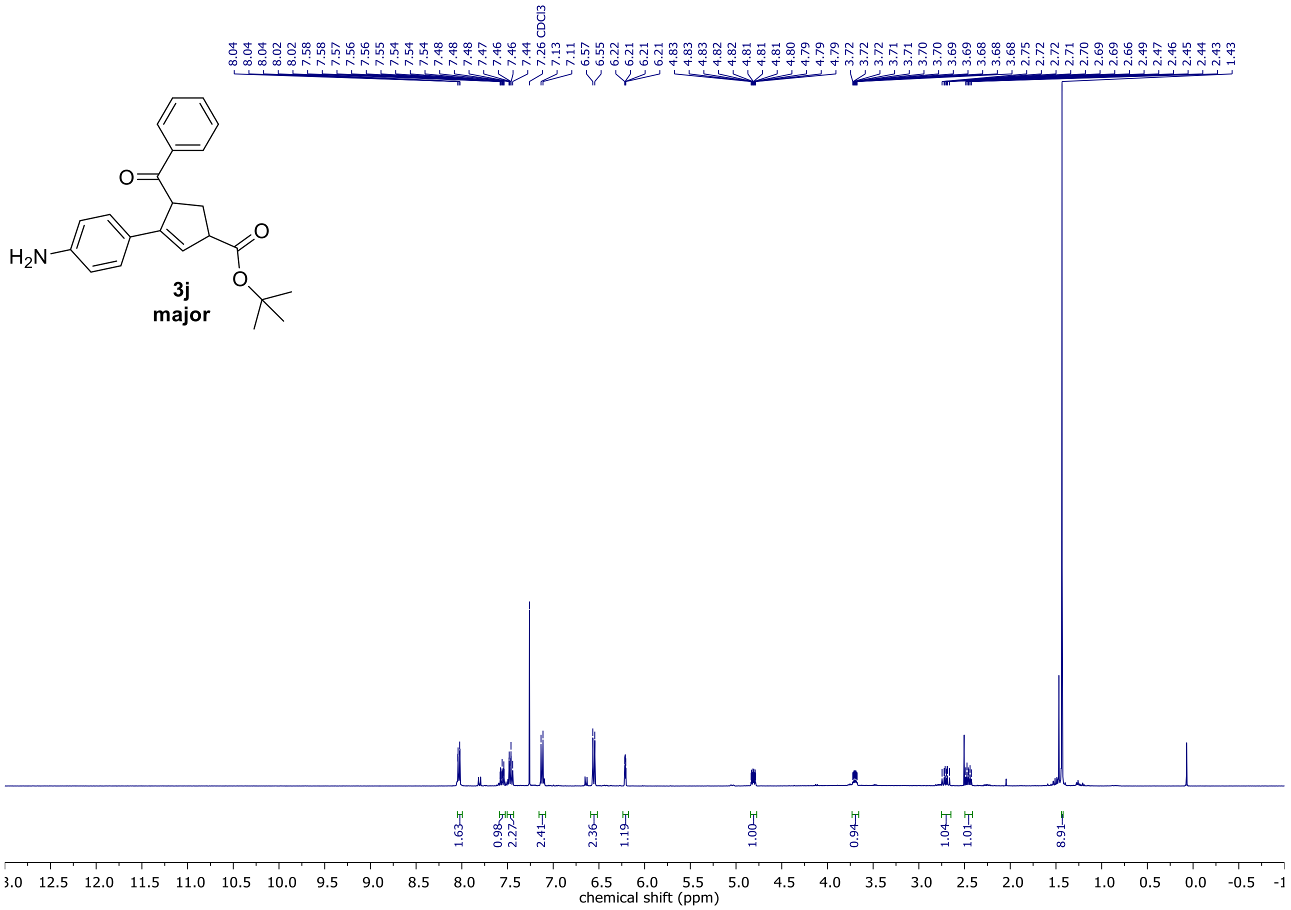
8.04 8.04 8.04 8.02 8.02 7.58 7.58 7.57 7.56 7.56 7.55 7.54 7.54 7.54 7.48 7.48 7.47 7.46 7.46 7.44 7.26 CDCl3 7.13 7.11 6.57 6.55 6.22 6.21 6.21 6.21 4.83 4.83 4.83 4.82 4.82 4.81 4.81 4.81 4.80 4.79 4.79 3.72 3.72 3.72 3.71 3.71 3.70 3.70 3.69 3.69 3.68 3.68 3.68 2.75 2.72 2.72 2.71 2.70 2.69 2.69 2.66 2.49 2.47 2.46 2.45 2.44 2.43 1.43
H_2N
O
3j
major
1.63
0.98
2.27
2.41
2.36
1.19
1.00
0.94
1.04
1.01
8.91
chemical shift (ppm)

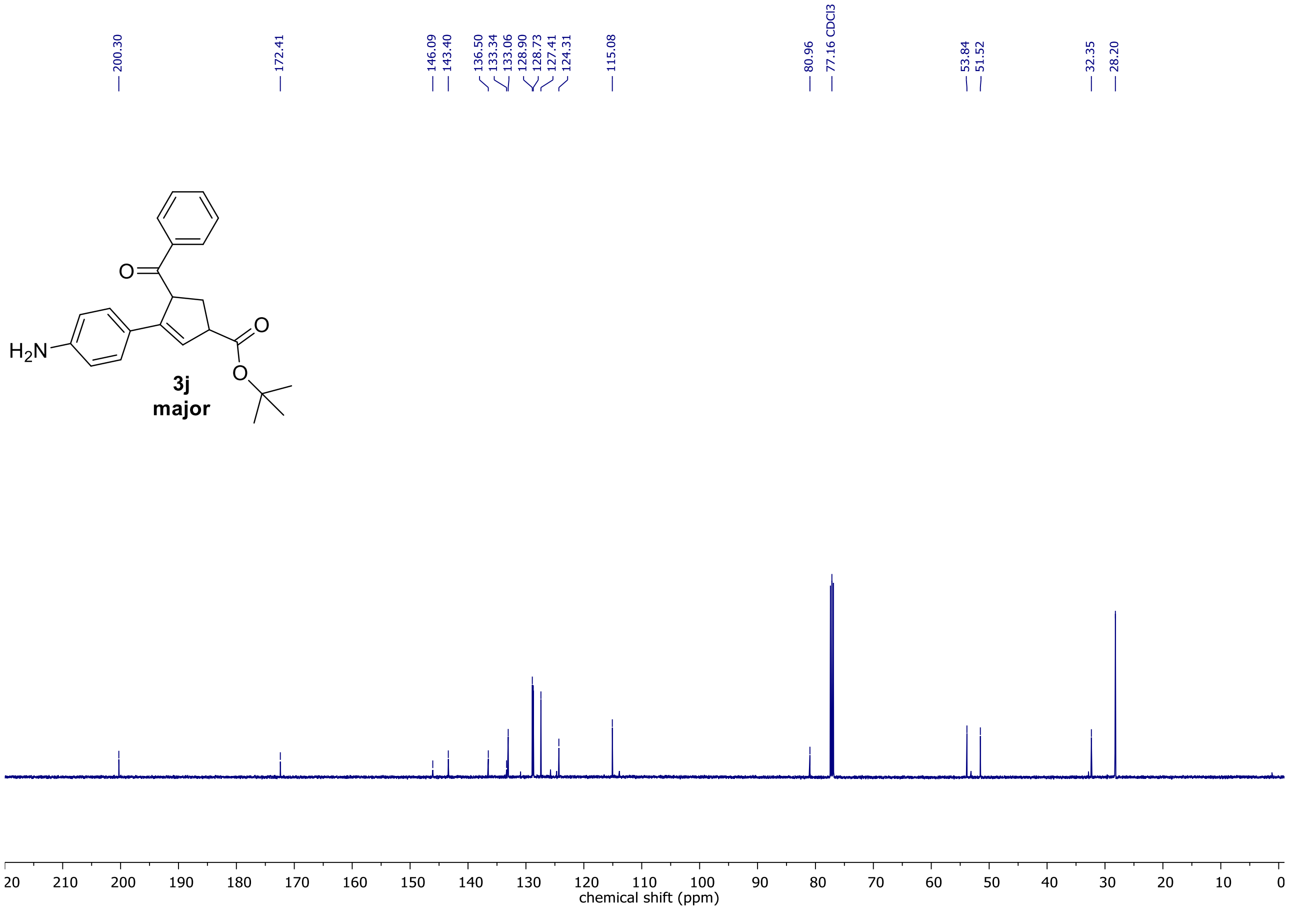
200.30
172.41
146.09
143.40
136.50
133.34
133.06
128.90
128.73
127.41
124.31
115.08
80.96
77.16 CDCl3
53.84
51.52
32.35
28.20
H_2N
O
3j
major
chemical shift (ppm)

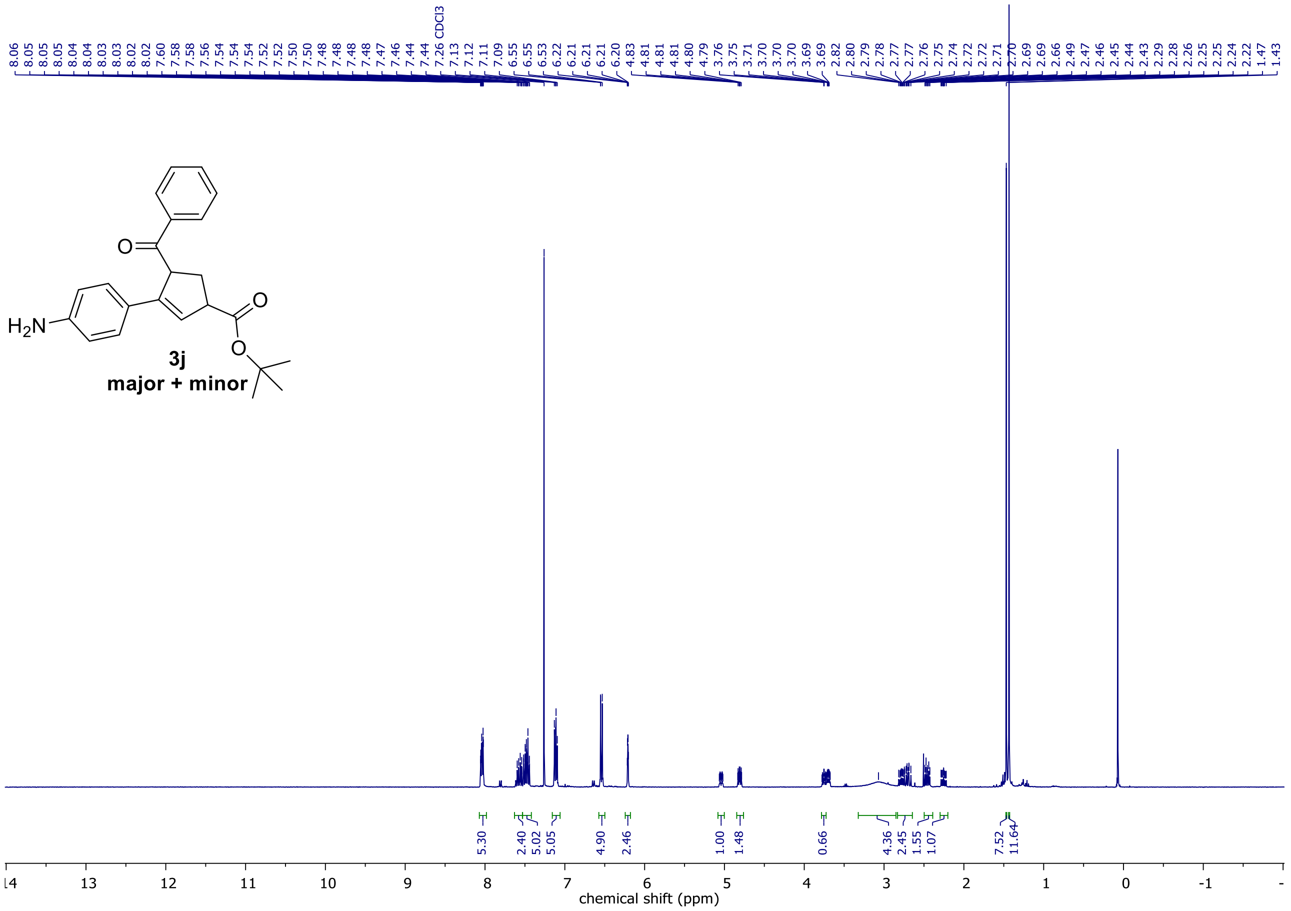

8.06 8.05 8.05 8.05 8.04 8.04 8.03 8.03 8.02 8.02 7.60 7.58 7.58 7.56 7.54 7.54 7.54 7.52 7.52 7.50 7.50 7.48 7.48 7.48 7.48 7.47 7.46 7.44 7.44 7.26 CDCl3 7.13 7.12 7.11 7.09 6.55 6.55 6.53 6.22 6.21 6.21 6.21 6.20 4.83 4.81 4.81 4.80 4.79 3.76 3.75 3.71 3.70 3.70 3.70 3.69 3.69 2.82 2.80 2.79 2.78 2.77 2.77 2.76 2.75 2.74 2.72 2.72 2.71 2.70 2.69 2.69 2.66 2.49 2.47 2.46 2.45 2.44 2.43 2.29 2.28 2.26 2.25 2.25 2.24 2.22 1.47 1.43
H2N
3j
major + minor
5.30 2.40 5.02 5.05 4.90 2.46 1.00 1.48 0.66 4.36 2.45 1.55 1.07 7.52 11.64
chemical shift (ppm)

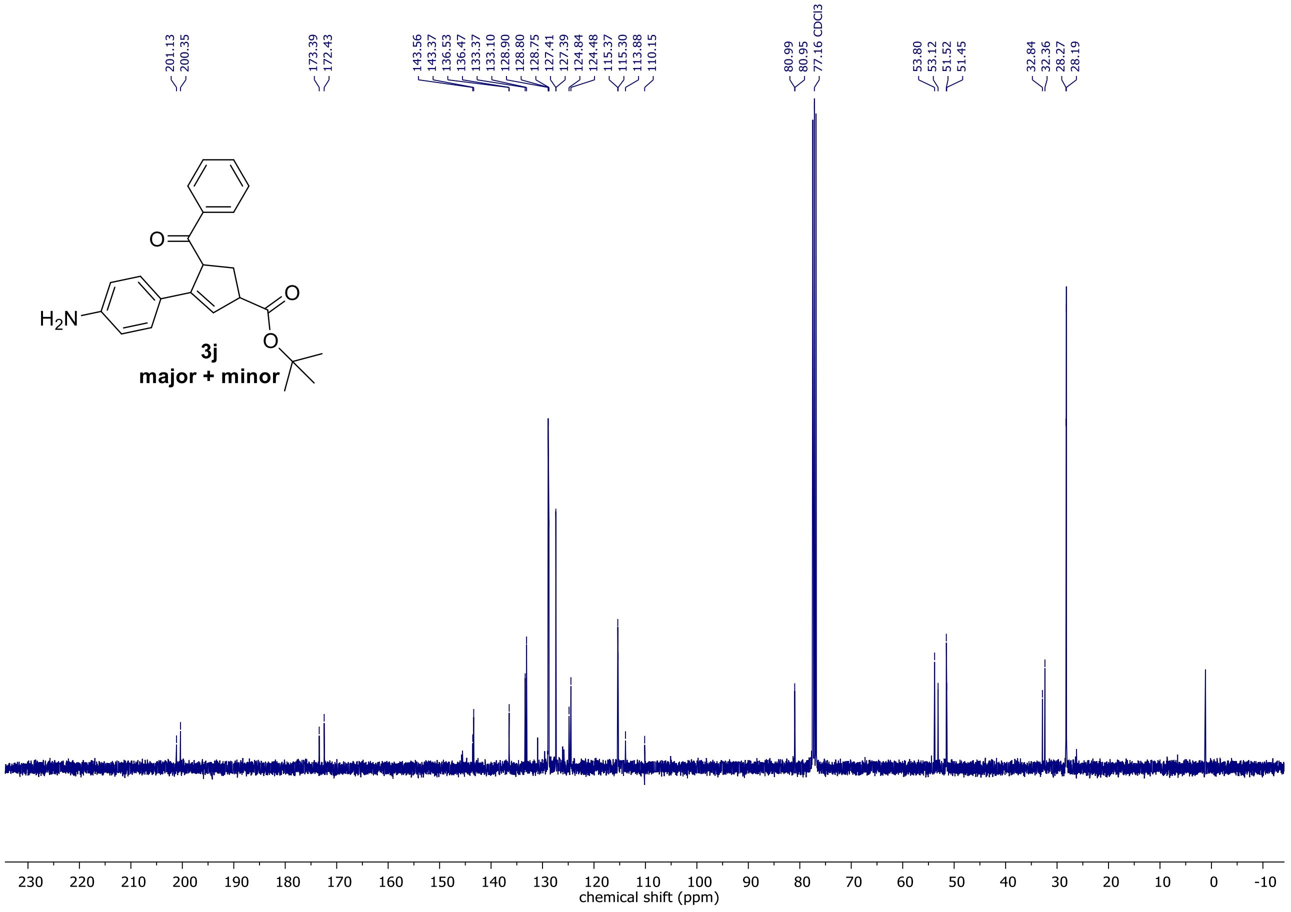

201.13 200.35 173.39 172.43 143.56 143.37 136.53 136.47 133.37 133.10 128.90 128.80 128.75 127.41 127.39 124.84 124.48 115.37 115.30 113.88 110.15 80.99 80.95 77.16 CDCl3 53.80 53.12 51.52 51.45 32.84 32.36 28.27 28.19
H2N
3j
major + minor
chemical shift (ppm)

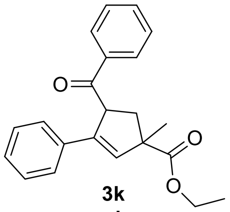

3k
major

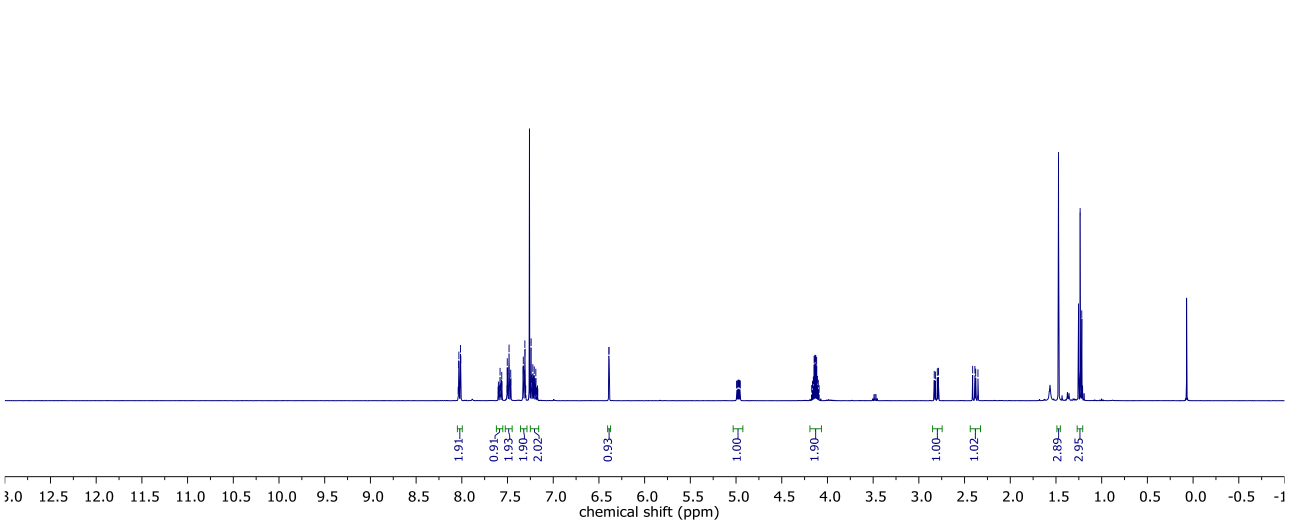

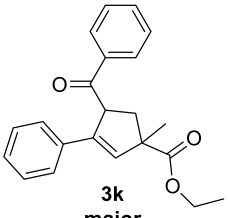

3k
major

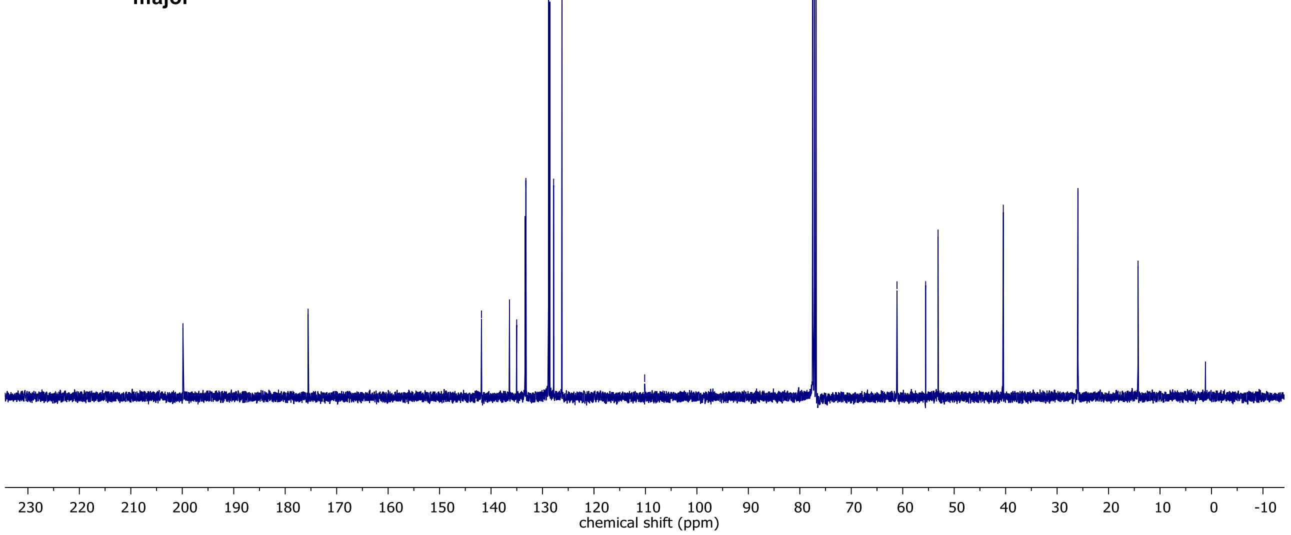

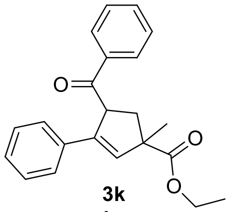

3k
minor

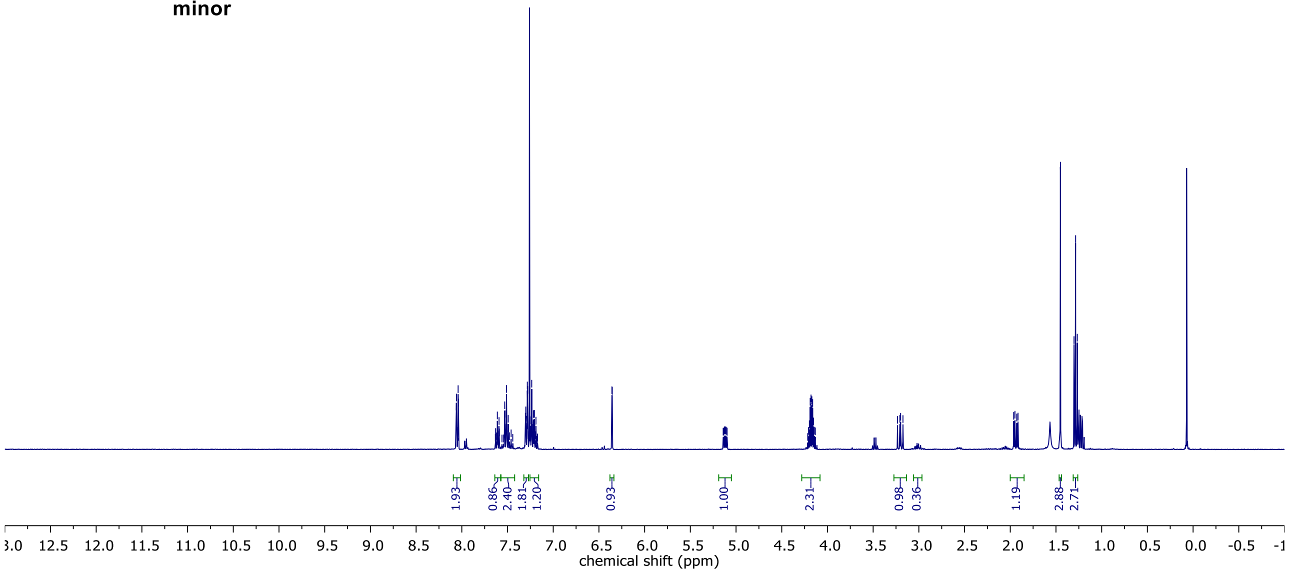
8.06 8.06 8.05 8.04 8.04 7.63 7.63 7.63 7.62 7.61 7.61 7.60 7.59 7.59 7.56 7.54 7.53 7.53 7.51 7.50 7.49 7.48 7.46 7.44 7.30 7.30 7.29 7.29 7.28 7.27 7.26 7.25 7.24 7.23 7.22 7.22 7.21 7.21 7.20 7.20 7.19 7.18 7.17 7.17 6.36 6.35 5.14 5.14 5.13 5.12 5.12 5.11 5.10 5.10 4.22 4.21 4.20 4.19 4.18 4.17 4.16 4.15 4.14 4.13 3.23 3.21 3.20 3.17 1.96 1.95 1.93 1.92 1.45 1.30 1.28 1.27
1.93
0.86
2.40
1.81
1.20
0.93
1.00
2.31
0.98
0.36
1.19
2.88
2.71
13.0 12.5 12.0 11.5 11.0 10.5 10.0 9.5 9.0 8.5 8.0 7.5 7.0 6.5 6.0 5.5 5.0 4.5 4.0 3.5 3.0 2.5 2.0 1.5 1.0 0.5 0.0 -0.5 -1
chemical shift (ppm)

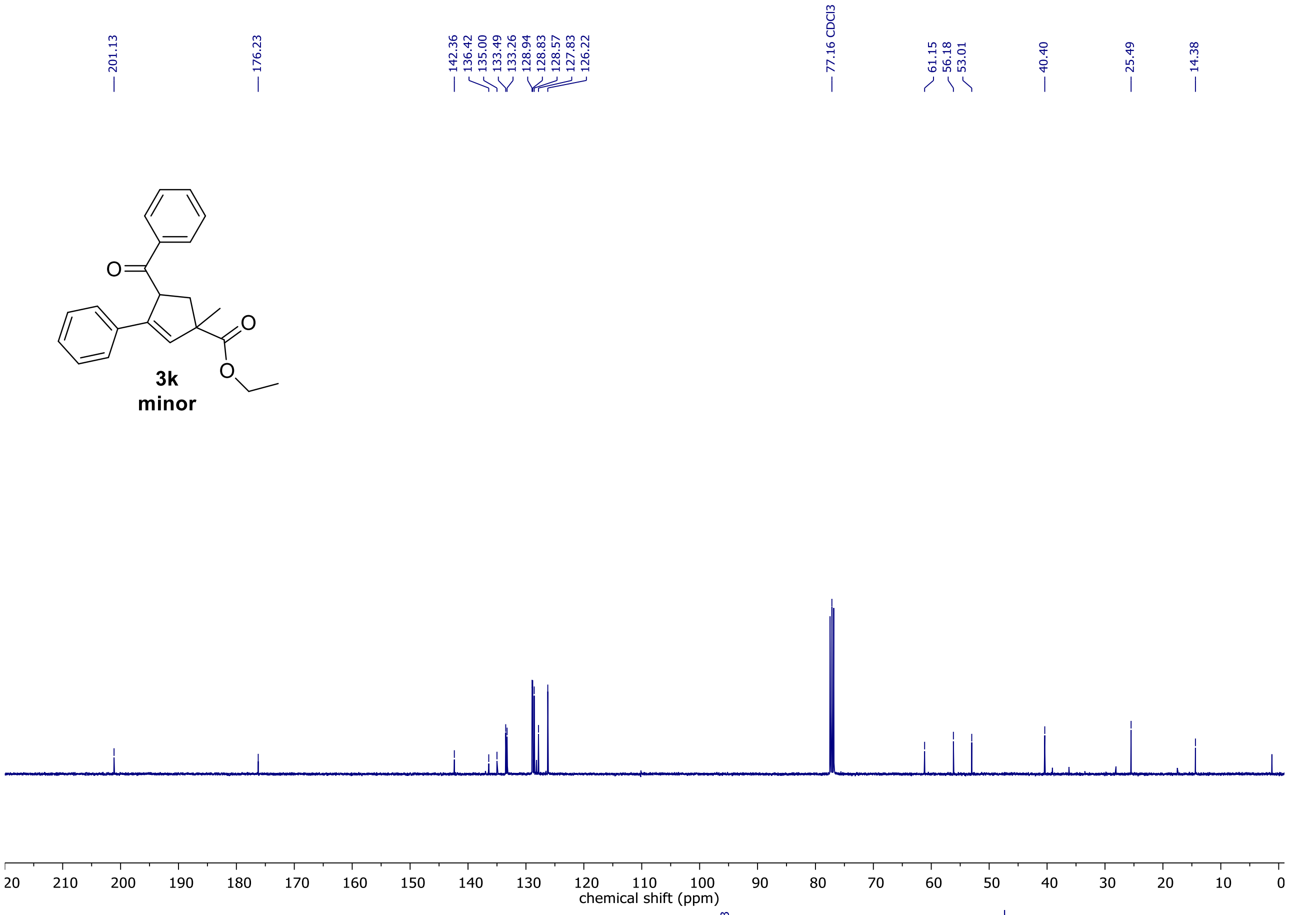
201.13
176.23
142.36
136.42
135.00
133.49
133.26
128.94
128.83
128.57
127.83
126.22
77.16 CDCl3
61.15
56.18
53.01
40.40
25.49
14.38
3k
minor
chemical shift (ppm)

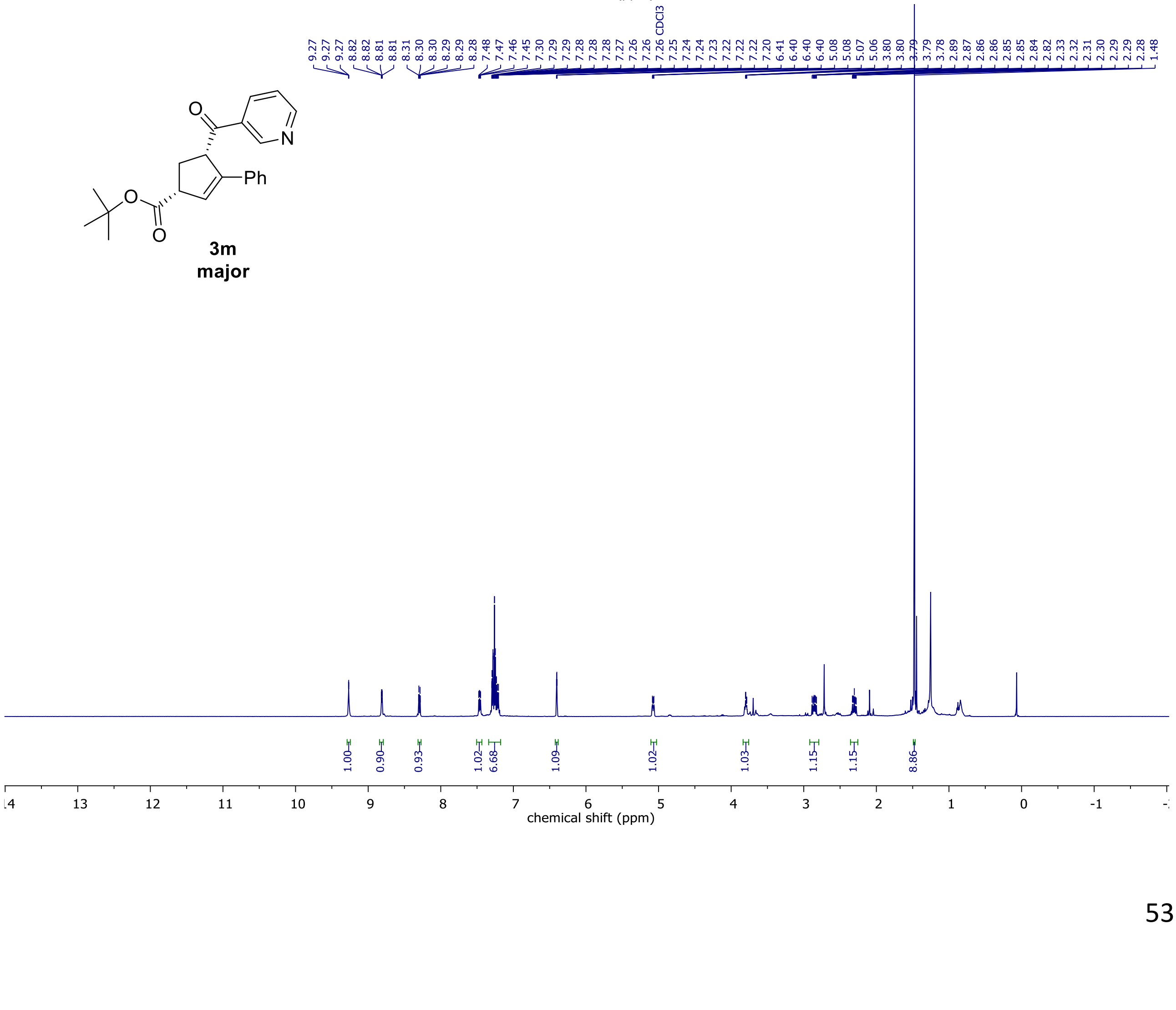
3m
major
chemical shift (ppm)

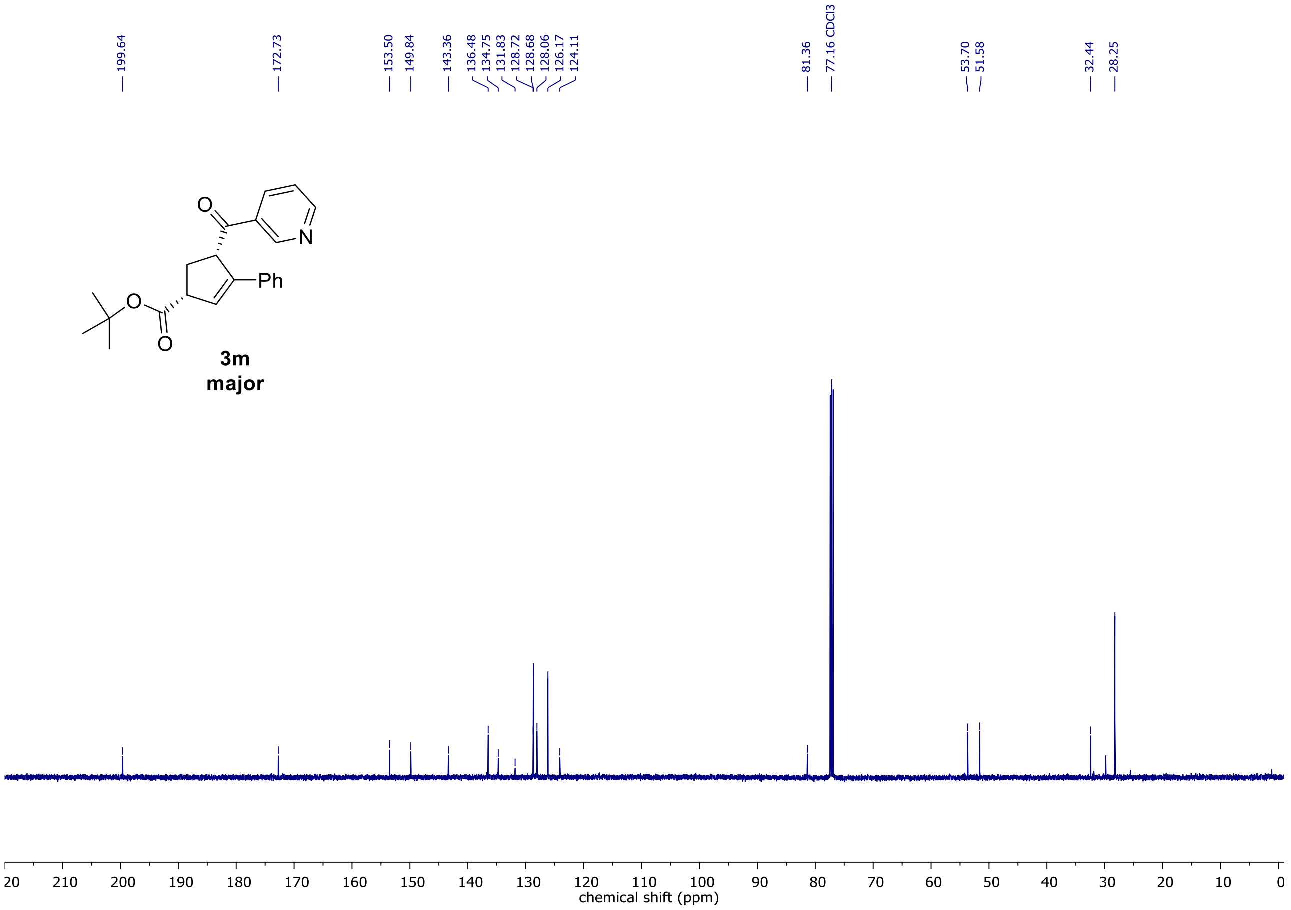
199.64
172.73
153.50
149.84
143.36
136.48
134.75
131.83
128.72
128.68
128.06
126.17
124.11
81.36
77.16 CDCl3
53.70
51.58
32.44
28.25
O
N
Ph
O
O
3m
major
20 210 200 190 180 170 160 150 140 130 120 110 100 90 80 70 60 50 40 30 20 10 0
chemical shift (ppm)

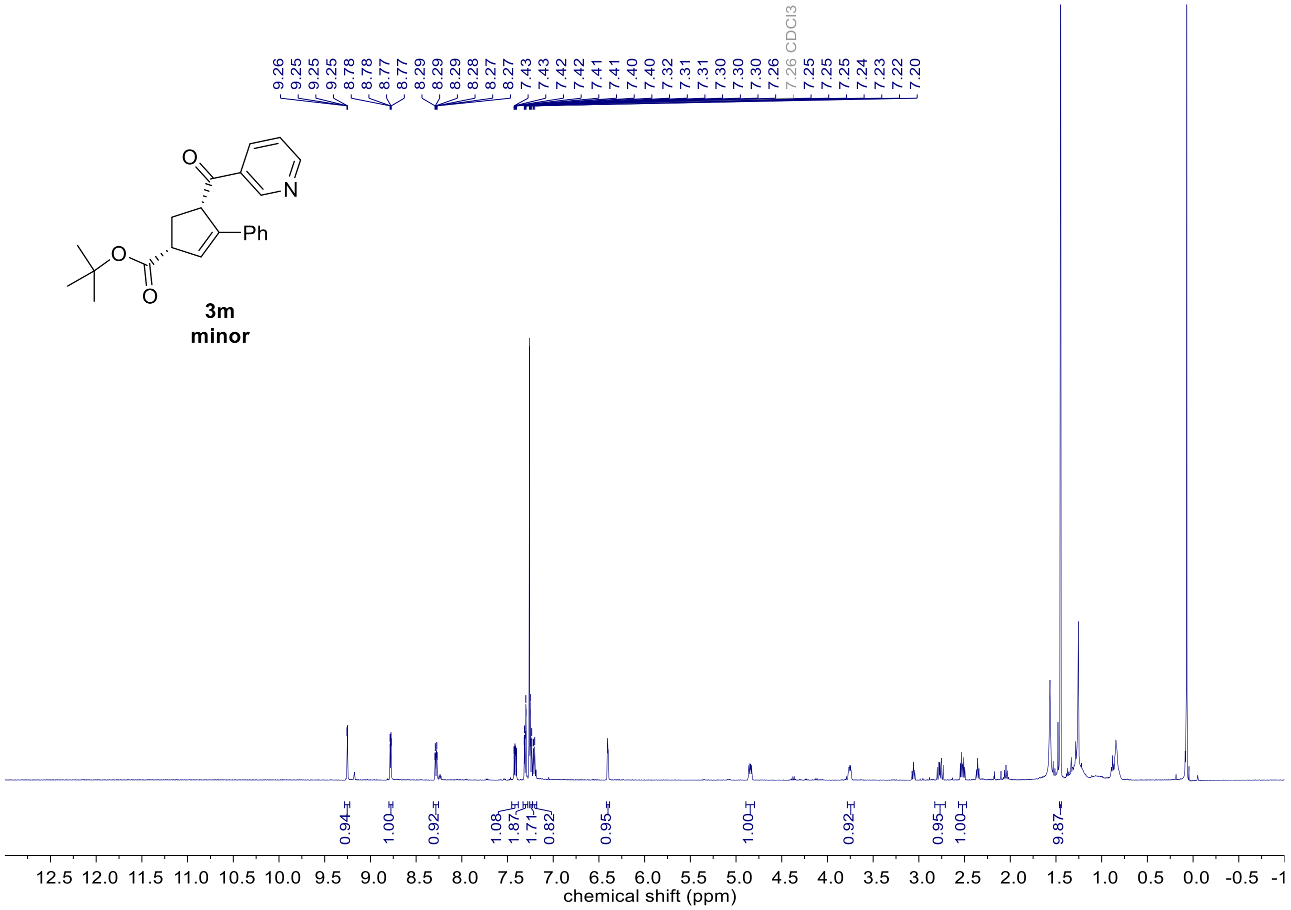

3m
minor

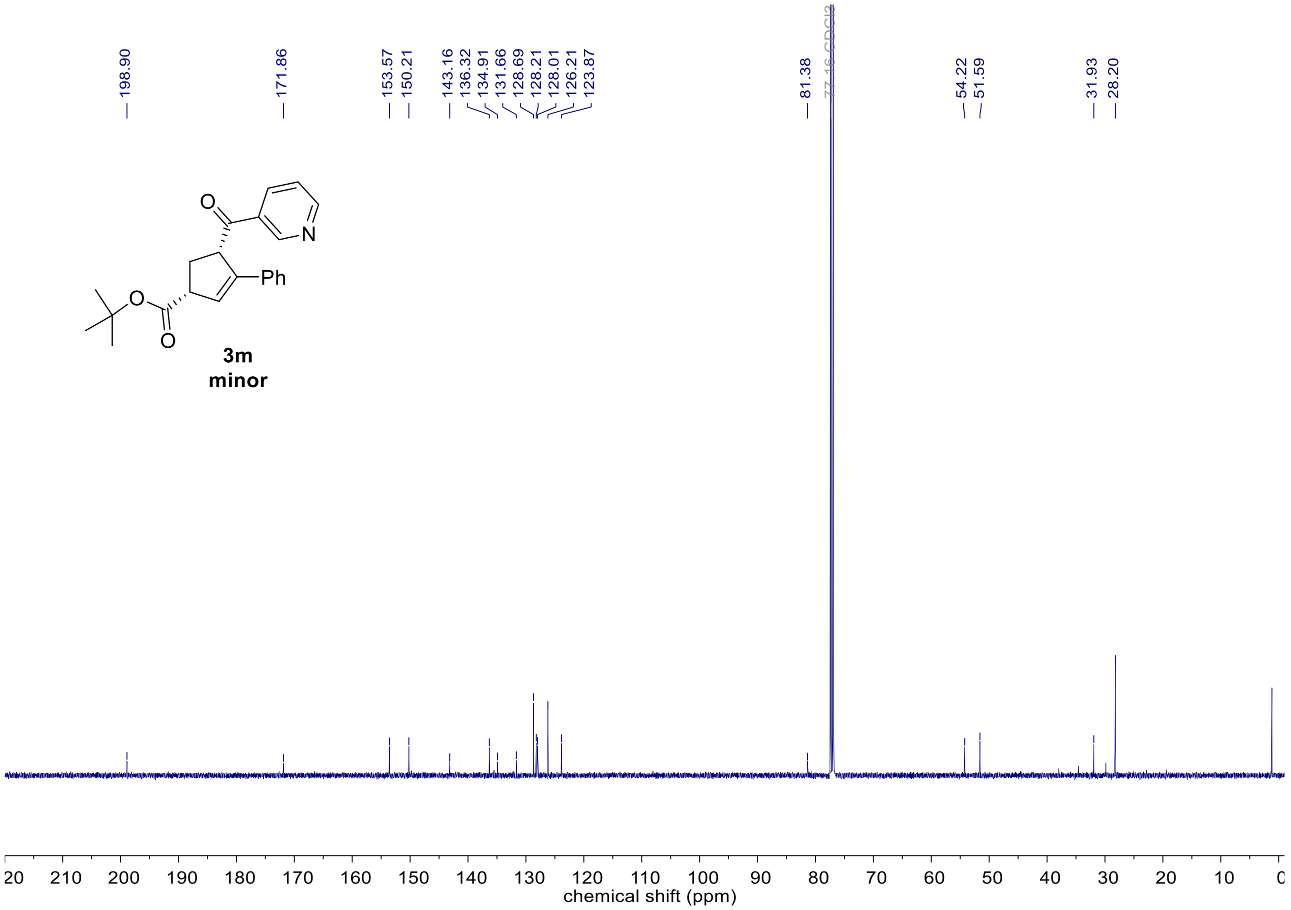

3m
minor

7.2 Bidimensional Spectra 3a (minor diastereoisomer)

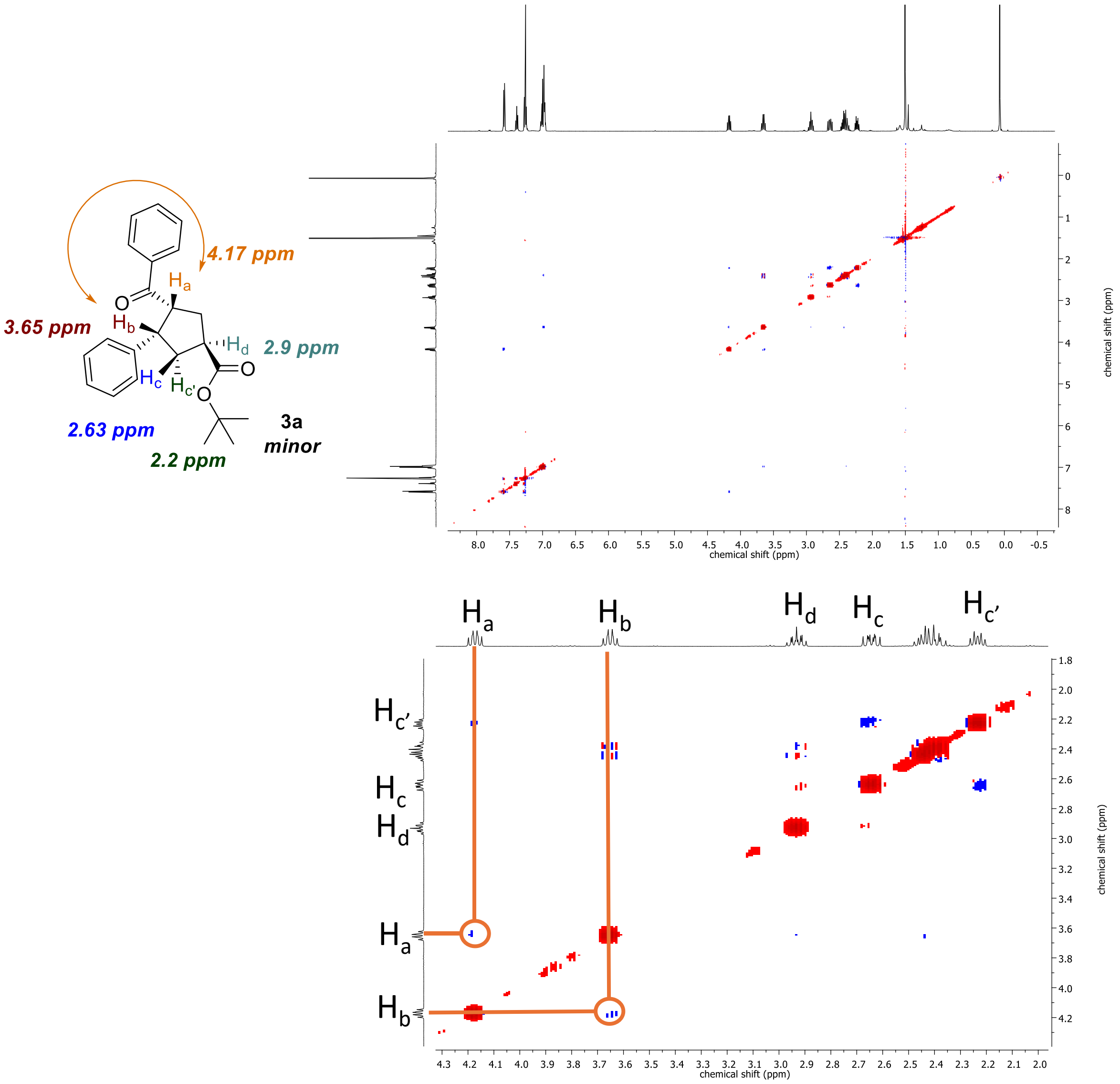

8. References

1. Amador, A.G., Sherbrook, E.M., and Yoon, T.P. (**2016**). Enantioselective Photocatalytic [3 + 2] Cycloadditions of Aryl Cyclopropyl Ketones. *J. Am. Chem. Soc. 138*, 4722–4725. https://doi.org/10.1021/jacs.6b01728.

2. Sun, W., Zhao, M., Meng, Y., Zheng, C., Yang, K., Wang, S., Ke, C., and Zhang, Z. (**2024**). Photoinduced [3 + 2] Cycloadditions of Aryl Cyclopropyl Ketones with Alkynes and Alkenes. *Org. Lett. 26*, 3762–3766. https://doi.org/10.1021/acs.orglett.4c00843.

3. Nguyen, T.V.T., Bossonnet, A., Wodrich, M.D., and Waser, J. (**2023**). Photocatalyzed [2σ + 2σ] and [2σ + 2π] Cycloadditions for the Synthesis of Bicyclo[3.1.1]heptanes and 5- or 6-Membered Carbocycles. *J. Am. Chem. Soc. 145*, 25411–25421. https://doi.org/10.1021/jacs.3c09789.
